# Investigating the Role of Glycolysis in Xuefu Zhuyu Capsule-Promoted Angiogenesis in Endothelial Cells: A Study Based on Network Pharmacology, Molecular Docking, and In Vitro Validation

**DOI:** 10.3390/ph18121902

**Published:** 2025-12-17

**Authors:** Fan Lin, Zhifeng Yao, Jiaming Yu, Xiaoqi Chen, Xinlei Chen, Yuxia Li, Juanli Fu, Ye Cheng, Junting Li, Chang Fang, Yizheng Wang, He Wang, Jing Cai

**Affiliations:** 1 College of Integrative Medicine, Fujian University of Traditional Chinese Medicine, Fuzhou 350122, China; 2009037@fjtcm.edu.cn (F.L.); 3230505010@fjtcm.edu.cn (Z.Y.); 18905907332@163.com (J.Y.);; 2 Key Laboratory of Integrative Medicine on Chronic Diseases, Fujian Province University, Fuzhou 350122, China

**Keywords:** angiogenesis, peripheral artery disease, Xuefu Zhuyu Capsule, endothelial cells, glycolysis, network pharmacology

## Abstract

**Background**: Peripheral artery disease (PAD) represents a major global cause of mortality and disability. A primary therapeutic strategy involves promoting angiogenesis in ischemic limbs. The Xuefu Zhuyu Capsule (XFZYC) is widely used in China for treating PAD and demonstrates therapeutic potential; however, the mechanism underlying its pro-angiogenic effect remains unclear. **Methods**: The components of XFZYC were identified via TCMSP and HERB databases, with network pharmacology and molecular docking predicting its potential targets and pathways. For in vitro validation, drug-containing serum and blank control serum were prepared. Human Microvascular Endothelial Cells (HMEC-1) cells were treated with 1.25%, 2.5%, or 5% serum to determine the optimal concentration using tube formation assays and Western blot (WB) analysis of HIF-1α, HK2, and PFKFB3. The efficacy of XFZYC was further assessed through CCK-8, scratch wound healing, cell adhesion, and tube formation assays. Glycolytic metabolite levels and enzyme activities were measured by colorimetric assays and WB. **Results**: Network pharmacology screening identified 167 active components in XFZYC and 2967 potential targets. GO functional and KEGG pathway enrichment analyses suggested that XFZYC likely promotes the glycolytic pathway via the HIF-1 signaling pathway, specifically mediated by HK2 and PFKFB3. In vitro experiments confirmed that XFZYC enhanced HMEC-1 cell viability, migration, adhesion, and tube formation. Concurrently, it augmented the glycolytic capacity of HMEC-1 cells, manifested by increased glucose consumption, lactate production, enhanced activity of key glycolytic enzymes (HK, PFK, and PK), and upregulated protein expression of PFKFB3. Treatment with 3PO, a glycolytic inhibitor, significantly suppressed these drug-induced effects. **Conclusions**: XFZYC promotes angiogenesis in endothelial cells by modulating the glycolytic pathway, an effect primarily mediated through the upregulation of PFKFB3 expression. This study offers a preliminary exploration of the underlying mechanisms by which XFZYC may act in the treatment of PAD, thereby providing a new scientific perspective for further understanding its therapeutic effects.

## 1. Introduction

PAD is a condition characterized by the narrowing or occlusion of arteries, primarily affecting those in the lower limbs. With approximately 200 million patients worldwide [1], PAD is the third most common manifestation of atherosclerotic disease, following coronary artery disease and stroke. Regardless of the presence of symptoms, individuals with PAD face a significantly higher risk of major adverse cardiovascular events and all-cause mortality compared to the non-affected population. A key therapeutic goal is achieving angiogenesis and blood supply reconstruction [2,3]. Therapeutic angiogenesis, the process of forming new blood vessels based on pre-existing vasculature, is crucial for improving blood supply to ischemic tissues [4,5]. A series of pioneering studies in recent years has confirmed that glycolysis not only provides energy for endothelial cells (ECs) but also comprehensively regulates various cellular behaviors during angiogenesis, such as proliferation, migration, adhesion, and tube formation. 6-phosphofructo-2-kinase/fructose-2,6-biphosphatase 3 (PFKFB3), a key regulator of glycolysis, has been identified as a critical modulator of angiogenesis [6,7]. Therapeutic angiogenesis strategies targeting glucose metabolism show promising application prospects, offering new avenues for intervening in metabolism to treat PAD [8,9].

The pursuit of such therapeutic strategies must, however, account for the inherent complexity of vascular repair. Recent evidence underscores that effective revascularization likely necessitates multi-targeted interventions capable of coordinating complementary signaling axes. For instance, the synergistic interplay between VEGF and SDF-1α has been shown to enhance the function of both endothelial progenitor and vascular smooth muscle cells, highlighting the power of coordinated signaling in vascular regeneration [10]. This complexity is mirrored in persistent clinical challenges, such as combating endothelial injury and microvascular dysfunction in conditions like thrombotic microangiopathy [11]. In this context, network pharmacology offers a compelling framework for understanding traditional herbal formulas, which are inherently multi-component agents. This approach is validated by examples such as Taohong Siwu Decoction, which ameliorates vascular pathology via coordinated multi-target mechanisms [12].

Meanwhile, advancements in technologies such as capillary electrophoresis are enabling in-depth characterization of complex herbal interactions [13]. The scientific foundation is further strengthened by a deepening understanding of metabolic regulatory networks: research confirms that endothelial homeostasis is fundamentally governed by its metabolic microenvironment. For instance, active compounds like orientin modulate cellular metabolic processes to inhibit macrophage pyroptosis [14], while the deacetylase SIRT6 acts as a key metabolic sensor and regulator, directly protecting endothelial function from inflammatory stress [15]. These findings collectively demonstrate that the metabolic state of endothelial cells is a central determinant of their homeostasis and governs cellular responsiveness to external stimuli [16]. These converging insights establish a theoretical foundation for exploring sophisticated multi-target therapies from the fundamental perspective of metabolic regulation.

Xuefu Zhuyu Decoction, originating from Wang Qingren’s “Corrections on Errors in Medical Classics,” is a representative formula of the method to promote blood circulation and remove blood stasis [17]. The Xuefu Zhuyu Capsule (XFZYC) is a modern preparation derived from the decoction, sharing its core herbal composition and therapeutic effects [18,19]. In clinical practice, XFZYC is widely used for preventing and treating conditions such as myocardial ischemia in coronary heart disease, atherosclerosis, and chronic limb ischemia, with notable efficacy [20,21]. Experimental studies indicate that under specific conditions, it can upregulate Dll4 expression, activate the Notch signaling pathway [22,23,24], and interact with various pro-angiogenic and anti-angiogenic factors, thereby regulating angiogenesis. Given that angiogenesis is highly energy-dependent and requires metabolic reprogramming in endothelial cells, the potential link between XFZYC’s pro-angiogenic effects and cellular metabolism is still unclear. Our study was designed to address this unanswered question, and for the first time, we specifically investigated the connection between XFZYC’s therapeutic actions and endothelial glycolytic metabolism to elucidate a new mechanism.

Here, to bridge this gap, we integrate network pharmacology prediction with molecular docking and rigorous in vitro validation. We hypothesize that XFZYC promotes angiogenesis by directly upregulating endothelial glycolysis, with PFKFB3 serving as a central target. By linking the pharmacological action of a classic herbal formula to the modern metabolic theory of angiogenesis, our work aims to uncover a previously unknown mechanistic layer of its therapeutic action, providing a fresh scientific perspective for its application in ischemic diseases.

## 2. Results

### 2.1. Bioinformatics Analysis of Peripheral Artery Disease

#### 2.1.1. Targets Associated with Peripheral Artery Disease

**Figure 1:**
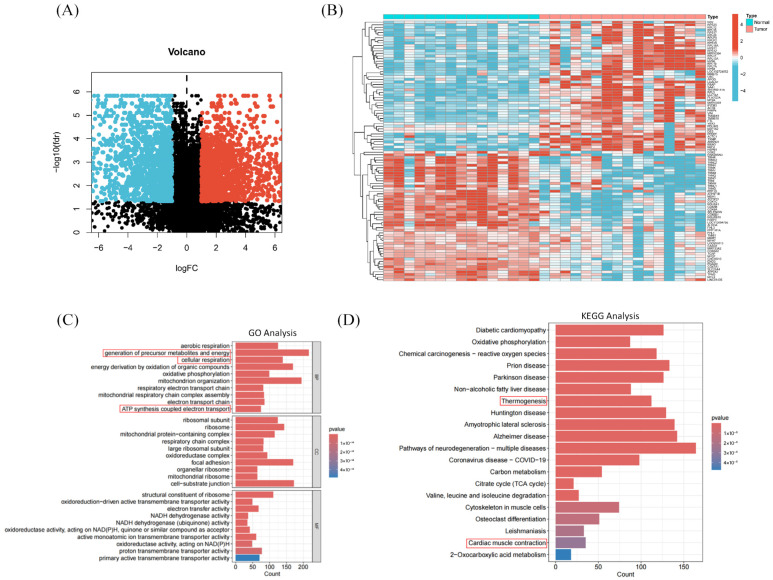
Transcriptomic Analysis of Peripheral Artery Disease using the GEO dataset GSE120642. (**A**) Volcano plot of differentially expressed genes (DEGs) between patients with severe limb ischemia (*n* = 16) and healthy controls (*n* = 15). Significantly dysregulated genes (|log2FC| > 1; adjusted *p*-value < 0.05) are highlighted. Significantly upregulated genes are shown in red, downregulated genes in blue, and non-significant genes in black. (**B**) Heatmap of the top 50 upregulated and top 50 downregulated DEGs across all samples. Significantly upregulated genes are colored red, and downregulated genes are colored blue, illustrating the expression patterns in patients with severe limb ischemia compared to healthy conditions. (**C**) Bar graph showing the significant enrichment terms (FDR < 0.05) for Biological Process (BP), Cellular Component (CC), and Molecular Function (MF) obtained from GO enrichment analysis. Bar height represents the enrichment significance (−log10 (*p*-value)); color indicates the category (BP: blue; MF: red). (**D**) Bar plot of the top 20 enriched KEGG pathways. The X-axis represents the gene ratio, while the Y-axis lists the pathway names. Bar height corresponds to gene count, and color intensity indicates the −log10 (*p*-value).

RNA-seq data were obtained from the GEO database, comprising samples from 15 healthy elderly individuals without PAD and 16 patients with severe limb ischemia undergoing amputation [25]. A total of 3759 differentially expressed genes (DEGs) were identified when comparing the normal and disease groups, consisting of 1879 upregulated and 1880 downregulated genes (Figure 1A,B). These DEGs were considered potential targets for PAD and used for subsequent analysis.

#### 2.1.2. Gene Functional Enrichment Analysis of PAD-Associated Targets

Functional enrichment analysis was performed on the PAD-related targets. For Biological Process (BP) terms, significant enrichments were primarily related to mitochondrial respiratory chain and energy metabolism processes, especially pathways associated with aerobic respiration, cellular respiration and energy generation, oxidative phosphorylation, electron transport, and ATP synthesis. Cellular Component (CC) enrichments were mainly mitochondrial complexes involved in respiration and translation, particularly respiratory chain complexes, ribosomes, and their subunits. Molecular Function (MF) enrichments involved functions related to electron transfer, oxidoreductase activity, ion transport, and ribosomal structure (Figure 1C). KEGG pathway analysis of PAD-related target genes included Oxidative phosphorylation, Carbon metabolism, Citrate cycle (TCA cycle), 2-Oxocarboxylic acid metabolism, Valine, leucine and isoleucine degradation, among more than 20 other pathways (Figure 1D). These pathways are highly relevant to the core pathology of disrupted cellular energy (ATP) supply due to ischemia.

### 2.2. Network Pharmacology Analysis

#### 2.2.1. Active Compounds in Xuefu Zhuyu Capsule and Target Prediction

A total of 167 active compounds were screened from XFZYC (Table 1), including 13 from Chaihu, 15 from Chishao, 4 from Chuanxiong, 2 from Danggui, 86 from Gancao, 11 from Honghua, 2 from Jiegeng, 8 from Niuxi, 3 from Shengdihuang, 19 from Taoren, and 4 from Zhiqiao. For the 11 studied herbs, a total of 2967 potential action targets were predicted (Table 1), comprising 374 for Chaihu, 195 for Chishao, 253 for Chuanxiong, 44 for Danggui, 775 for Gancao, 343 for Honghua, 101 for Jiegeng, 408 for Niuxi, 55 for Shengdihuang, 251 for Taoren, and 168 for Zhiqiao (Supplementary Table S1).

#### 2.2.2. Drug-Compound-Target Network Construction and Analysis

**Figure 2:**
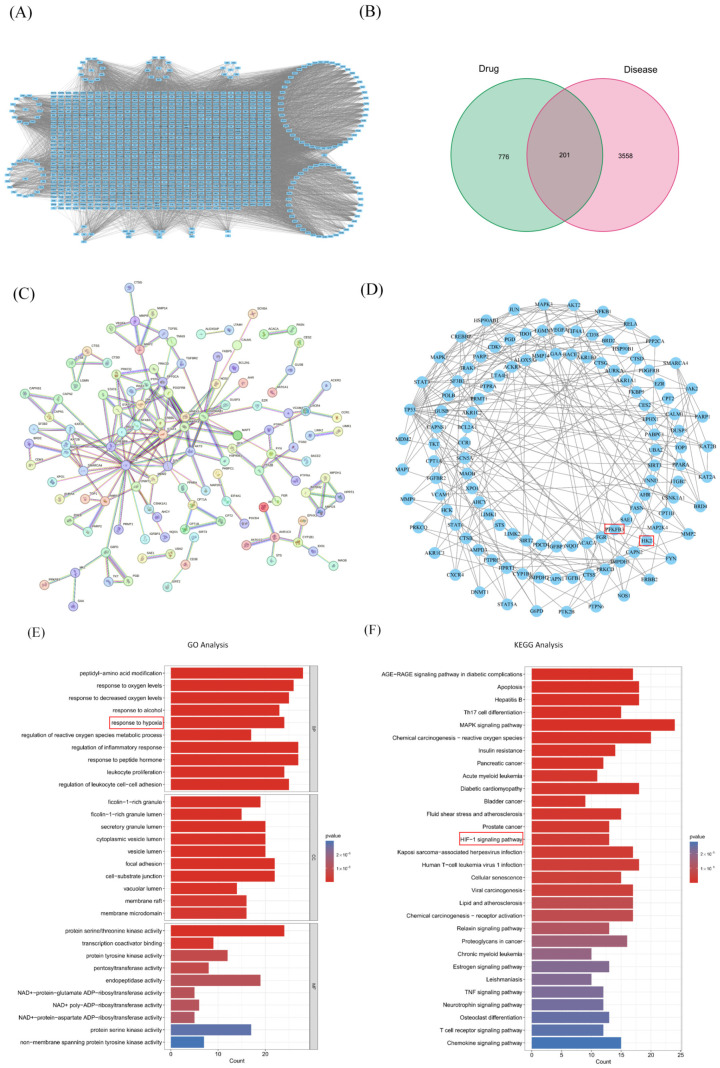
Network Pharmacology Analysis Predicting the Potential Mechanism of Action for XFZYC in Treating PAD. (**A**) Active component-Potential target interaction network of XFZYC. Nodes represent active components and action targets; edges represent interactions. This network illustrates the multi-component, multi-target systematic characteristics of the formula. (**B**) Venn diagram of XFZYC active components and PAD targets. The intersecting targets are considered potential direct targets for XFZYC in treating PAD. (Drug target:green;Disease target:pink;Common Targets:grey) (**C**) Protein–Protein Interaction (PPI) network diagram, showing the interaction relationships among the core targets of PAD. (**D**) Topological analysis of XFZYC-PAD common targets, identifying key targets in the network based on “node degree value”. The targets marked with red square are the primary focus of this paper. (**E**) Gene Ontology (GO) functional enrichment analysis bar graph, showing the significantly enriched Biological Processes (BP), Molecular Functions (MF), and Cellular Components (CC) for the potential targets.The pathways marked with red squares are the primary focus of this paper. (**F**) Kyoto Encyclopedia of Genes and Genomes (KEGG) pathway enrichment analysis presented as a bar plot. The pathways marked with red squares are the primary focus of this paper.

The interaction network between active components in XFZYC and their targets was visualized and analyzed using Cytoscape version 3.7.1, generating a comprehensive network comprising 1127 nodes (including 959 targets, 157 active components, and 11 herbs) and 10,321 edges (Figure 2A). Venn diagram analysis identified 201 common target genes shared between the drug and PAD target genes (Figure 2B). These 201 common target genes were input into the STRING database, and a PPI network was generated after removing discrete nodes (Figure 2C), resulting in 207 nodes. Topological analysis screened for targets with degree, closeness, and betweenness centrality values greater than their respective means (Degree > 9.3708, Closeness > 0.0357, Betweenness > 315.2359) as core targets (Figure 2D). The results demonstrate that these targets are associated with multiple pivotal processes, including energy metabolism, inflammatory and immune responses, angiogenesis, and the regulation of cell proliferation and apoptosis. Among them, the key glycolytic enzymes HK2 and PFKFB3 are identified as potential critical targets for XFZYC in regulating cellular metabolism, given their central regulatory role in energy metabolism [26,27].

#### 2.2.3. GO Functional and KEGG Pathway Enrichment Analysis of Drug-Disease Intersection Targets

GO and KEGG analyses were performed on the targets associated with XFZYC’s treatment of PAD. GO enrichment analysis revealed that Biological Process (BP) terms were significantly enriched in cellular perception and response to hypoxia, oxidative stress, and hormonal and nutrient signals, particularly the response to hypoxia and the regulation of reactive oxygen species metabolic processes. Cellular Component (CC) terms were enriched in junctions connecting cells to the external environment and specific microdomains on the cell membrane for signaling. Molecular Function (MF) terms were enriched in functions involving phosphorylated histones and other proteins, suggesting their key roles in epigenetic regulation, DNA damage response, and cell signal transduction (Figure 2E). KEGG pathway analysis indicated that the top 20 relevant pathways mainly included the MAPK signaling pathway, HIF-1 signaling pathway, AGE-RAGE signaling pathway in diabetic complications, Citrate cycle (TCA cycle), Chemical carcinogenesis—reactive oxygen species, T-cell receptor signaling pathway, etc. (Figure 2F). This set of target genes collectively exerts its primary functions by participating in core signaling pathway networks such as AGE-RAGE, MAPK, and HIF-1.

The enrichment analysis of PAD target genes from Section 2.1.2 suggested a close relationship between PAD and tissue ischemia/hypoxia, with one of the core pathological consequences being disrupted ATP supply, highlighting the importance of energy metabolism dysfunction caused by ischemia in disease pathogenesis and progression (Figure 2E,F). Regarding the molecular mechanism, the Hypoxia-Inducible Factor-1 (HIF-1) pathway serves as a hub connecting hypoxia and metabolic remodeling: its functional subunit HIF-1α acts as a “master switch,” capable of directly transcriptionally regulating the expression of key glycolytic genes such as PFKFB3 [28].

Based on the above evidence, we hypothesize that the HIF-1 pathway plays a key role in XFZYC’s targeting of glycolysis for treating PAD [29].

### 2.3. Molecular Docking

**Figure 3:**
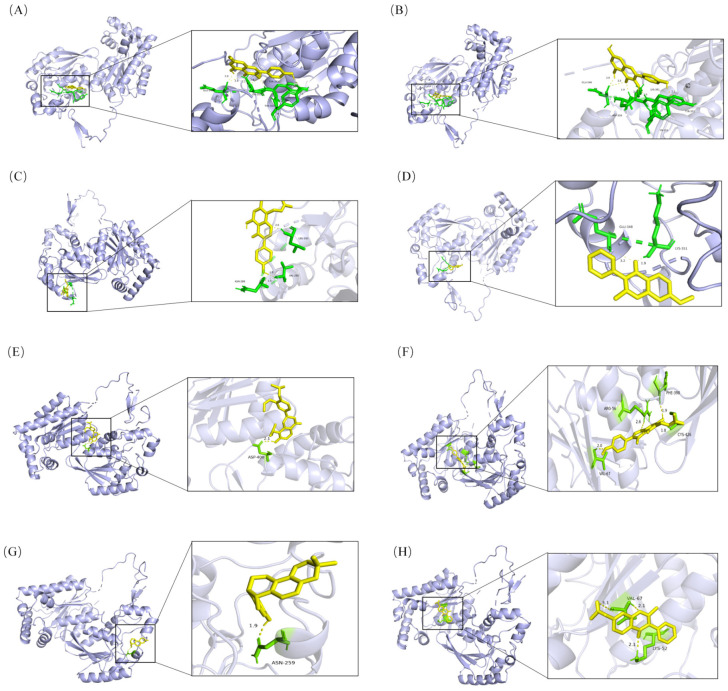
Molecular Docking Visualization of Key Active Components from XFZYC with PFKFB3. (**A**) Kaempferol (MOL000422), (**B**) Jaranol (MOL000239), (**C**) Lupiwighteone (MOL003656), (**D**) 7-Methoxy-2-methyl isoflavone (MOL003896), (**E**) Euchrenone (MOL004806), (**F**) Gancaonin A (MOL004856), (**G**) Shinpterocarpin (MOL004891), (**H**) 7-Acetoxy-2-methylisoflavone (MOL004991). In the figure, steel blue denotes the PFKFB3 protein, yellow represents the small-molecule compound, and green indicates the specific docking site.

Systematic molecular docking of XFZYC constituents against PFKFB3, HIF-1α, and HK2 revealed robust interactions and stable complex formation for a majority of the ligands (Appendix A Table A1). Strikingly, PFKFB3 emerged as a predominant target, with 30 out of 34 constituents exhibiting binding free energies below −5 kcal/mol. Notably, a subset of eight PFKFB3-targeting constituents—Kaempferol (MOL000422), Jaranol (MOL000239), Lupiwighteone (MOL003656), 7-Methoxy-2-methyl isoflavone (MOL003896), Euchrenone (MOL004806), Gancaonin A (MOL004856), Shinpterocarpin (MOL004891), and 7-Acetoxy-2-methylisoflavone (MOL004991)—demonstrated superior affinity, with binding energies below −6.5 kcal/mol (Table 2 and Figure 3). Significant binding was also observed for HIF-1α (3 constituents) and, remarkably, for HK2, where the single screened constituent, Rehmaglutin C, also achieved a binding energy <−6.5 kcal/mol. These findings indicate that XFZYC is a rich source of high-affinity ligands for PFKFB3, containing abundant potent PFKFB3 effectors.

### 2.4. Effects of XFZYC-Containing Serum on In Vitro Tube Formation Ability and HIF-1α, HK2, and PFKFB3 Protein Expression in Endothelial Cells

**Figure 4:**
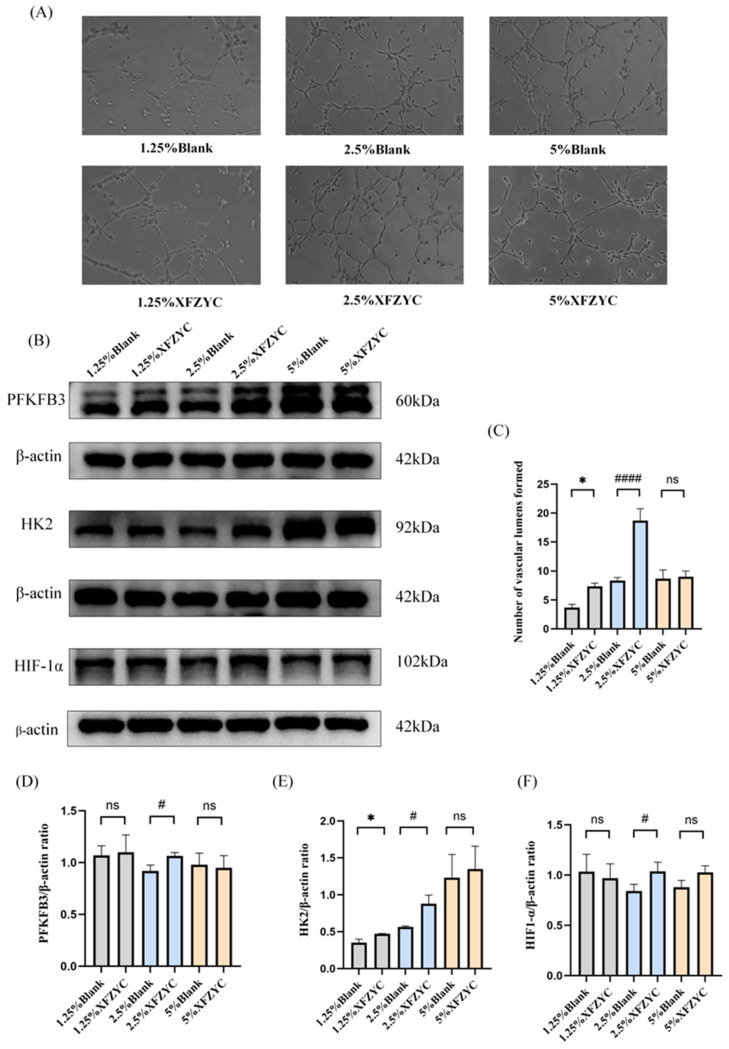
Effects of different concentrations of Xuefu Zhuyu Capsule drug-containing serum on endothelial cell tube formation capacity and HIF-1α, HK2, and PFKFB3 protein expression. (**A**,**C**) Tube formation assay detecting the in vitro tube formation capacity of endothelial cells treated with different concentrations of XFZYC drug-containing serum. (**B**,**D**,**E**,**F**) Protein expression levels of PFKFB3, HK2, and HIF-1α under treatment with 1.25%, 2.5%, and 5% blank serum or drug-containing serum were determined by Western blot analysis. * *p* < 0.05 for the 1.25% XFZYC group compared to the 1.25% Blank group; ^#^ *p* < 0.05, ^####^ *p* < 0.0001 for the 2.5% XFZYC group compared to the 2.5% Blank group, ns, not significant; *n* = 3.

To determine the appropriate drug concentration for subsequent experiments, an in vitro tube formation assay was performed. The results indicated that 2.5% XFZYC-containing serum promoted a stronger pro-angiogenic capacity compared to lower (1.25%) and higher (5%) concentrations (Figure 4A,C). Western blot (WB) analysis was then conducted to validate the predictions from the network pharmacology and molecular docking studies. The WB results showed that both 1.25% and 2.5% XFZYC-containing serum promoted the protein expression of HIF-1α, HK2, and PFKFB3, whereas 5% XFZYC-containing serum showed inhibitory or non-statistically significant effects. Based on these findings, 2.5% XFZYC-containing serum was selected for use in all subsequent mechanistic investigations (Figure 4B,D–F).

### 2.5. Effects of XFZYC-Containing Serum and the Glycolytic Inhibitor 3PO on Endothelial Cell Angiogenic Behavior

**Figure 5:**
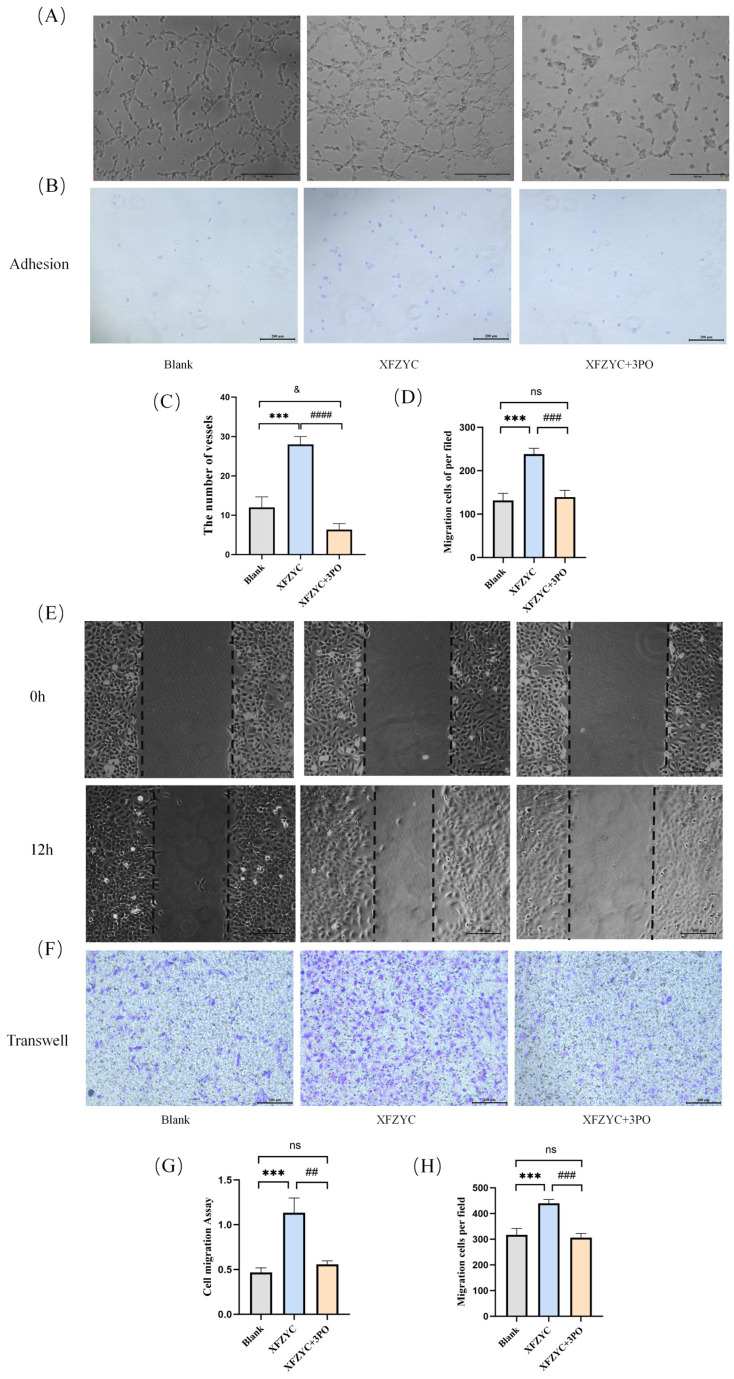
Effects of XFZYC-containing serum and the glycolytic inhibitor 3PO on endothelial cell angiogenic behavior. (**A**,**C**) Tube formation assay detecting the effects of XFZYC-containing serum and 3PO on the tube-forming capacity of endothelial cells. (**B**,**D**) Adhesion assay detecting the effects of XFZYC-containing serum and 3PO on the adhesive capacity of endothelial cells. (**E**,**F**,**G**,**H**) Scratch wound healing assay and Transwell assay detecting the effects of XFZYC-containing serum and 3PO on the migratory capacity of endothelial cells. *** *p* < 0.001 for the XFZYC group compared to the Blank group; ^##^ *p* < 0.01 ^###^ *p* < 0.001, ^####^ *p* < 0.0001 for the XFZYC + 3PO group compared to the XFZYC group; ^&^ *p* < 0.05 for the XFZYC + 3PO group compared to the Blank group; ns, not significant; *n* = 3.

The glycolytic inhibitor 3PO significantly inhibited endothelial cell angiogenic behavior. As shown in Figure 5A,C, XFZYC treatment significantly increased the number of in vitro tubes formed by HMEC-1 cells compared to the blank serum group. However, the addition of 3PO markedly attenuated this XFZYC-induced tube-forming capacity, indicating that the pro-angiogenic effect of XFZYC depends on the activation of the glycolytic pathway.

In the cell adhesion assay (Figure 5B,D), XFZYC significantly enhanced the adhesive ability of HMEC-1 cells. This effect was completely reversed by co-treatment with 3PO, with no statistically significant difference observed between the XFZYC + 3PO group and the blank serum group (*p* > 0.05). These results suggest that XFZYC promotes cell adhesion by enhancing glycolysis, an effect that can be inhibited by 3PO.Regarding cell migration, the scratch wound healing assay results (Figure 5E,G) demonstrated that XFZYC significantly promoted cell migration, whereas 3PO considerably weakened this promotive effect. The Transwell assay results further supported this conclusion (Figure 5F,H): XFZYC significantly increased the number of migrating cells, and this effect was significantly suppressed by co-treatment with 3PO, indicating that glycolysis plays a key role in XFZYC-induced cell migration.

In summary, the glycolytic pathway plays a crucial role in the process of XFZYC-promoted angiogenesis in endothelial cells, and its regulatory effect spans multiple key steps, including tube formation, adhesion, and migration.

### 2.6. Effects of XFZYC-Containing Serum and the Glycolytic Inhibitor 3PO on Glycolysis in Endothelial Cells

**Figure 6:**
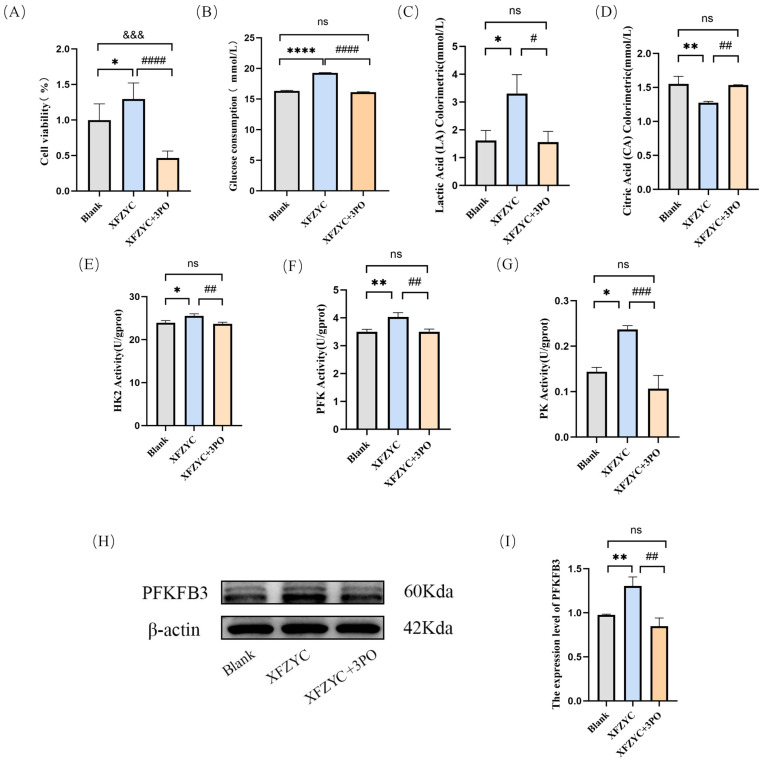
Effects of XFZYC-containing serum and the glycolytic inhibitor 3PO on glycolysis in endothelial cells. (**A**) Cell viability was determined by CCK-8 assay. (**B**) Glucose uptake was measured using a Glucose Assay Kit. (**C**) Lactate production was detected using a Lactate Assay Kit. (**D**) Citrate level was assessed using a Citrate Assay Kit. (**E**) HK enzyme activity was measured with a Hexokinase Activity Assay Kit. (**F**) PFK enzyme activity was determined using a Phosphofructokinase Activity Assay Kit. (**G**) PK enzyme activity was assessed with a Pyruvate Kinase Activity Assay Kit. (**H**,**I**) PFKFB3 protein expression was determined by Western blot analysis. * *p* < 0.05, ** *p* < 0.01, **** *p* < 0.0001vs. Blank group; ^#^ *p* < 0.05, ^##^ *p* < 0.01, ^###^ *p* < 0.001, ^####^ *p* < 0.001 vs. XFZYC group; ^&&&^ *p* < 0.001 vs. XFZYC + 3PO group; ns, not significant; *n* = 3.

The CCK-8 assay results (Figure 6A) showed that compared to the blank serum group, XFZYC-containing serum significantly promoted HMEC-1 cell viability. This promotive effect of XFZYC on HMEC-1 cell viability was markedly inhibited by pre-treatment with 3PO.

Compared to the blank serum group (Figure 6B–D), XFZYC treatment significantly promoted glucose uptake and lactate production in HMEC-1 cells, while simultaneously reducing the content of citrate, a key metabolite of the tricarboxylic acid (TCA) cycle. These changes were all reversed by pre-treatment with 3PO, indicating that XFZYC promotes cellular glycolysis and inhibits the TCA cycle.

Further investigation into the activities of key glycolytic enzymes revealed (Figure 6E–G) that XFZYC significantly increased the activities of hexokinase (HK), phosphofructokinase (PFK), and pyruvate kinase (PK). Conversely, 3PO effectively blocked the activating effects of XFZYC on these three enzymes, suggesting that XFZYC enhances the cell’s glycolytic energy supply capacity likely by augmenting the activities of key glycolytic enzymes. Furthermore, Western blot analysis showed that compared to the blank serum group (Figure 6H,I), PFKFB3 protein expression was increased in the XFZYC group. Pre-treatment with 3PO subsequently decreased PFKFB3 protein expression relative to the XFZYC-containing serum group. This indicates that XFZYC likely promotes glycolytic metabolism in HMEC-1 cells by upregulating PFKFB3 expression.

## 3. Discussion

The regulation of angiogenesis by Xuefu Zhuyu Decoction (XFZYD) exhibits multi-target and multi-pathway characteristics. XFZYD is ingeniously derived from the combination of Taohong Siwu Decoction and Sini Powder, effectively integrating the principles of promoting blood circulation to remove blood stasis and moving qi to relieve depression [17]. In the formula, Semen Persicae (Taoren) and Flos Carthami (Honghua) serve as the sovereign medicines; their combination specifically addresses stasis in the blood, capable of breaking stagnation to unblock the channels and also eliminating deep-seated stasis. Rhizoma Chuanxiong (Chuanxiong), pungent and potent in moving, can propel the stagnant qi in the blood, while Radix Paeoniae Rubra (Chishao), sour and cold, enters the liver and is skilled at dispersing extravasated blood. Combined with Radix Achyranthis Bidentatae (Niuxi) to guide the stasis downward, these three minister drugs work synergistically to assist the sovereign medicines in breaking up blood stasis. The entire formula prioritizes breaking stasis while concurrently regulating qi and nourishing blood, subtly aligning with the ancient tenet that “when qi moves, blood flows.”

Network pharmacology analysis revealed the multi-component, multi-target regulatory characteristics of XFZYC in treating PAD. This study screened 167 active components involving 2967 potential targets. The constructed component-target network suggests a high degree of systemic action characteristic of this formula. The enrichment analysis results indicated that one of the core pathologies of PAD is the disruption of ATP supply caused by ischemia and hypoxia, and that the HIF-1 signaling pathway, as a central hub connecting hypoxia and metabolic remodeling, likely plays a key role. Based on this theoretical framework, a key finding from our further research is that XFZYC likely targets pivotal points in the glycolytic pathway to intervene in PAD, with a focus on PFKFB3 as a core regulatory target. Gene Ontology enrichment analysis further indicated that the potential mechanisms of action of XFZYC are closely related to biological processes such as the cellular response to hypoxia and oxidative stress, which are crucial in the ischemic pathology of PAD. Pathway enrichment analysis highlighted the central position of the HIF-1 signaling pathway within XFZYC’s action network, providing a modern scientific interpretation for its traditional efficacy of “promoting blood circulation and removing blood stasis.” Molecular docking results provided strong support for this: while multiple active components in XFZYC exhibited binding affinity with both PFKFB3 and HIF-1α, the binding energies for PFKFB3 were consistently stronger (generally < −6.5 kcal/mol), whereas those for HIF-1α were relatively weaker. Therefore, this study prioritizes PFKFB3 as a potential key node through which XFZYC may regulate glycolysis. In summary, we hypothesize that XFZYC acts by modulating glycolytic metabolism in endothelial cells, with a primary focus on regulating the critical target PFKFB3, thereby alleviating ischemia-related symptoms in PAD.

To validate the predictions from network pharmacology and molecular docking, this study first evaluated the effects of different concentrations (1.25%, 2.5%, and 5%) of XFZYC-containing serum on HMEC-1 cells. The results indicated that the 2.5% drug-containing serum demonstrated the most significant effects in enhancing tube-forming capacity and upregulating the protein expression of HK2, PFKFB3, and HIF-1α. Notably, a non-monotonic dose–response relationship was observed: the 5% drug-containing serum did not exhibit a stronger promotive effect but instead showed inhibitory or non-significant effects on angiogenesis-related indicators. This phenomenon is consistent with previous findings, which also reported that 2.5% XFZYC-containing serum yielded the best outcomes in promoting vascular endothelial cell function, whereas the 5% concentration showed negligible effects [30]. We speculate that such a typical “U-shaped” or “bell-shaped” dose–response curve is not uncommon in studies involving complex traditional medicine systems. The underlying reason may be that certain active components at higher concentrations lead to overactivation and even inhibition of specific signaling pathways. Based on these empirical findings and mechanistic considerations, 2.5% XFZYC-containing serum was selected for subsequent experiments.

Previous research on the pro-angiogenic effects of XFZYD has primarily focused on the regulation of angiogenesis-related genes. For instance, XFZYC-containing serum was found to transiently promote angiogenesis in human microvascular endothelial cells by downregulating Kruppel-like factor 2 (KLF2) protein expression [31]. XFZYC may regulate angiogenesis by modulating the expression of Jagged1 and Dll4 within the Notch signaling pathway [23,24]. XFZYD promoted the expression of CD106 and CD146 in endothelial progenitor cells and increased serum and myocardial nitric oxide (NO) levels, thereby fostering angiogenesis in the myocardial ischemic area [32,33]. The present study discovered that while promoting in vitro tube formation in HMEC-1 cells, XFZYC significantly enhanced the activity of key glycolytic enzymes and PFKFB3 protein expression. Crucially, this effect was reversible by the specific inhibitor 3PO, unveiling the modern biological basis—regulation of cellular metabolic reprogramming—underlying the action of this traditional formula.

Glycolysis, a core energy metabolism pathway in endothelial cells, orchestrates the entire process of angiogenesis—initiation, extension, and maturation—by regulating ATP production and metabolic substrate supply. HK, PFK, and PK are three key glycolytic enzymes [34,35]. Among them, the reaction catalyzed by PFK constitutes the rate-limiting step of glycolysis. Fructose-2,6-bisphosphate (F-2,6-BP), its most potent activator, is critical for controlling the glycolytic rate. PFKFB3 catalyzes the synthesis of F-2,6-BP, thereby activating glycolysis and serving as a powerful controller enhancing glycolytic flux [36]. It is this unique position at the gateway of the pathway’s committed step that makes PFKFB3 a strategic and highly sensitive intervention point for modulating endothelial glycolysis [37]. Distinct from most aerobic cells, endothelial cells rely predominantly on glycolysis, which not only enables faster energy generation and more efficient production of pro-angiogenic metabolites like lactate but also allows more O_2_ to be supplied to neighboring cells, minimizing ROS generation and associated damage [7]. Network pharmacology and molecular docking predictions identified HK2 and PFKFB3 as potential glycolytic targets of XFZYC. PFKFB3 was prioritized as the leading candidate for further experimental validation based on two compelling reasons: first, it achieved the most favorable molecular docking scores, indicating a high probability of direct compound binding; second, and more importantly, its upstream regulatory role over the pathway’s key flux-controlling enzyme PFK positions it as a potent leverage point for effectively reprogramming global glycolytic output. Subsequent experiments confirmed that the prescription promotes the expression of both HK2 and PFKFB3. However, this study has certain limitations: it has not been verified whether specific components within the formula directly target PFKFB3, nor has the involvement of the HIF-1α pathway been confirmed. Therefore, the present results only suggest that the observed drug effects may be attributable to action on PFKFB3, rather than mediated through a specific signaling pathway.

In recent years, the development of therapeutic angiogenesis strategies based on Vascular Endothelial Growth Factor (VEGF) has faced obstacles due to limited efficacy, side effects, drug resistance, and other complex, difficult-to-resolve issues [38]. Consequently, strategies targeting endothelial cell metabolism have emerged as promising alternatives, particularly for treating cardiovascular diseases, cancer, and ischemic conditions [39]. Among these, the PFKFB3 inhibitor PFK158 has shown significant effects in anti-tumor and anti-angiogenesis applications and has entered Phase I clinical trials (ClinicalTrials.gov: NCT02044861) [7].

Basic research indicates that glycolytic metabolism has a significant protective effect in mouse models of hindlimb ischemia [40]. Upregulation of key glycolytic enzyme activity can significantly promote endothelial cell proliferation, migration, and tube formation. Conversely, downregulating glycolysis inhibits the activation of Vascular Endothelial Growth Factor Receptor 2 (VEGFR2), leading to reduced levels of ERK and AKT, and consequently suppressing endothelial cell proliferation, migration, and in vitro tube formation [40]. Furthermore, key glycolytic enzymes such as HK, PKM2, and PFKFB3 have all been demonstrated to be involved in angiogenesis [41]. Specifically, HK2 enhances the transcriptional activity of HIF-1α by increasing the accumulation of glycolytic intermediates, thereby promoting the expression of pro-angiogenic factors like VEGF [26,42]. Nuclear translocation of PKM2 drives the expression of angiogenic genes via the HIF-1α/VEGF signaling axis [43]. PFKFB3, by enhancing glycolysis, supports endothelial cell proliferation, migration, and angiogenesis. Knockdown of PFKFB3 inhibits the activity of tip cells and stalk cells, affecting vascular sprouting [44].

Additionally, key metabolites of glycolysis have been proven to exert various influences on EC angiogenesis. For instance, supplementation with the intermediate fructose-6-phosphate helps maintain normal VEGFR2 and Notch functionality. The end product lactate stabilizes HIF-1α, activates the VEGF signaling pathway and VEGFR2 expression, promotes angiogenesis, and aids in revascularization and damaged tissue regeneration post-ischemia [45]. This study demonstrates that XFZYC, while promoting glucose uptake and lactate production, inhibits citrate synthesis, suggesting it may influence cellular metabolism by regulating the distribution of metabolic intermediates. This aligns highly with previously identified functions of pro-angiogenic metabolites like fructose-6-phosphate and lactate, indicating that XFZYC might amplify its biological effects through these glycolytic metabolites. Moreover, PFKFB3, as a downstream target of Notch and KLF2 signaling, might, upon its upregulated expression, disrupt the metabolic balance between tip and stalk cells. This possesses internal logical consistency with our team’s previous findings that XFZYC regulates the expression of Dll4/Jagged1 and KLF2 [23,24,31]. Furthermore, the increased ATP supply resulting from enhanced glycolysis might promote protein synthesis by activating mTORC1 signaling, while lactate accumulation could indirectly regulate VEGF expression by stabilizing HIF-1α. These multi-layered regulatory networks collectively constitute the molecular basis for the formula’s characteristic “multi-target, multi-pathway” mode of action.

This study provides the first evidence demonstrating a direct link between XFZYC and the promotion of angiogenesis through its association with glycolytic metabolism, offering preliminary in vitro support for this mechanism. However, angiogenesis involves a complex network of glucose metabolic regulation, and XFZYC itself contains multiple components with broad potential targets. Therefore, its precise pro-angiogenic mechanism remains to be fully elucidated. The current research has several limitations. First, network pharmacology and molecular docking predictions identified a large number of potential targets (2967), among which components showing potential binding affinity for PFKFB3 were mostly derived from Gancao. Given that licorice primarily serves as a harmonizing and guiding agent in the formula, these predictions may include false positives. This highlights the need for subsequent specialized validation focusing on high-scoring docking components such as kaempferol. Second, as a key regulatory node in the glycolytic pathway, the precise mechanism of PFKFB3 remains unclear. Further experiments are needed to determine whether active components in XFZYC (e.g., kaempferol) directly bind to and modulate PFKFB3 function. Additionally, the present study only used 3PO as an inhibitor to investigate the role of glycolysis in the pro-angiogenic effect of XFZYC. Although 3PO is generally recognized as a glycolysis inhibitor, the possibility of its off-target effects cannot be completely ruled out. Therefore, techniques such as gene knockout or silencing could be employed to further verify the central role of PFKFB3 in the XFZYC-mediated regulatory pathway. Furthermore, how glycolytic metabolism interacts with classical angiogenic signaling pathways such as VEGFR2 represents an important scientific question that requires clarification. Finally, the current findings are still confined to the cellular level. Future studies should employ animal models such as hindlimb ischemia to further validate the functional relationship between XFZYC and the glycolytic pathway in vivo.

Based on these limitations, future research will focus on isolating and identifying one or two high-potential active components from XFZYC (e.g., kaempferol) and separately evaluating their effects on endothelial cell glycolysis and angiogenic function. This will help clarify the specific material basis driving the observed pharmacological effects and deepen the understanding of XFZYC’s mechanism of action.

In summary, our in vitro findings demonstrate that XFZYC enhances pro-angiogenic functions—including proliferation, migration, adhesion, and tube formation—in HMEC-1 cells by upregulating PFKFB3 and promoting glycolytic metabolic reprogramming. Inhibition of glycolysis effectively attenuates these XFZYC-induced phenotypes. This study is the first to link the pharmacological activity of XFZYC with endothelial metabolic reprogramming, thereby revealing a previously unexplored dimension of its mechanism. While these results provide novel mechanistic insight into how this blood-activating and stasis-resolving formula may promote angiogenesis, further in vivo validation remains essential. Collectively, our work contributes to the growing framework for developing therapeutic angiogenesis strategies based on metabolic regulation and supports the continued investigation of traditional herbal medicines in this context.

## 4. Materials and Methods

### 4.1. Animals and Cells

Fifty male Sprague-Dawley (SD) rats, 8-week-old and SPF grade, with an average body weight of 300 ± 10 g, were used [Laboratory Animal Production License: SCXK (Zhe) 2019-02]. They were housed in the SPF laboratory animal facility of the Fujian University of Traditional Chinese Medicine [Laboratory Animal Use License: SYXK (Min) 2019-0007]. The animal study protocol was approved by the Animal Ethics Committee of Fujian University of Traditional Chinese Medicine (Approval No. 2021046). HMEC-1 were purchased from Xiamen Immocell Biotechnology Co., Ltd, Xiamen, China. (Product No. IM-H194).

### 4.2. Medicine

Xuefu Zhuyu Capsules (XFZYC) were manufactured by Tianjin Hongrentang Pharmaceutical Co., Ltd, Tianjin, China. (National Medicine Permit No.: Z12020223; Batch No.: BA03310; Specification: 0.4 g/capsule).

### 4.3. Reagents

MCDB-131 medium, pentobarbital sodium, and dimethyl sulfoxide (DMSO) were purchased from Sigma-Aldrich, St. Louis, MO, USA (Product Nos.: M8537, 1030001, D2650, respectively). 3PO was purchased from MedChemExpress, Monmouth Junction, NJ, USA (Product No.: HY-19824-1MG). Fetal Bovine Serum (FBS) was purchased from Thermo Fisher Scientific,Waltham, MA, USA (Product No.: A5256701). The Glucose Assay Kit was produced by Nanjing Jiancheng Bioengineering Institute, Nanjing, China (Product No.: F006-1-1). Assay kits for Hexokinase (HK) activity, Phosphofructokinase (PFK) activity, Pyruvate Kinase (PK) activity, L-Lactic Acid (LA) content, and Citric Acid (CA) content were all from Wuhan elabscience Bio-Technology Co.Ltd. Wuhan, China.(Product Nos.: E-BC-K610-M, E-BC-K612-M, E-BC-K611-M, E-BC-K044-M, E-BC-K351-M, respectively). Trizol was purchased from Invitrogen, Carlsbad, CA, USA (Product No.: 101105). RIPA Lysis Buffer, BCA Protein Assay Kit, and Horseradish Peroxidase (HRP)-conjugated Goat Anti-Rabbit Immunoglobulin G (IgG) were supplied by Beyotime Biotechnology, Shanghai, China (Product Nos.: P0013B, P0010S, A0208, respectively). Rabbit anti-β-actin and rabbit anti-PFKFB3 antibodies were purchased from Cell Signaling Technology, Danvers, MA, USA (Product Nos.: 4970S, 13123S, respectively). The CCK-8 assay kit was provided by Shanghai LiJi Biological Technology Co., Ltd, Shanghai, China (Product No.: AC11L054). The in vitro Angiogenesis Assay Kit was purchased from Millipore, Burlington, MA, USA (Product No.: 2658909). The ECL chemiluminescence detection kit was purchased from Beijing Cowin Biotech Co., Ltd, Beijing, China(Product No.: 20130).

### 4.4. Screening of Active Drug Components and Action Targets

The TCMSP database (https://www.tcmsp-e.com/, accessed on 25 July 2025) was used to retrieve the active components of Xuefu Zhuyu Decoction (comprising Chaihu, Chishao, Chuanxiong, Danggui, Gancao, Honghua, Jiegeng, Niuxi, Shengdihuang, Taoren, and Zhiqiao) [46]. Screening was performed using the criteria of Oral Bioavailability (OB) ≥ 30% and Drug Likeness (DL) ≥ 0.18. For herbal components not found in the TCMSP database, the HERB database (http://herb.ac.cn, accessed on 25 July 2025) was utilized, and active components were screened based on Lipinski’s Rule of Five [47]. The specific steps were as follows: active components of “Shengdihuang” were downloaded, intersected with all active components in the HERB database to obtain detailed data (molecular weight and PubChem ID). Using the PubChem ID, compound details were downloaded from the PubChem website to acquire the canonical SMILES. The canonical SMILES of the screened compounds were then subjected to further screening using the SwissADME platform (http://www.swissadme.ch/, accessed on 25 July 2025). Compounds whose canonical SMILES were unavailable were converted using the 3D molecule to SMILES tool on the NovoPro platform (https://www.novopro.cn/, accessed on 25 July 2025). Final screening criteria were a Gastrointestinal (GI) absorption score of “High” and the Druglikeness property meeting at least two “Yes” criteria. These were included as supplementary candidate active components.

### 4.5. Acquisition of Peripheral Artery Disease-Related Targets

The peripheral artery disease dataset (GSE120642) was obtained from the GEO database (https://www.ncbi.nlm.nih.gov/geo/, accessed on 25 July 2025) [25]. The R package (version 4.5.1) limma was used for background correction and normalization of the dataset to obtain an expression matrix. Differentially expressed genes (DEGs) were screened using the thresholds *p* < 0.05, FDR < 0.05 and |log2(Fold Change)| ≥ 1. Visualization was performed by generating volcano plots and heatmaps.

### 4.6. Enrichment Analysis

To explore the biological functions and pathways associated with the key genes, Gene Ontology (GO) and Kyoto Encyclopedia of Genes and Genomes (KEGG) enrichment analyses were performed using the clusterProfiler package in R. Gene symbols were converted to Entrez IDs using the org.Hs.eg.db R package. GO analysis covered three categories: Biological Process (BP), Cellular Component (CC), and Molecular Function (MF). The significance threshold was set at *p* < 0.05 and q < 0.1. The top 10 most significantly enriched terms were selected for presentation. KEGG pathway enrichment analysis was performed using the same threshold criteria. Enrichment results were visualized using circle-based plots [48].

### 4.7. Molecular Docking Validation

The core target proteins PFKFB3 (PDB ID: 2DWO), HIF-1α (PDB ID: 8HE0), and HK2 (PDB ID: 2NZT) were selected for molecular docking. Protein structures were retrieved from the RCSB Protein Data Bank (https://www.rcsb.org/, accessed on 25 July 2025). Structures were filtered based on resolution (<2.5 Å), experimental method (X-ray crystallography), species (Homo sapiens), chain length, and the presence of bound ligands. Downloaded crystal structures were processed using PyMOL software (version 3.1.3) to remove water molecules and small ligands. Receptors were prepared in AutoDockTools (version 1.5.7) by adding non-polar hydrogen atoms, calculating charges, ensuring a rigid receptor conformation, and saving as PDBQT files. Small molecule ligands selected from the previously screened active compounds were downloaded in MOL2 format from the TCMSP database or in SDF format from PubChem. OpenBabel software (version 3.1.1) was used to convert all ligands to the appropriate format. Ligand structures were further refined in PyMOL by removing water molecules and co-crystallized small ligands. AutoGrid was used to define the grid box parameters for molecular docking. The grid box dimensions were set to sufficiently encompass the entire binding site of the receptor. Grid spacing was adjusted within the allowable range of 0.2–1.0 Å to ensure comprehensive coverage of the binding pocket and to accommodate all reasonable ligand conformations during docking. Molecular docking was performed in AutoDock to calculate binding energies, and PyMOL was used to visualize the results and illustrate molecular interactions [49,50].

### 4.8. Preparation of Drug-Containing Serum and Blank Serum

SD rats were acclimatized for 7 days and then randomly divided into a Drug Group and a Blank Group using a random number method, with 25 rats per group. Xuefu Zhuyu Capsules were dissolved in normal saline. Referring to the dose conversion method between humans and animals, the daily administration dose for the Drug Group was 12 times the normal human dose per unit body weight, i.e., 0.96 g·kg^−1^·d^−1^. The Blank Group received an equal volume of normal saline. Both groups were administered via gavage twice daily for 7 consecutive days. Two hours after the last administration, rats were anesthetized using 3% pentobarbital sodium. Blood was collected from the abdominal aorta and centrifuged at 4000 r·min^−1^ for 30 min to separate the serum. The obtained serum was inactivated at 56 °C for 30 min to deactivate complement, filtered through a 0.22 μm membrane for sterilization, and stored at −20 °C for later use.

### 4.9. Cell Culture

HMEC-1 cells were maintained in MCDB-131 medium supplemented with 10% fetal bovine serum at 37 °C under 5% CO_2_. The cells were seeded in 6-well plates at a density of 2.5 × 10^5^ cells/mL, with 1 mL per well. Following synchronization, the optimal concentration of XFZYC medicated serum was determined by testing three concentrations (1.25%, 2.5%, and 5%), each with corresponding blank serum control groups and XFZYC medicated serum treatment groups, resulting in a total of six experimental conditions. Based on these results, the optimal concentration was identified as 2.5%. Subsequently, the cells were randomly divided into three groups for further experiments: a blank serum group (2.5% blank serum), an XFZYC group (2.5% XFZYC medicated serum), and an XFZYC + 3PO group. In the XFZYC + 3PO group, cells were pretreated with 20 μmol·L^−1^ 3PO for 2 h, as established in the previous literature and preliminary experiments, after which the medium was replaced with one containing 2.5% XFZYC medicated serum for an additional 24 h of intervention.

### 4.10. Cell Behavior Analysis

#### 4.10.1. In Vitro Tube Formation Assay

After 24 h of culture under different treatments, cells from each group were collected. The In Vitro Angiogenesis Assay was performed according to the kit instructions. Briefly, 50 μL of pre-mixed ECMatrix™ solution was evenly spread into pre-chilled (4 °C) 96-well plates and allowed to solidify at 37 °C with 5% CO_2_ for 2 h. Then, 3 × 10^4^ cells in 150 μL medium were seeded per well. After 6 h of incubation at 37 °C, six random high-power fields (×200 magnification) were selected, and the total number of formed tubes was counted

#### 4.10.2. CCK-8 Assay

HMEC-1 cells in good growth condition were seeded in 96-well plates at 1 × 10^5^ cells per well and cultured until ~50% confluent. Cells were then treated according to the groups described in Section 4.9, with 6 replicate wells per group, for 24 h. The culture medium was discarded, and 100 μL of MCDB-131 medium containing 10% CCK-8 reagent was added to each well. Cells were incubated in the cell culture incubator for 2 h, and the absorbance (A value) of each well was measured at a wavelength of 450 nm. The cell proliferation rate was calculated as: Cell Proliferation Rate (%) = [(Aexperimental − Ablank)/(Acontrol − Ablank)] × 100%.

#### 4.10.3. Scratch Wound Healing Assay

Cells from each group were collected and seeded into 6-well plates at 5 × 10^5^ cells per well, and cultured overnight at 37 °C with 5% CO_2_. Once cells reached 100% confluence, two parallel scratch wounds were made per well using a pipette tip. After grouping and treatment as described in Section 4.9, cells were washed with PBS, and the medium was replaced with serum-free medium. Cells were cultured for 12 h, then observed and photographed. Cell migration was analyzed using ImageJ software (version 1.8.0). Migration Rate (%) = [(Initial scratch width-Scratch width at specified time point)/Initial scratch width] × 100%.

#### 4.10.4. Transwell Assay

Cells in the logarithmic growth phase were seeded in 6-well plates at a density of 2 × 10^5^ cells per well and intervened according to the groups described in Section 4.9 for 24 h at 37 °C with 5% CO_2_. Cells were then collected, counted, and 2 × 10^3^ cells in serum-free medium were added to the upper chamber of a Transwell insert. After 12 h of incubation, cells that migrated to the lower side of the membrane were fixed for 15 min, stained with crystal violet for 20 min according to the kit instructions, air-dried, photographed, and the number of migrated cells was counted. Each group had 3 replicate chambers.

#### 4.10.5. Cell Adhesion Assay

After 24 h of culture under different treatments, cells from each group were seeded in 96-well plates at 2 × 10^3^ cells per well and cultured for 1 h at 37 °C with 5% CO_2_. The culture medium was then discarded. Cells were fixed for 15 min and stained with crystal violet for 20 min according to the kit instructions, air-dried, photographed, and the number of adherent cells was counted. Each group had 6 replicate wells.

### 4.11. Colorimetric Assay for Glycolysis-Related Metabolite Levels and Key Enzyme Activities

Cells from each group were collected, lysed by ultrasonic disruption, and centrifuged at 12,000 r·min^−1^ for 10 min to collect the supernatant. According to the BCA Protein Assay Kit instructions, the BCA working solution was prepared. Protein standards and samples were added to a 96-well plate, followed by the BCA working solution. The plate was then read using a microplate reader to calculate the protein concentration of the samples. According to the respective assay kit instructions, cells were processed to analyze glucose consumption, lactate production, and citrate content, and to measure the activities of Hexokinase (HK), Phosphofructokinase (PFK), and Pyruvate Kinase (PK).

### 4.12. Western Blot Analysis of PFKFB3, HK2, and HIF-1α Protein Levels

After the respective treatments, the culture medium was discarded, and cells were washed three times with ice-cold PBS. Cells were lysed on ice for 30 min using RIPA lysis buffer containing PMSF, followed by centrifugation at 4 °C, 12,000 r·min^−1^ for 10 min. The supernatant was collected, and the protein concentration was determined using the BCA method. Proteins were separated by SDS-PAGE and transferred onto PVDF membranes. Membranes were blocked in rapid blocking buffer at room temperature for 30 min. Subsequently, membranes were incubated overnight at 4 °C with shaking with primary antibodies against PFKFB3 (1:2000), HK2 (1:1000), HIF-1α (1:1000), and β-actin (1:1000). After incubation, membranes were washed 6 times with TBST, 5 min each, followed by incubation with HRP-conjugated secondary antibody (1:2000) for 1 h. After washing, bands were visualized using ECL chemiluminescence. Band intensity (gray value) was analyzed using ImageJ software.

### 4.13. Statistical Analysis

All statistical analyses were performed using SPSS software (version 25.0). Continuous data that followed a normal distribution are presented as mean ± standard deviation (x ± s). Specific comparisons between drug-containing serum and blank serum at each concentration were assessed by two-tailed Student’s t-test, while multiple group comparisons were conducted using one-way analysis of variance (ANOVA). A *p*-value of less than 0.05 was considered statistically significant.

## 5. Conclusions

This study demonstrates that Xuefu Zhuyu Capsule promotes angiogenesis by upregulating PFKFB3 expression, enhancing glycolytic metabolism in endothelial cells, and thereby facilitating cell proliferation, migration, adhesion, and in vitro tube formation. These findings not only reveal a novel mechanism underlying the traditional Chinese medicine efficacy of “promoting blood circulation and removing blood stasis” from the perspective of energy metabolism but also provide a scientific basis for therapeutic angiogenesis strategies targeting metabolic regulation.

## Figures and Tables

**Table 1:** Summary of Active Components and Their Targets in Xuefu Zhuyu Capsules.

Herb Name	Number of Active Components	Number of Predicted Targets
Chaihu	13	374
Chishao	15	195
Chuanxiong	4	253
Danggui	2	44
Gancao	86	775
Honghua	11	343
Jiegeng	2	101
Niuxi	8	408
Shengdihuang ^1^	3	55
Taoren	19	251
Zhiqiao	4	168
Total	167	2967

^1^ Data sourced from the HERB database.

**Table 2:** Bioactive compounds with docking results against PFKFB3 greater than −6.5 kcal·mol^−1^.

Mol ID	Name	Herbal Sources	Binding Energy (kcal∙mol^−1^)
MOL000422	Kaempferol	Caihu, Honghua, Niuxi, Gancao	−6.95
MOL000239	Jaranol	Gancao	−6.99
MOL003656	Lupiwighteone	Gancao	−6.68
MOL003896	7-Methoxy-2-methyl isoflavone	Gancao	−6.68
MOL004806	Euchrenone	Gancao	−6.76
MOL004856	Gancaonin A	Gancao	−7.15
MOL004891	Shinpterocarpin	Gancao	−7.22
MOL004991	7-Acetoxy-2-methylisoflavone	Gancao	−7.36

## Data Availability

Data are contained within the article and Supplementary Materials.

## Supplementary Materials

The following supporting information can be downloaded at: https://www.mdpi.com/article/10.3390/ph18121902/s1, Table S1: Summary of Drugs, Active Components and Their Targets.

## Abbreviations

The following abbreviations are used in this manuscript:

3PO	3-(3-pyridinyl)-1-(4-pyridinyl)-2-propen-1-one (a glycolytic inhibitor)
ATP	Adenosine Triphosphate
BCA	Bicinchoninic Acid
BP	Biological Process
CC	Cellular Component
CCK-8	Cell Counting Kit-8
DEG	Differentially Expressed Gene
DMSO	Dimethyl Sulfoxide
EC	Endothelial Cell
ECL	Enhanced Chemiluminescence
FBS	Fetal Bovine Serum
FDR	False Discovery Rate
GI	Gastrointestinal
GO	Gene Ontology
HEMC-1	Human Microvascular Endothelial Cell line-1
HERB	A high-throughput experiment- and reference-guided database of traditional Chinese medicine
HIF-1α	Hypoxia-Inducible Factor 1-alpha
HK	Hexokinase
HK2	Hexokinase 2
HRP	Horseradish Peroxidase
IgG	Immunoglobulin G
KEGG	Kyoto Encyclopedia of Genes and Genomes
KLF2	Krüppel-like Factor 2
LA	Lactic Acid
MF	Molecular Function
mTOR	Mechanistic Target of Rapamycin
mTORC1	Mechanistic Target of Rapamycin Complex 1
NO	Nitric Oxide
OB	Oral Bioavailability
PAD	Peripheral Artery Disease
PBS	Phosphate-Buffered Saline
PC	Pyruvate Carboxylase
PFK	Phosphofructokinase
PFKFB3	6-Phosphofructo-2-kinase/Fructose-2,6-biphosphatase 3
PK	Pyruvate Kinase
PKM2	Pyruvate Kinase Isoenzyme M2
PPI	Protein–Protein Interaction
PVDF	Polyvinylidene Difluoride
RIPA	Radioimmunoprecipitation Assay
ROS	Reactive Oxygen Species
SDS-PAGE	Sodium Dodecyl Sulfate Polyacrylamide Gel Electrophoresis
SMILES	Simplified Molecular Input Line Entry System
SPF	Specific Pathogen-Free
TBST	Tris-Buffered Saline with Tween
TCA	Tricarboxylic Acid
TCMSP	Traditional Chinese Medicine Systems Pharmacology
VEGF	Vascular Endothelial Growth Factor
VEGFR2	Vascular Endothelial Growth Factor Receptor 2
XFZYC	Xuefu Zhuyu Capsule

## Appendix A

**Table A1:** Summary of Molecular Docking Results.

Mol ID/Name	Binding Protein	Binding Energy (kcal∙mol^−1^)
MOL000422	PFKFB3	−6.95
MOL004598	PFKFB3	−5.63
MOL000239	PFKFB3	−6.99
MOL000392	PFKFB3	−5.98
MOL000417	PFKFB3	−5.84
MOL000422	PFKFB3	−6.68
MOL003656	PFKFB3	−6.68
MOL003896	PFKFB3	−6.76
MOL004806	PFKFB3	−6.14
MOL004814	PFKFB3	−5.45
MOL004828	PFKFB3	−5.72
MOL004835	PFKFB3	−7.15
MOL004856	PFKFB3	−6.38
MOL004857	PFKFB3	−6.29
MOL004863	PFKFB3	−5.72
MOL004866	PFKFB3	−7.22
MOL004891	PFKFB3	−6.34
MOL004908	PFKFB3	−5.8
MOL004915	PFKFB3	−6.2
MOL004949	PFKFB3	−6.29
MOL004957	PFKFB3	−5.66
MOL004990	PFKFB3	−7.36
MOL004991	PFKFB3	−5.9
MOL005007	PFKFB3	−5.96
MOL005016	PFKFB3	−5.49
MOL000006	PFKFB3	−4.73
MOL002695	PFKFB3	−5.29
MOL002712	PFKFB3	−5.96
MOL002714	PFKFB3	−5.55
MOL002717	PFKFB3	−3.49
MOL002151	PFKFB3	−5.14
MOL000173	PFKFB3	−4.1
MOL001002	PFKFB3	−4.39
MOL000785	PFKFB3	−1.98
MOL004702	HIF-1α	−5.03
MOL004820	HIF-1α	−5.39
MOL006992	HIF-1α	−3.35
MOL005008	HIF-1α	−5.5
MOL004824	HIF-1α	−2.17
Rehmaglutin C	HK2	−6.95
