# Supplementary material for: Investigating the Role of Glycolysis in Xuefu Zhuyu Capsule-Promoted Angiogenesis in Endothelial Cells: A Study Based on Network Pharmacology, Molecular Docking, and In Vitro Validation
Source: Pharmaceuticals (Basel). 2025 Dec 17;18(12):1902. doi: 10.3390/ph18121902 (PMC12735633; doi:10.3390/ph18121902)
Supplement: Supplementary file 1 [file pharmaceuticals-18-01902-s001.zip › pharmaceuticals-3995543-supplementary.pdf]

Table S1. Summary of Drugs, Active Components and Their Targets

Drug	Active-ingredient Mol ID	Target
Chai Hu	MOL000098	ABCG2
		AKR1B1
		NOX4
		PLG
		ADORA3
		XDH
		CA2
		CA7
		CA12
		CA4
		CYP1B1
		APP
		ADORA1
		ABCB1
		ADORA2A
		MAPT
		KDM4E
		GPR35
		AVPR2
		TOP2A
		MAOA
		IGF1R
		FLT3
		CYP19A1
		INSR
		EGFR
		F2
		PIM1
		ALOX5
		AURKB
		DRD4
		ACHE
		GLO1
		MYLK
		MPO
		PIK3R1
		DAPK1
		PYGL
		SYK
		CA1

GSK3B
SRC
PTK2
HSD17B2
KDR
MMP13
MMP3
CA3
ALOX15
ABCC1
PLK1
CA6
CDK1
MMP9
PIK3CG
MMP2
PKN1
CA14
CA9
CSNK2A1
ALOX12
MET
NEK2
CXCR1
CAMK2B
ALK
AKT1
NEK6
PLA2G1B
CA5A
AXL
APEX1
NUAK1
AKR1C2
AKR1C1
AKR1C3
AKR1C4
CA13
AKR1A1
PTGS2
PLA2G2A
PTPRS
ESR2
MPG

	SLC22A12
	OPRM1
	ODC1
	CDK5R1
	CCNB3
	CDK6
	CDK2
	ARG1
	TERT
	NOS2
	TYR
	HSD17B1
	AHR
	ESRRA
	MCL1
	BCHE
MOL000354	XDH
	CA2
	CA7
	CA12
	CA4
	CYP1B1
	ABCC1
	NOX4
	AKR1B1
	ABCG2
	ACHE
	ALOX15
	ALOX12
	IGF1R
	EGFR
	AVPR2
	MAOA
	FLT3
	CYP19A1
	F2
	PIM1
	ALOX5
	AURKB
	DRD4
	ADORA1
	GLO1
	MPO
	PIK3R1

ADORA2A
DAPK1
PYGL
CA1
GSK3B
SRC
PTK2
HSD17B2
KDR
MMP13
MMP3
CA3
PLK1
CA6
CDK1
MMP9
MMP2
PKN1
CA14
CA9
CSNK2A1
MET
NEK2
CXCR1
CAMK2B
ALK
AKT1
ABCB1
NEK6
PLA2G1B
CA5A
BACE1
AXL
NUAK1
AKR1C2
AKR1C1
AKR1C3
AKR1C4
CA13
AKR1A1
GPR35
MAPT
KDM4E
TOP2A

	INSR
	MYLK
	SYK
	PIK3CG
	APEX1
	CDK5
	CCNB3
	CDK6
	CDK2
	ARG1
	APP
	MCL1
	TERT
	TYR
	HSD17B1
	AHR
	ESRRA
	PTPRS
	PLG
	ESR2
	MPG
	SLC22A12
	PARP1
	TTR
	MMP12
	CD38
	AKR1B10
	TNKS2
MOL000422	NOX4
	AKR1B1
	XDH
	TYR
	FLT3
	CA2
	ALOX5
	CA7
	HSD17B2
	ABCC1
	HSD17B1
	AHR
	CA12
	ESRRA
	ABCB1
	CYP1B1

ABCG2

ADORA1

CA4

ACHE

MAOA

GLO1

SYK

GSK3B

MMP9

MMP2

ALOX15

ALOX12

PTPRS

ADORA2A

CDK5

CCNB3

ARG1

GPR35

ESR2

DAPK1

MPG

SLC22A12

TTR

AKR1B10

TNKS2

TNKS

CDK6

CDK2

CYP19A1

CSNK2A1

EGFR

AVPR2

IGF1R

F2

PIM1

AURKB

DRD4

MPO

PIK3R1

PYGL

CA1

SRC

PTK2

KDR

	MMP13
	MMP3
	CA3
	PLK1
	CA6
	CDK1
	PKN1
	CA14
	CA9
	MET
	NEK2
	CXCR1
	CAMK2B
	ALK
	AKT1
	NEK6
	PLA2G1B
	CA5A
	BACE1
	AXL
	NUAK1
	AKR1C2
	AKR1C1
	AKR1C3
	AKR1C4
	CA13
	AKR1A1
	APP
	PARP1
	MMP12
	CD38
	TOP1
	ESR1
	PTGS2
	CFTR
	PFKFB3
	AMY1A
	GRK6
	TERT
	MAPT
MOL000490	GLO1
	F2
	NR1H3
	XDH

CA2
CA7
CA12
CA4
CYP1B1
AKR1B1
ABCG2
NOX4
CD38
ADORA3
ABCB1
MAPT
KDM4E
GPR35
AVPR2
TOP2A
MAOA
IGF1R
FLT3
CYP19A1
INSR
EGFR
PIM1
ALOX5
AURKB
DRD4
ACHE
ADORA1
MYLK
MPO
PIK3R1
DAPK1
PYGL
SYK
CA1
GSK3B
SRC
PTK2
HSD17B2
KDR
MMP13
MMP3
CA3
ALOX15

ABCC1
PLK1
CA6
CDK1
MMP9
PIK3CG
MMP2
PKN1
CA14
CA9
CSNK2A1
ALOX12
MET
NEK2
CXCR1
CAMK2B
ALK
AKT1
NEK6
PLA2G1B
CA5A
BACE1
AXL
APEX1
NUAK1
AKR1C2
AKR1C1
AKR1C3
AKR1C4
CA13
ADORA2A
AKR1A1
ESR1
PLG
CDK5
CCNB3
CDK6
CDK2
ARG1
APP
OPRD1
TERT
ESR2
ST6GAL1

	PLA2G2A
	TYR
	HSD17B1
	AHR
	ESRRA
	PTGS1
	PDE4B
	PDE4D
MOL004598	ABCG2
	PLG
	ADORA3
	ADORA1
	ADORA2A
	CA2
	CA7
	CA12
	PLK1
	ABCB1
	AKR1B1
	NOX4
	FLT3
	EGFR
	KDR
	XDH
	AURKB
	SYK
	GSK3B
	CYP1B1
	CA1
	CA13
	ALOX5
	PIM1
	MET
	BACE1
	SRC
	CDK1
	PTK2
	APP
	PIK3CG
	CA6
	CSNK2A1
	AXL
	CDK5
	KIT

F2
MMP13
IGF1R
INSR
MYLK
DAPK1
PKN1
NEK2
ALK
NEK6
NUAK1
MCL1
MMP2
KDM4E
TOP2A
PIK3R1
PYGL
CA3
PLA2G1B
CA5A
APEX1
AKR1C2
AKR1C1
AKR1C4
AKR1A1
CAMK2B
OPRM1
DRD4
MMP3
MMP9
CDK2
MAOA
OPRD1
CA9
CA4
AKT1
NOS2
ACHE
AVPR2
CDK6
ARG1
ALOX15
CYP19A1
PTGS2

	ALOX12
	CCNB3
	TYR
	AHR
	HSD17B2
	ESR2
	ESRRA
	ABCC1
	CA14
	MAPT
	PARP1
	AKR1C3
	ST6GAL1
	MPO
	PTPRS
	MPG
	GPR35
	PFKFB3
	TERT
	CSF1R
MOL004609	AKR1B1
	ABCG2
	OPRD1
	ADORA1
	ALOX5
	ADORA2A
	ADORA3
	CA2
	CA7
	CA4
	NOX4
	XDH
	CYP1B1
	ESR2
	PLG
	PLA2G2A
	ABCC1
	CA12
	MAPT
	KDM4E
	GPR35
	TOP2A
	GLO1
	MPO

PIK3R1
DAPK1
PYGL
CA3
PKN1
CSNK2A1
NEK2
CAMK2B
NEK6
PLA2G1B
APEX1
NUAK1
AKR1C2
AKR1C1
AKR1C3
AKR1C4
AKR1A1
PTPRS
BACE1
CD38
TNKS2
TNKS
ARG1
KIT
MMP13
MMP2
MPG
SLC22A12
PIM1
IGF1R
INSR
EGFR
SRC
PTK2
KDR
OPRM1
CDK6
MYLK
GSK3B
HSD17B2
AMY1A
ODC1
FLT3
TYR

	AHR
	ESRRA
	APP
	MMP3
	TERT
	ALOX12
	MAOA
	CA14
	CA9
	CA5A
	CDK2
	MET
	ABCB1
	ALOX15
	MMP9
	AURKB
	CFTR
	NAE1
	CA1
	PIK3CG
	PTGS2
	SYK
	HSD17B1
	PLK1
	CA13
	ST6GAL1
	CYP19A1
	AR
	CBR1
	MMP12
	CCNB3
	AVPR2
MOL004624	TLR9
	STAT3
MOL004628	CHRNA4
	ITGA2B
	CHRNA7
	CHRNA3
	BCHE
	AR
	MME
	HSD11B1
	DPP4
	PLA2G10

SLC6A3
FKBP1A
DNM1
CHRNB4
CHRNA3
HTR1A
CHRNA5
SLC6A2
PARP1
SRD5A2
PLA2G2C
GRM3
GRM8
GRM6
GRM2
HRH3
HTR1F
ADRA1A
ADRB2
ADRB1
ADRB3
HSD17B3
DRD4
NR3C1
PTGER4
PTGER2
PTGER3
GBA
GBA2
HTR1D
HTR5A
UTS2R
HTR3A
CHRNB3
SLC6A9
CYP51A1
HRH4
HTR1B
DRD1
PDE10A
EPHX1
ADRA2A
ADRA2C
ADRA2B

	HTR2C
	HRH1
	ADRA1B
	FUCA1
	HSD17B7
	REN
	HTR2B
	HTR6
	SCN5A
	KCNA5
MOL004644	ESR1
	ESR2
	SIRT1
	GLO1
	NR1H3
	PDE4B
	CD38
	ODC1
	PTGES
	PLG
	ABCC1
	SHBG
	TNKS2
	TNKS
	NAE1
	GPR35
	PTPRS
	DAPK1
	MPG
	AKR1B1
	PDE4D
MOL004648	AR
	NR1H3
	ESR2
	VDR
	CDC25A
	GLRA1
	HDAC6
	HDAC2
	HDAC1
	HDAC8
	HDAC3
	HDAC7
	HDAC11

	HDAC4
	HDAC9
	HDAC10
	RBP4
	CYP19A1
	HDAC5
	PTPN1
MOL004653	CNR1
	CNR2
	ELANE
	CSF1R
	LCK
	HCRTR2
	HCRTR1
	GSK3B
	CTSS
	RBP4
	CTSV
	CTSL
	ADORA3
	SCN2A
	SCN10A
	ELOVL6
	FLT1
	CCND3
	CXCR2
	KDR
	P2RX3
	GRM5
	JAK3
	CFD
	JAK2
	CHRM3
	SCN9A
	PPIA
	MAPK1
	PABPC1
	IDH1
	CHRM2
	CHRM1
	PRKDC
	TSPO
	PDE10A
	CPT1A

MMP9
MAPK11
VCP
BDKRB1
CCND1
EGFR
CCNE2
CCNB3
ADORA1
ADORA2A
GRM4
PTGS2
FPR1
PTPN1
NOS2
HTR1A
DRD2
CTSK
KCNJ5
KCNJ6
HTR7
HTR6
NOS1
GRK2
PSMB5
TRPA1
IKBKB
SAE1
PDE4B
MMP3
MMP1
STK33
ITGAV
PIM1
DRD4
PRKCG
CMA1
PIM2
NPY5R
TRPV1
CHRM4
CDK5
PSEN2
KCNK3

MOL004702	KCNK9
	F10
	PDE7A
	CDK2
	FAAH
	NTRK1
	CCKBR
	PDE9A
	TACR3
	NTRK2
	NTRK3
	MAPK8
	MAPK10
	MAPK9
	FAP
	P2RX7
	PTK2
	PLK1
	GRM2
	CCR1
	PTPN1
	HIF1A
	NR1I2
	HMGCR
	GSK3A
	EPHX2
	PARP1
	F2R
	INCENP
	TTK
	MDM2
	PDE3A
	PDE3B
	MMP3
	MMP1
	MMP2
	MMP7
	CCNC
	PDK1
	CDK8
	INSR
	NR1H4
	ITK
	AURKA

	P2RX3
	LIMK1
	PSEN2
	HSP90AA1
	CHEK1
	GABRB3
	GABRA1
	GABRA5
	GABRG2
	ADORA2A
	ADORA2B
	MTOR
	PIK3CB
	PIK3CA
	MAP3K14
	CYP19A1
	ROCK2
	MAPK1
	CRHR1
	CCND1
	EZH2
	SORD
	MMP9
	MMP8
	VDR
	JAK3
	REN
	IMPDH1
	IMPDH2
	PTGS1
	KDR
	IL6ST
MOL013187	SLC6A2
	SLC6A3
	SHBG
	MCL1
	MAPK9
	ALOX12
	MAP2K1
	HTR1A
	JAK3
	CDK2
	CDK1
	ALOX15

LYPLA1
LYPLA2
ERN1
HDAC2
TDP2
CHEK1
ADA
PDE2A
CHEK2
PNP
FNTA
CDK9
JAK1
MTOR
CAPN1
CYP17A1
ROCK2
ROCK1
LCK
SYK
ITK
TXK
CCND1
PYGL
FGFR1
SLC6A4
NTRK1
WEE1
ABL1
EGFR
FCER2
PIK3CD
PRKDC
PIK3CB
HCK
PIK3CG
PI4KB
PIK3CA
EPHB4
ZAP70
STAT5A
TOP1
PLA2G2A
ROCK2

		KMO
		JAK2
		PLK1
		FBP1
		TRPM8
		PIK3CA
		MMP13
		CA9
		ADAM17
		MMP14
		MMP7
		ADAM10
Chi Shao	MOL001002	GPR35
		ERBB2
		AKR1B1
		CCND1
		PDGFRB
		FLT4
		IGF1R
		INSR
		EGFR
		CA2
		CDK2
		AURKB
		CA7
		CA1
		GSK3B
		SRC
		PTK2
		KDR
		PLK1
		CA6
		CA12
		CA14
		CA9
		CSNK2A1
		MET
		CA4
		PLK4
		CA13
		TEK
		AKT1
		AURKA
		CA5A

	BACE1
	MAP3K8
	BRAF
	EPHB4
	HSPA1A
	NUAK1
	SQLE
	FGR
	LYN
	GSR
	TNNC1
	XDH
	DAO
	PTGS2
	ESR1
	ESR2
	MAOA
	HSD17B3
	PTPN1
	ALOX5
	CBR1
	CDK5
	CES2
	CA5B
	SNCA
MOL001925	ABCB1
	HSP90AA1
	LGALS3
	LGALS9
	SLC6A3
	PTAFR
	SELP
	PRKCG
	PRKCD
	PRKCA
	PRKCB
	PRKCE
	PRKCH
	PRKCQ
	FDFT1
MOL002714	KDM4E
	XDH
	ALOX15
	CDK1

ALOX12
GRK6
CYP19A1
CA7
CA12
CA4
ABCB1
CYP1B1
HSD17B1
AKR1B1
CDK5
CA2
CCNB3
CDK6
CA1
CA9
ABCG2
CBR1
ESR2
ESR1
ACHE
ADORA1
ADORA2A
PTGS2
PTPRS
AMY1A
FLT3
IKBKB
NTRK2
AR
NOX4
MAOA
SYK
GSK3B
ABCC1
TTR
CSNK2A1
CFTR
AKR1B10
TNKS2
TNKS
HSD17B2
BCHE
ADORA3

TERT
LCK
PFKFB3
PIM1
PARP1
ALOX5
APP
CALM1
ARG1
NOS2
GLO1
MMP9
MMP2
MMP12
CD38
TOP1
NAE1
EGFR
SRC
TYR
AHR
ESRRA
KIT
OPRD1
CYP1A1
AURKB
PIK3CG
MAPT
TOP2A
INSR
MYLK
APEX1
IGF1R
KDR
PLK1
MET
ALK
AXL
PTPN1
ST6GAL1
SLC22A12
GPR35
FYN
TACR2

	PRKDC
	MAPK3
	BACE1
	SIRT1
	PLA2G2A
	PLA2G4A
	CA6
	CDK2
MOL006992	CA7
	CA12
	CA4
	MMP12
	MMP13
	MET
	CA2
	CA1
	CA3
	CA6
	CA5B
	CA5A
	KIT
	SRC
	KDR
	FGFR1
	CYP1B1
	KLK1
	KLK2
	BCL2
	DNMT1
	KCNH2
	APP
	MAPK14
	TERT
	PGD
	ST3GAL3
	FUT7
	FUT4
	STAT1
	SQLE
	CA9
	PTGS1
	CA13
	ABCC1
	TAS2R31

	ESR1
	ESR2
	ADORA1
	MMP2
	BACE1
	ABCB1
	CYP19A1
	RXRA
	MMP14
	MAPT
	GABRA1
	ACHE
	PLA2G1B
	DYRK1A
	HIF1A
	CES1
	PPARG
	CES2
	HSD17B1
	AKR1C3
	ABCG2
	PGF
	VEGFA
	SHBG
	MMP9
	MT-ND4
	POLB
	PLA2G2A
	PLA2G5
	PLA2G10
	ADORA3
	GRIN2A
	CBR1
	PYGL
MOL007008	HSP90AA1
	LGALS3
	LGALS9
	ABCB1
	SLC6A2
	SSTR5
	SSTR2
	SSTR4
	SSTR1
	SSTR3

		VEGFA
		FGF1
		FGF2
		HPSE
		PTAFR
		AKR1B1
	MOL007012	HSP90AA1
		LGALS3
		LGALS9
		ABCB1
		SLC6A2
		SSTR5
		SSTR2
		SSTR4
		SSTR1
		SSTR3
		VEGFA
		FGF1
		FGF2
		HPSE
		PTAFR
		AKR1B1
	MOL007016	ABCB1
		HSP90AA1
		LGALS3
		LGALS9
		SLC6A3
		PTAFR
		SELP
		PRKCG
		PRKCD
		PRKCA
		PRKCB
		PRKCE
		PRKCH
		PRKCQ
		FDFT1
Chuan Xiong	MOL002135	EGFR
		FYN
		EPHA2
		YES1
		BLK
		LCK
		SYK

CSK
EPHB2
BMX
LYN
EPHA5
EPHA4
TXK
FGR
EPHA6
PTK6
EPHB3
EPHA3
BTK
TYRO3
COQ8B
EPHA1
RET
PIM1
ALOX5
PRKCG
PIM2
CNR1
PARP1
CA9
PIM3
FPR1
HSP90AA1
CDK2
HDAC7
CTSV
FLT3
PDGFRA
MYLK
GCGR
HSP90AB1
RAF1
CYP11B1
FLT4
DYRK1A
CYP11B2
STAT6
NPY5R
CYP17A1
CXCR2

CNR2
JAK2
DHFR
STAT3
EGLN1
QPCT
HSD17B3
DRD3
ADORA2A
ERBB2
FLT1
GABRA1
CDK4
GSTA1
KCNMA1
ROCK2
ROCK1
PLA2G7
MAP2K2
TACR3
KIF11
NOX4
CSF1R
MIF
FAAH
NCOR1
PDE10A
CDC25B
TRPM8
HDAC3
MAPKAPK2
ADORA1
PIK3CD
PIK3CG
HDAC11
HDAC10
ABL1
PRKCA
CDK1
CMA1
RPS6KB1
AURKA
ADORA2B
TYMS

	GSK3B
	MKNK1
	TNKS2
	TNKS
	SLC29A1
MOL002151	ALOX5
	CYP19A1
	PLK1
	GLI2
	NR3C1
	SOAT1
	HCRTR2
	HCRTR1
	PTGS2
	ACACB
	CYP24A1
	CYP27A1
	DKK1
	OPRD1
	KDR
	TEK
	ITK
	PDE4B
	PDE7A
	NAMPT
	F10
	NPY5R
	HSD17B2
	GRM5
	CRHR1
	PFKFB3
	KCNA5
	LYPLA2
	EPHX2
	ACACA
	LSS
	IMPDH2
	MGAT2
	KCNH2
	DGAT2
	GPR88
	SMO
	GPR183
	THRA

THRB
PARP1
CNR1
PTGER4
MTNR1A
MTNR1B
SYK
ABCC1
CTSV
CTSL
ABCB1
BDKRB2
HTR1A
DRD4
HTR2A
HTR2C
DRD3
HTR7
HTR6
UTS2R
FASN
C3AR1
ICAM1
SELE
HRH1
CTSK
TGM2
CTSS
F2R
F13A1
MDM2
DRD2
ELANE
CFTR
PDE10A
SLC5A1
POLM
CCKBR
POLK
POLL
POLH
HPGDS
MAPK10
SRC

	SCN9A
	TRPV1
	SCARB1
	PSEN2
	MMP9
	KIF11
MOL002157	GRM5
	CTSK
	CTSS
	PDE10A
	CPT1A
	CPT2
	PTGER1
	SLC6A9
	CMA1
	F10
	CAPN1
	FAP
	HCRTR2
	HCRTR1
	CTSL
	CTSB
	ABL1
	SRC
	TSPO
	CCND1
	PIM1
	TACR3
	KIF11
	SLC2A1
	SIGMAR1
	PIM2
	SLC2A3
	MAPK14
	FLT3
	DRD4
	MAPK8
	MAPK10
	FKBP1A
	PSEN2
	PTK2
	P2RX7
	CPT1B
	PYGL

PYGM
EPHX2
MAPK9
ADORA2A
HTR7
HTR6
ADRA1D
ADRA1A
ADRA1B
SLC6A1
CNR1
HTR2A
PPIA
TRPA1
CAPN1
CAPN2
PDE7A
HCK
CTSV
S1PR3
OXTR
DRD3
CHRNA7
ADORA3
PARP1
SCN9A
MME
HTR2B
CHRM1
JAK1
ACE
ALK
WNT3A
KCNH2
TNKS2
CCNE2
CCNB3
MMP13
SCARB1
CASP3
PDE4B
S1PR1
STAT3
PSENEN

		GPR139
		NQO2
		LGMN
		PTK2B
		KCNJ11
		CHRM2
		CTRB1
		SLC2A2
		CCR1
		KCNJ5
		KCNJ6
		AKT1
		QPCT
		ECE1
		PTPN1
		JAK3
		SYK
		CSNK1D
Dang Gui	MOL000358	NPC1L1
		NR1H3
		RORC
		SHBG
		HMGCR
		CYP17A1
		SREBF2
		CYP19A1
		AR
		CYP51A1
		RORA
		ESR1
		ESR2
		PTPN1
		CYP2C19
		ACHE
		SERPINA6
		G6PD
		SLC6A2
		SLC6A4
		BCHE
		CHRM2
		NR1I3
		NR1H2
		VDR
		PTGER1

	PTGER2
	TBXAS1
	PTGES
	DHCR7
	PPARD
	SQLE
	GLRA1
	HSD11B1
	PTPN6
	PTPN2
	FDFT1
	CES2
	NOS2
	PPARG
	UGT2B7
	NR3C1
	POLB
MOL000449	NPC1L1
	NR1H3
	RORC
	ESR1
	ESR2
	SHBG
	SREBF2
	HMGCR
	CYP19A1
	AR
	CYP17A1
	CYP51A1
	RORA
	CYP2C19
	PTPN1
	SERPINA6
	G6PD
	ACHE
	SLC6A4
	NR1I3
	CHRM2
	SLC6A2
	BCHE
	NR1H2
	PTGER1
	PTGER2
	TBXAS1

		PTGES
		PPARA
		PPARD
		SQLE
		VDR
		DHCR7
		PTPN6
		PTPN2
		FDFT1
		HSD11B1
		NOS2
		PPARG
		UGT2B7
		GLRA1
		POLB
Gan Cao	MOL000098	ABCG2
		AKR1B1
		NOX4
		PLG
		ADORA3
		XDH
		CA2
		CA7
		CA12
		CA4
		CYP1B1
		APP
		ADORA1
		ABCB1
		ADORA2A
		MAPT
		KDM4E
		GPR35
		AVPR2
		TOP2A
		MAOA
		IGF1R
		FLT3
		CYP19A1
		INSR
		EGFR
		F2
		PIM1
		ALOX5

AURKB
DRD4
ACHE
GLO1
MYLK
MPO
PIK3R1
DAPK1
PYGL
SYK
CA1
GSK3B
SRC
PTK2
HSD17B2
KDR
MMP13
MMP3
CA3
ALOX15
ABCC1
PLK1
CA6
CDK1
MMP9
PIK3CG
MMP2
PKN1
CA14
CA9
CSNK2A1
ALOX12
MET
NEK2
CXCR1
CAMK2B
ALK
AKT1
NEK6
PLA2G1B
CA5A
AXL
APEX1
NUAK1

	AKR1C2
	AKR1C1
	AKR1C3
	AKR1C4
	CA13
	AKR1A1
	PTGS2
	PLA2G2A
	PTPRS
	ESR2
	MPG
	SLC22A12
	OPRM1
	ODC1
	CDK5R1
	CCNB3
	CDK6
	CDK2
	ARG1
	TERT
	NOS2
	TYR
	HSD17B1
	AHR
	ESRRA
	MCL1
MOL000211	SAE1
	POLB
	AKR1B10
	HSD11B1
	PTPN1
	PTGES
	NR1H4
	CDC25C
	PTPN2
	GPBAR1
	TOP2A
	CDC25A
	RORC
	PTPRF
	ACP1
	PDE4D
	PLA2G1B
	CDC25B

	CES2
	AR
	TERT
	FAAH
	FABP3
	FABP5
	PPARD
	FABP1
	PTPN11
	UGT2B7
	SCD
	SERPINA6
	SHBG
	G6PD
	CYP51A1
	CYP19A1
	HSD17B3
	GABBR1
	TOP1
	NPC1L1
	SIGMAR1
	CYP17A1
	VDR
	GABRA2
	PPARG
	PTPN6
	SLC6A3
	ADORA3
	MAPK3
	FFAR1
	FABP2
	PTGS2
	CD81
	PRKCH
	HSD11B2
	HSF1
MOL000239	AKR1B1
	ADORA1
	ADORA2A
	NOX4
	ABCB1
	MCL1
	ABCG2
	ESR2

SLC22A12
OPRD1
HSD17B2
HSD17B1
FLT3
BACE1
CYP1B1
ADORA3
APP
PIM1
PLG
EGFR
KIT
PIK3CG
ALK
XDH
NOS2
PTGS2
OPRM1
CA2
CA7
CA12
MAPT
TOP2A
INSR
MYLK
APEX1
ESR1
AKT1
ALOX5
ODC1
MMP9
MMP2
CA4
MET
ABCC1
IGF1R
AVPR2
CXCR1
ALOX15
ALOX12
TYR
AHR
ESRRA

CYP19A1
CA1
CA6
CA9
NAE1
F2
CDK1
GPR35
GSK3B
PTPRS
KDM4E
MAOA
ACHE
TERT
AURKB
SRC
PTK2
KDR
PLK1
PKN1
NEK2
NEK6
AXL
NUAK1
CDK5
CCNB3
CDK6
ST6GAL1
KCNA3
PDE5A
LCK
SYK
CSNK2A1
HSP90B1
BCHE
PLA2G2A
DAPK1
MPG
CYP1A1
CYP1A2
CAMK2B
GRK6
CDK2
PFKFB3

	GCGR
	DRD4
	GLO1
	AMY1A
MOL000354	XDH
	CA2
	CA7
	CA12
	CA4
	CYP1B1
	ABCC1
	NOX4
	AKR1B1
	ABCG2
	ACHE
	ALOX15
	ALOX12
	IGF1R
	EGFR
	AVPR2
	MAOA
	FLT3
	CYP19A1
	F2
	PIM1
	ALOX5
	AURKB
	DRD4
	ADORA1
	GLO1
	MPO
	PIK3R1
	ADORA2A
	DAPK1
	PYGL
	CA1
	GSK3B
	SRC
	PTK2
	HSD17B2
	KDR
	MMP13
	MMP3
	CA3

PLK1
CA6
CDK1
MMP9
MMP2
PKN1
CA14
CA9
CSNK2A1
MET
NEK2
CXCR1
CAMK2B
ALK
AKT1
ABCB1
NEK6
PLA2G1B
CA5A
BACE1
AXL
NUAK1
AKR1C2
AKR1C1
AKR1C3
AKR1C4
CA13
AKR1A1
GPR35
MAPT
KDM4E
TOP2A
INSR
MYLK
SYK
PIK3CG
APEX1
CDK5
CCNB3
CDK6
CDK2
ARG1
APP
MCL1

	TERT
	TYR
	HSD17B1
	AHR
	ESRRA
	PTPRS
	PLG
	ESR2
	MPG
	SLC22A12
	PARP1
	TTR
	MMP12
	CD38
	AKR1B10
	TNKS2
MOL000392	IL2
	CYP19A1
	ALDH2
	CA12
	ADORA1
	ADORA2A
	TBXAS1
	MAOA
	MGAM
	HTR2A
	HTR2C
	ESRRA
	ESRRB
	ABCG2
	CA7
	HSD17B2
	CA4
	CBR1
	SLC6A2
	ALOX12
	EGFR
	TYR
	XDH
	PFKFB3
	ESR2
	PTPRS
	HSD17B1
	ABCB1

	ESR1
	MIF
	ALOX15
	PPARA
	PTGS1
	TLR9
	MAOB
	CA2
	CA1
	NOX4
	PON1
	TNKS2
	TNKS
	ACHE
	CA3
	CA6
	CA14
	CA13
	CA5B
	CA5A
	DHODH
	STS
	ERCC5
	FEN1
	RAF1
	CA9
	PTPN1
	BACE1
MOL000417	CYP19A1
	CA7
	HSD17B2
	CA12
	CA4
	CBR1
	ABCB1
	TYR
	MIF
	PPARA
	HSD17B1
	ESR1
	ESR2
	TLR9
	PTPRS
	EGFR

	MAOB
	ALDH2
	XDH
	ALOX12
	PTGS1
	SLC6A2
	TBXAS1
	MAOA
	MGAM
	HTR2A
	HTR2C
	ADORA1
	ADORA2A
	ESRRA
	ESRRB
	ABCG2
	CA2
	IL2
	PFKFB3
	NOX4
	ALOX15
	CA1
	PTPN1
	PON1
	ABCC1
	CYP1B1
	DUSP3
	CDC25B
	CDC7
	PLAT
	F10
	PLAU
	ACHE
	STS
	CA5B
	HSP90AB1
MOL000422	NOX4
	AKR1B1
	XDH
	TYR
	FLT3
	CA2
	ALOX5
	CA7

HSD17B2
ABCC1
HSD17B1
AHR
CA12
ESRRA
ABCB1
CYP1B1
ABCG2
ADORA1
CA4
ACHE
MAOA
GLO1
SYK
GSK3B
MMP9
MMP2
ALOX15
ALOX12
PTPRS
ADORA2A
CDK5
CCNB3
ARG1
GPR35
ESR2
DAPK1
MPG
SLC22A12
TTR
AKR1B10
TNKS2
TNKS
CDK6
CDK2
CYP19A1
CSNK2A1
EGFR
AVPR2
IGF1R
F2
PIM1
AURKB

DRD4
MPO
PIK3R1
PYGL
CA1
SRC
PTK2
KDR
MMP13
MMP3
CA3
PLK1
CA6
CDK1
PKN1
CA14
CA9
MET
NEK2
CXCR1
CAMK2B
ALK
AKT1
NEK6
PLA2G1B
CA5A
BACE1
AXL
NUAK1
AKR1C2
AKR1C1
AKR1C3
AKR1C4
CA13
AKR1A1
APP
PARP1
MMP12
CD38
TOP1
ESR1
PTGS2
CFTR
PFKFB3

	AMY1A
	GRK6
	TERT
	MAPT
MOL000497	ALOX5
	PDPK1
	ABCG2
	AKR1B1
	CYP19A1
	MAOB
	PTPN1
	CHRNA7
	BACE1
	PTGS2
	NOS2
	APP
	F3
	PTGS1
	TYR
	SLC5A1
	ODC1
	ACHE
	EGFR
	SLC5A2
	PDK1
	KCNA3
	PLA2G1B
	MAOA
	SLC29A1
	ABCB1
	NPY5R
	MAPKAPK2
	CTSD
	THRA
	THRB
	HDAC6
	HDAC8
	HDAC1
	HDAC3
	PDGFRB
	HDAC5
	RPS6KB1
	RET
	MAPK14

MMP9
MMP8
CAPN1
HPGDS
NR1H4
ESRRB
HDAC7
HSP90AB1
HDAC10
RAF1
CYP11B1
HTR1A
CYP11B2
BRAF
FASN
ERN1
ITGAL
HDAC4
HDAC9
TRPM8
PSEN2
NAAA
BCHE
FLT3
NCOR2
ITK
JAK2
MMP1
IMPDH1
MAPK10
MAPK1
ROCK2
HDAC11
ADORA2B
ADAM17
ALOX5AP
PARP2
BMP1
MDM2
MMP13
TACR3
ADAM10
STAT6
TGFBR1

	HCRTR2
	MDM4
	ECE1
	CDC25B
MOL000500	ALOX15
	ALOX12
	CYP19A1
	MAOB
	ALOX15B
	TYR
	RET
	CHEK1
	WEE1
	GSK3B
	PIM1
	PIM2
	HTT
	COMT
	MIF
	HSD17B3
	NOX4
	CLK1
	DYRK1B
	VCP
	CASP3
	CASP7
	RAF1
	MAP2K1
	BRAF
	PIM3
	ESR1
	ESR2
	CCND3
	ADAM17
	MKNK1
	KDR
	MAPKAPK2
	ADORA2B
	RPS6KA2
	CDK4
	CDK2
	HSP90AA1
	MMP7
	ADORA2A

ALPL
HSD17B2
PITRM1
HSP90AB1
MMP25
MMP16
ESRRA
ESRRB
BMP1
ADAM10
LNPEP
CTSS
MTOR
PIK3CD
PIK3CB
PIK3CG
PIK3CA
EZR
ABL1
ADRA2C
ADRA2B
PIK3CA
TUBB1
TUBB3
CA14
HDAC3
ROCK1
MBD2
SIGMAR1
AURKA
CHRNA7
CAPN1
SRC
DBF4
ROCK2
ALPG
MPEG1
GSTP1
XIAP
GSTM2
PLAA
PTGS1
NR4A1
CDK5

	BCHE
	CCNB3
	JAK3
	LCK
	ALK
	DNM1
	TRPM8
	CDK2
	AKR1A1
	GSK3A
	PRKDC
	HCK
	DRD2
	CCNE1
	HDAC2
	NCOR2
MOL001484	ALOX15
	PTPN1
	ESRRA
	ESRRB
	ERN1
	AR
	RPS6KA3
	EZR
	CLK1
	DYRK1B
	TRPM8
	CHEK2
	RPS6KA1
	HSD17B2
	LNPEP
	CHEK1
	WEE1
	F3
	PGF
	VEGFA
	PARP1
	HSD17B3
	TBK1
	MIF
	MAPKAPK2
	ADAM17
	RET
	IMPDH1

	IMPDH2
	TUBB1
	ADCY5
	SRC
	TUBB3
	LCK
	DAO
MOL001792	CYP19A1
	HSD17B1
	ESR1
	ESR2
	CA7
	ABCC1
	CA12
	SHBG
	CA4
	CYP1B1
	CBR1
	MAOB
	PTGS1
	ADORA1
	ADORA3
	ABCG2
	TAS2R31
	AKR1C3
	PLA2G1B
	CES1
	PPARG
	CES2
	MMP13
	MMP12
	SLC5A2
	POLB
	PLA2G5
	PLA2G10
	GRM5
	SERPINE1
	RXRA
	BACE1
	EDNRA
	KLK1
	KLK2
	ESRRA
	ESRRB

CLK1
DYRK1B
HSD17B14
BCHE
CHRNA7
ACHE
DYRK1A
CA2
CA1
CA3
CA6
CA13
CA5B
CA5A
KIT
SRC
KDR
FGFR1
MET
CTSB
F3
HSD17B2
ERN1
RPS6KA5
AKR1B1
PLA2G2A
MMP3
SIRT2
YWHAG
WEE1
DNM1
PTGER1
PTGER4
PTGER2
PTGER3
MMP2
NOX4
CA9
PTPN1
BCL2L1
VCP
NQO2
GRM2
IGF1R

	INSR
	DUSP3
	PDPK1
	GSK3B
	PIK3CB
	CYP2C9
	CYP3A4
	PIK3CA
	CCNE1
	CCNE1
	AURKA
	CALM1
	HNF4A
	MMP9
	HDAC5
	HDAC7
	HDAC9
	PGF
	VEGFA
MOL002311	PDE4D
	ALOX15
	XPO1
	CCR4
	CA13
	HSP90B1
	SRD5A1
	NFKB1
	FNTA
	HSP90AB1
	HSP90AA1
	GPR84
	CFTR
	NOS2
	ESR2
	ALOX12
	EP300
	MPI
	EGLN1
MOL002565	PTPN1
	CYP19A1
	ESR1
	ESR2
	RET
	JAK3

LCK
ALK
KDR
MAP2K1
CHEK1
WEE1
ALOX15
PIM1
PIM2
GSK3B
KIT
PDE4D
PARP1
HSD17B3
MKNK1
MMP1
DAO
MAPKAPK2
EIF4A1
ERN1
PTGS1
HDAC2
HTT
COMT
HSD17B2
MIF
LNPEP
ALOX12
FGFR1
CCND3
CASP3
CASP7
CA14
MET
VCP
TYMS
CA2
RAF1
CYP11B1
ADORA2B
CYP11B2
CYP17A1
ABL1
MTOR

PIK3CD
PRKDC
PIK3CB
HCK
PIK3CG
PIK3CA
PDE4A
PDE4B
PDE4C
SRC
GSTP1
GSTM2
MMP3
RPS6KA2
ALPL
ADAM17
CA1
CA12
CA9
CA4
CA5B
CA5A
TUBB1
TUBB3
CDK2
CDK4
MMP7
MMP8
ALPG
MBD2
PLAA
TNKS2
TNKS
KDM4C
ABCB1
KCNMA1
DGAT1
CDK5
CLK1
CLK3
DYRK2
PLK1
HSD17B14
IRAK4

	PIK3CA
	RPS6KA5
	MAOB
	GRIA1
	PIK3R1
	ROCK2
MOL003656	PTPN1
	ESR2
	ESR1
	PDE5A
	AKT1
	HSD17B1
	ABCB1
	TBXAS1
	MGAM
	HTR2A
	HTR2C
	ESRRA
	ESRRB
	EGFR
	RELA
	CYP19A1
	CCR4
	ALOX15
	ALDH2
	HSD17B2
	ALOX12
	BCL2
	BACE1
	HSP90B1
	ACHE
	HSP90AA1
	MAOA
	HSP90AB1
	IL2
	CA4
	GPR84
	SIRT1
	GCGR
	MIF
	TYR
	XDH
	ABCG2
	PFKFB3

	PDE10A
	CA12
	FNTA
MOL003896	CYP19A1
	ABCG2
	CA12
	SLC6A2
	CA4
	MAOA
	HSD17B2
	CA7
	CBR1
	ABCB1
	EGFR
	ADORA1
	ADORA2A
	HTR2A
	HTR2C
	PTGS1
	MAOB
	CA2
	CA1
	ESR1
	TBXAS1
	HSD17B1
	MGAM
	ESRRB
	PFKFB3
	ALDH2
	TNKS
	ESRRA
	NTRK1
	TYR
	IL2
	ESR2
	CYP1B1
	PGK1
	MGLL
	ACHE
	FLT1
	PTPRS
	GRM5
	GRM1
	TACR1

MCL1
MMP1
FNTA
SLC5A7
NAAA
TRPM8
CRHR1
PDGFRA
HCRTR2
HCRTR1
PON1
HTR7
CYP17A1
UTS2R
P2RX7
MTNR1B
PRKCG
PRKCA
FPR2
CYP27A1
RPS6KB1
AURKA
JAK3
JAK2
PDE4A
PDE4C
ROCK2
PGGT1B
PPARG
PDE1B
GRM2
LRRK2
PDE10A
CXCR2
ALOX5
PDE7A
MTNR1A
TGM2
NOS1
SIRT3
SIRT2
KDM1A
NR3C2
NR3C1

	PGR
	GPR139
	ALOX12
	PSEN2
	CHRNA7
	TGFBR1
	MMP8
	CA9
	CACNA2D1
	PARP2
	ABAT
	MIF
	HDAC6
	F10
	PIK3CB
MOL004328	CYP19A1
	CA7
	ABCC1
	HSD17B1
	CA12
	SHBG
	CA4
	CYP1B1
	CBR1
	ESR1
	ESR2
	PTGS1
	MAOB
	ADORA1
	ADORA3
	ABCG2
	TAS2R31
	AKR1C3
	PLA2G1B
	GRM5
	CES1
	PPARG
	CES2
	SLC5A2
	MMP12
	POLB
	MMP13
	PLA2G2A
	PLA2G5

PLA2G10
BACE1
CHRNA7
KLK1
KLK2
RXRA
SERPINE1
SRC
CA2
CA1
HSD17B14
KIT
KDR
FGFR1
MET
CA3
CA6
CA13
CA5B
CA5A
NQO2
CTSB
NOX4
AKR1B1
CLK1
DYRK1B
BCHE
CA9
ESRRA
ESRRB
CDK5
IGF1R
INSR
DYRK1A
HSD17B2
IGFBP3
MMP2
YWHAG
SIRT2
F3
PTGER1
PTGER2
PTGER3
BCL2L1

	PIK3CB
	CYP2C9
	CYP3A4
	PIK3CA
	VCP
	PGF
	VEGFA
	EDNRA
	ERN1
	MMP3
	APP
	GSK3B
	BCL2
	MMP9
	WEE1
	LCK
	SYK
	CCNE1
	CDK3
	CDK4
	AURKA
	SNCA
	ALOX12
	HNF4A
MOL004805	CTSK
	ODC1
MOL004806	ESR1
	ESR2
	PPARG
	ABCG2
	CYP19A1
	CES1
	BCHE
	CES2
	ACHE
	CTSK
	MAOB
	CA12
	HSD17B1
	PLA2G1B
	ODC1
	BACE1
	PTGS1
	CTSL

ADORA1
HSP90AB1
ADORA3
TAS2R31
PDE10A
RXRA
CYP1B1
MTNR1A
HSP90AA1
MTOR
PIK3CA
SHBG
MAPK1
TRPV1
MARS
CDK2
MMP3
MMP1
MMP7
MMP8
CA7
CA4
HSD17B2
HCRTR2
HCRTR1
PARP1
RET
PSEN2
PFKFB3
CASR
ADAM17
EGFR
DBF4 CDC7
P2RX7
BRD4
BRD3
TSPO
BRPF1
PRKCZ
CCR3
CCKBR
CDK5
CCNE2
CCNB3

	HSD11B1
	BACE2
	NQO2
	FLT4
	FLT3
	PDGFRA
	MAP2K2
	ALK
	RAC1
	CRHR1
	CCND1
	CDK1
	CCNE1
	NPY1R
	MTNR1B
	LDLR
	RAF1
	PIM1
	BRAF
	HTR1B
	NR3C1
	DRD4
	ADRA1D
	ADRA1A
	ADRA1B
	MYLK
	HTR7
	CAPN1
	OPRK1
	CAPN2
	CAPN1
	GRIA1
	NTSR1
	TGFBR1
	TYMS
	F9
	RPS6KA3
	CXCR2
MOL004808	PTPN1
	TYR
	MAOB
	ESR1
	PDGFRB
	ABCG2

PLK1
ADORA2B
BMP1
AKR1B10
ESR2
CTSL
MTOR
PIK3CG
PIK3CA
GSK3B
GSK3A
CES2
CASP3
CASP7
COMT
RET
HSD17B2
HSP90AA1
RAF1
MYLK
HDAC8
HDAC1
PIM2
BRAF
MARK1
MMP1
ADAM17
FLT3
DUSP3
CHEK1
PDK1
WEE1
KDM1A
ADORA2A
CDK2
MMP3
CASP6
HDAC4
ERBB2
PPARG
EP300
ABL1
HDAC6
PDE10A

KIT
KDR
CYP19A1
ACACB
ALOX15
CHRNA7
HDAC3
LCK
SYK
LYN
MMP7
TXK
IMPDH1
PIM1
HSD17B3
KLK1
KLK2
STAT3
CPT1A
SCD
ALPL
MME
HDAC2
NCOR2
LIPC
JAK3
HDAC11
HSD11B1
ALK
LIPG
HDAC10
SAE1 UBA2
DRD4
MCL1
TGM2
DPP4
ROCK2
ROCK1
CDC25A
PLAA
AR
CDK1
CCNE1
ANPEP

	CDK3
	AURKA
	MT-ND4
	PDE5A
	CPT2
	ME1
MOL004810	ODC1
	MAOB
	AURKB
	RPS6KB1
	AURKA
	ESR1
	ESR2
	PTPN1
	SLC5A1
	BRAF
	HSD17B2
	CTSK
	HDAC1
	SGK1
	KDM1A
	DNM1
	SYK
	HDAC5
	HDAC7
	TGM2
	HDAC4
	MMP8
	EPHB4
	NOX4
	TNF
	PARP1
	RET
	DRD1
	CHRNA7
	LCK
	MAPK14
	CDK1
	CCNE2
	PLG
	HDAC8
	TRPM8
	SLC29A1
	HTR7

	ADAM17
	BCL2L1
	BCL2
	PDK1
	CDK2
	CDK3
	NFKB1
	GRK2
	RELA
MOL004811	TYR
	PTPN1
	ALOX15
	ESRRA
	ESRRB
	HSD17B2
	ALOX15B
	PDK1
	CTSD
	DNM1
	CTSK
	CYP2C9
	CYP3A4
	ALOX12
	AURKA
	RET
	LCK
	CYP1A2
	AURKB
	ADORA2B
	RPS6KB1
	MDM2
	SLC29A1
	ABL1
	HSP90AA1
	HDAC3
	PDGFRB
	HDAC2
	PDGFRA
	HDAC5
	CAPN1
	PIK3CD
	PIK3CG
	TRPV1
	RAF1

	MMP1
	BRAF
	MMP8
	HPGDS
MOL004814	ALOX5
	CBR1
	ESR1
	ESR2
	MAOA
	CA7
	CA12
	CA4
	CA2
	AKR1B1
	CA1
	CA6
	CA14
	CA9
	CA13
	CA5A
	ACHE
	PARP1
	TNKS2
	PTGS2
	NOX4
	CCNB3
	CDK6
	SYK
	ABCC1
	HSD17B1
	CFTR
	AKR1B10
	GSK3B
	CSNK2A1
	TBXAS1
	MGAM
	HTR2C
	ESRRA
	ESRRB
	EGFR
	TYR
	AHR
	XDH
	TERT

CDK2
ALOX12
ARG1
MCL1
GLO1
APP
MMP9
MMP2
MMP12
CD38
TOP1
PFKFB3
SNCA
IGF1R
CA5B
AURKB
IGFBP3
CRHR1
CDK5
DHODH
DAPK1
MPG
SLC22A12
KCNA5
KCNA3
KDR
PLK1
MET
GPR35
FNTA
AVPR2
F2
DRD4
MPO
PIK3R1
PYGL
MMP13
MMP3
CDK1
PKN1
NEK2
CXCR1
CAMK2B
NEK6

	PLA2G1B
	AKR1C2
	AKR1C4
	AKR1A1
	IGFBP6
	IGFBP4
	IGFBP5
	IGFBP2
	IGFBP1
	SRC
	PTK2
	AKT1
	BACE1
	NUAK1
	GSR
	CYP1A2
MOL004815	ODC1
	MAPKAPK2
	MAPKAPK5
	MAOB
	NOS2
	PTPN1
	BCL2
	APP
	ALOX5
	PRKCD
	AKR1B1
	PDPK1
	CCR4
	KCNA3
	CHRNA7
	WEE1
	GPR84
	ABCG2
	ALPL
	F3
	MAOA
	MCL1
	TERT
	CYP19A1
MOL004820	PDE4D
	CA1
	CA12
	CA9

CA7
GSK3B
MMP9
MMP1
MMP2
ODC1
ERN1
ELANE
AURKA
PTPN1
HDAC3
HDAC1
CTSL
ALDH2
MET
GCGR
HPGDS
SREBF2
MAOB
AURKB
CA13
PARP1
HDAC6
CES1
PPARG
CES2
HDAC8
SCD
GRK6
COMT
CYP1A2
GRIN1
SRC
HIF1A
TNNC1
CDC25A
CDC25B
ALOX5
TRPV1
EPHX2
AR
APP
MCL1
TAS2R31

TBXA2R
CCNB3
RAF1
TGFBR1
BRAF
ACVR1
PLEC
S1PR1
CYP1B1
FLT3
GSK3A
CDK2
ADAMTS5
ADAMTS4
MMP13
MMP14
HSP90AA1
HDAC2
KDR
IKBKE
TBK1
CDK5
DPP4
CTSB
CHEK1
BMP4
HTR3A
CHRNA7
EP300
KAT2B
KAT2A
NCOR2
HDAC11
PDE10A
PDK1
HDAC10
MPEG1
HSD11B1
KIF11
BCL2
PLAA
BMP1
STK17B
MDH2

	MMP3
	ADAM17
	MMP7
	MMP12
	MMP8
	PRF1
MOL004824	MMP13
	MMP9
	MMP2
	CDK1
	CCNE1
	KDR
	GRM5
	CSF1R
	TYMS
	SOAT1
	SOAT2
	F2
	NR3C1
	SCN9A
	RPS6KB1
	AURKA
	MMP1
	HIF1A
	KCNH2
	MET
	CHEK1
	BACE2
	PIK3CG
	HDAC3
	JAK3
	JAK1
	OPRK1
	SLC5A1
MOL004827	PTPN1
	CYP19A1
	RARA
	GCGR
MOL004828	ABCB1
	PDE5A
	AKT1
	PTPN1
	CYP19A1
	ALOX15

ABCG2
BACE1
ESR2
ALK
HSD17B2
HSD17B1
HSP90B1
HSP90AA1
CDK1
HSP90AB1
XDH
CA7
CYP1B1
TYR
ABCC1
AHR
ESRRA
MCL1
ALOX12
CA12
EGFR
CA2
EGLN1
ESR1
EP300
BCHE
ACHE
GSK3B
PTPRS
DAPK1
MPG
AKR1B1
CA4
CBR1
MAPT
KDM4E
TOP2A
F2
MYLK
MPO
PIK3R1
PYGL
SYK
MMP13

MMP3
CA3
PLK1
CSNK2A1
NEK2
CA13
NEK6
PLA2G1B
CA5A
APEX1
AKR1C2
AKR1C1
AKR1C3
AKR1C4
ALPL
NOX4
PTGS2
AVPR2
CXCR1
PLAU
PDE10A
ERBB2
PFKFB3
CCND1
GCGR
CA1
CA9
GPR84
PDK1
CDK2
CFTR
DHFR
CNR2
ADORA2A
GABRB3
GABRB3
MMP9
MMP2
ARG1
BCL2
GPR35
MELK
ALOX5
ADORA1

	NAE1
	SLC22A12
	PTAFR
	CA6
	FNTA
MOL004833	PTPN1
	GSK3A
	ADORA1
	LNPEP
	HSD17B2
	HSD17B1
	AR
	ESR1
	ESR2
	MMP1
	MET
	CCNB3
	KIT
	SRC
	KDR
	FGFR1
	HSD17B3
	LRRK2
	GRK7
	HIPK4
	TAOK2
	PIK3C2G
	PIP4K2C
	ERBB2
	FYN
	CSF1R
	ABL1
	FLT1
	CASK
	PIP5K1C
	DSTYK
	PDGFRB
	FLT4
	FLT3
	PDGFRA
	EGFR
	RET
	EPHA2
	YES1

GABRB3
GABRA1
GABRA5
GABRG2
MAP2K3
PIM1
JAK3
BLK
RPS6KA3
PHKG2
CSNK1G1
MYLK
DAPK3
CHEK2
CSNK1G2
RPS6KA1
DAPK1
LCK
CSK
FGFR3
CSNK1A1
NTRK1
CSNK1D
JAK1
MTOR
MAP3K9
PRKG2
MAP2K4
MAP2K2
JAK2
CDK7
DAPK2
RPS6KA4
PIK3CD
PRKDC
PIK3CB
HCK
PIK3CG
PI4KB
EPHB2
IRAK1
TYK2
PRKCE
MAP2K1

	PRKCH
	CSNK2A1
	IRAK4
	PLK4
	AAK1
	MARK2
	BMX
	MAP3K20
	LYN
	STK16
	MAP3K1
	FGFR4
	STK17B
	STK10
	TTK
	EPHA5
	EPHA4
MOL004835	MAOB
	ABCG2
	PTPN1
	AKR1B1
	APP
	CHRNA7
	F3
	MAOA
	CYP19A1
	ALOX5
	KCNA3
	BACE1
	ALDH2
	PDPK1
	PTGS2
	EGFR
	TUBB1
	NOS2
	PFKFB3
	MIF
	ABCB1
	RET
	MMP9
	MMP1
	HTR1A
	TERT
	BCHE

ADAM17
CHEK1
EZR
MMP13
MMP3
MMP7
MMP8
MAP2K1
PDK1
PIM1
MELK
MMP2
WEE1
NR1D1
HDAC1
CDK2
BMP1
ACHE
PDE4A
PDE4B
PDE4D
PDE4C
MMP12
MMP25
HDAC6
MMP16
HDAC8
MMP14
ADAM10
CDC25A
CDC25B
PTGS1
ALOX15
EDNRA
HDAC3
HDAC5
HDAC10
TLR9
VCP
RAF1
PIM2
BRAF
GSK3A
MAPK14

	PIK3CD
	PIK3CB
	MCL1
	PIK3R1
	EPHB2
	ABL1
	PRKCA
	CDK1
	GSTP1
	GSTM2
	NOX4
	GRIA1
	ROCK2
	ANPEP
	NCOR2
	CCNC
	SLC1A3
	HDAC11
	CDK8
	SYK
	HPGDS
	NR4A1
	HDAC7
	HDAC9
	PLK1
	CDK4
	TRPM8
	CCND3
	KDM1A
MOL004838	BACE1
	HSD17B2
	HSD17B1
	BRAF
	GSK3B
	HSP90AA1
	OPRD1
	EIF2AK3
	LRRK2
MOL004841	MAOB
	CHRNA7
	AKR1B1
	BACE1
	PTGS2
	ALOX5

EGFR
F3
APP
ABCG2
CYP19A1
TERT
PTPN1
NOS2
SNCA
MAOA
ABCB1
CXCR4
TYR
ABCC1
SHBG
CBR1
HSD17B14
MMP2
ERN1
INSR
CLK1
RPS6KB1
TUBB1
IGF1R
PDK1
ODC1
GLI2
GLI1
BCL2L1
BCL2
CAPN1
WEE1
ESR1
ESR2
VCP
TYMS
NOX4
FLT3
PLK1
ESRRA
ESRRB
ABL1
PDGFRB
CDK4

	CHEK1
	ALPL
	DUSP3
	HDAC1
	MAP4K4
	MAPT
	ADCY5
	ALOX5AP
	HPGDS
	YWHAG
	PGD
	CYP1B1
	CDK1
	NQO2
	ROCK1
	HSD11B1
	PLAU
	RAF1
	CA3
	PIK3CB
	PIK3CA
	DNM1
	BRAF
	PTGS1
	PDPK1
	HDAC8
	DRD5
	DRD4
	SIGMAR1
	CHEK2
	ALDH2
	MTOR
	IGFBP3
	HDAC6
	SRC
	MMP12
MOL004848	ABCG2
	CYP19A1
	AKR1B1
	APP
	MAOB
	CHRNA7
	PDPK1
	NOS2

	PTPN1
	KCNA3
	ODC1
	F3
	ALOX5
	BACE1
	EGFR
	ABCB1
	PTGS2
	HSP90AB1
	PTGES
	TUBB1
	MAPKAPK2
	MAPKAPK5
	CXCR2
	PDK1
	HSP90B1
	EGLN1
	MAOA
	PTGS1
	PDE5A
	PDE10A
	BCL2L1
	GCGR
	ROCK2
	ALDH2
	CCND1
	PLAU
	MELK
	ALOX12
	FBP1
	PRKCD
	TERT
	TLR9
	ALK
	CHEK2
	WEE1
	EDNRA
	AKR1C3
MOL004849	PTPN1
	ACHE
	PDE4D
	CCR4
	PDK1

	HSP90B1
	HSP90AA1
	XPO1
	PLAU
	GSK3B
	HSP90AB1
	ALDH2
	CYP19A1
	NOS2
	PLG
	F2
	PRSS1
	F10
	SRD5A1
	ERBB2
	TLR9
	CCND1
MOL004855	PTPN1
	ACHE
	PIM1
	HSP90AA1
	PIM2
	PIM3
	ADRB2
	ADRB1
	PDK1
	CA4
	CBR1
	ACP1
	IL2
	MELK
	GCGR
	CDK2
	CDK1
	HSP90AB1
	ALDH2
	OPRK1
	SRC
MOL004856	PTPN1
	ACHE
	HSD17B2
	ABCB1
	CYP19A1
	CA12

IL2

ABCG2

ALDH2

HSD17B1

MGAM

ADORA1

ESRRA

ESRRB

CA7

CBR1

HSP90AB1

EGFR

HSP90AA1

SLC6A2

PTGS1

PDK1

CA4

PDE5A

MAOA

ADORA2A

CDK2

ALOX12

CDK4

NOS2

TYR

MIF

FBP1

ESR2

ESR1

PFKFB3

RELA

CNOT7

DUSP3

EGLN1

CALCA

HSP90B1

OPRK1

ALOX15

PRKCI

GABRB3

GABRA1

TBXAS1

PPARA

ERN1

	AGTR1
	GCGR
MOL004857	PTPN1
	ACHE
	CYP19A1
	HSD17B2
	CA7
	CA12
	CBR1
	ABCB1
	ALOX15
	MIF
	TYR
	HSP90AB1
	IL2
	CA4
	EGFR
	PPARA
	PTGS1
	HSD17B1
	ALDH2
	HSP90AA1
	IDH1
	FASN
	PTPRS
	TBXAS1
	MGAM
	HTR2A
	HTR2C
	ADORA1
	ESRRA
	ESRRB
	ABCG2
	CA2
	CA1
	PDK1
	PTGS2
	HSP90B1
	IKBKB
	PTGES
	ESR2
	ALOX12
	SLC6A2
	PLAU

	ESR1
	F10
	FBP1
	SIRT1
	KISS1R
	RELA
	CHEK2
	CHEK1
	DUSP3
	PRKCE
	PFKFB3
	PRSS1
MOL004863	PTPN1
	AKT1
	PDE5A
	ESR2
	MIF
	TYR
	ESR1
	NOX4
	BCL2
	ALOX12
	HSP90B1
	ALOX15
	XDH
	TBXAS1
	MGAM
	HTR2A
	HTR2C
	ESRRA
	ESRRB
	BACE1
	SIRT1
	HSP90AB1
	CCR4
	ABCB1
	HSP90AA1
	F10
	CA4
	ALDH2
	NOS2
	HSD17B1
	GCGR
	MCL1

	ACHE
	RELA
	CYP19A1
	PFKFB3
	PDE10A
	FASN
MOL004864	PTPN1
	ACHE
	HSD17B2
	CYP19A1
	ABCB1
	PDE5A
	RELA
	IL2
	HSP90AB1
	NOS2
	HSD17B1
	ABCG2
	ALDH2
	HSP90B1
	HSP90AA1
	PDK1
	CA12
	CDK2
	CDK1
	PTGS1
	CA4
	CA7
	MGAM
	HTR2A
	HTR2C
	ESRRA
	ESRRB
	ADORA1
	MCL1
	BCL2
	MELK
	ESR2
	FASN
	BAD
	SLC6A2
	EGFR
	FBP1
	ALOX15

	DUSP3
	AGTR1
	EP300
	PDE4D
	CBR1
	MPI
	CCND1
	ESR1
	CNOT7
	GCGR
MOL004866	AKT1
	PPARG
	CYP19A1
	ABCG2
	CDK5
	MAOA
	CCNB3
	ALOX5
	ADORA1
	GLO1
	SYK
	PARP1
	TTR
	MMP9
	MMP2
	MMP12
	CD38
	CYP1B1
	AKR1B10
	TNKS2
	TNKS
	TOP1
	ARG1
	PTPRS
	AKR1B1
	XDH
	CA2
	CA12
	ACHE
	ABCC1
	PDE5A
	ESR1
	ESR2
	CDK6

ADORA2A
HSD17B1
CSNK2A1
NAE1
AMY1A
GRK6
F2
MAPT
KDM4E
GPR35
AVPR2
TOP2A
IGF1R
INSR
PIM1
AURKB
DRD4
MYLK
MPO
PIK3R1
DAPK1
PYGL
PTK2
HSD17B2
KDR
MMP13
MMP3
CA3
PLK1
PIK3CG
PKN1
MET
NEK2
CA13
CAMK2B
NEK6
PLA2G1B
CA5A
APEX1
NUAK1
AKR1C2
AKR1C1
AKR1C3
AKR1C4

	AKR1A1
	ALOX15
	BCHE
	PTPN1
	BACE1
	TERT
	CA4
	PLG
	CA7
	AR
	ST6GAL1
	CBR1
	CDK2
	GSK3B
	CA1
	CA9
	APP
	PFKFB3
	PLA2G2A
	ABCB1
	TYR
	AHR
MOL004879	ACHE
	PTPN1
	PDE4D
	GCGR
	ALDH2
	MAOB
	HSD17B2
	CES1
	CES2
	SCD
	AURKA
	TGFBR1
	ACVR1
	MMP9
	MMP1
	MMP2
	HTR3A
	ROCK2
	ROCK1
	ODC1
	MMP13
	HDAC8

COMT
ADORA2B
S1PR3
S1PR1
AKR1B10
MET
HDAC6
HDAC2
PTAFR
KDR
HDAC1
HDAC9
MMP3
XPO1
HPGDS
CMA1
BMP4
TBXA2R
DUSP3
DPP4
MELK
FLT1
CHRNA7
SLC5A1
ADAMTS5
ADAMTS4
PLK1
MMP14
TRPV1
MIF
ERN1
MPI
PDE7A
PIK3CA
MTOR
CDC25A
BMP1
MMP7
CDC25B
KDM5C
KDM4B
KDM5B
PDK1
KDM4A

	SF3B3
	ERBB2
	HDAC3
	NCOR2
	HDAC5
	HDAC7
	NCOR1
	HDAC11
	HDAC4
	F3
	HDAC10
	CYP19A1
	CCNB3
	ADAM17
	CTSL
	CHEK1
	F10
	NOS2
	PLEC
	FLT3
	ME1
	MCHR1
	ABL1
	TNNC1
	PDE4A
	PDE4B
	SLC10A2
	PDE4C
	RPS6KB1
	AGPAT2
	HSD17B1
	BRD4
	TAS2R31
	CSNK1A1
MOL004882	ESR1
	PPARG
	LRRK2
	FLT1
	FGFR1
	AGTR1
	PIK3CA
	MMP1
	MMP7
	FCER2

	SLC5A2
	MAP3K12
	BACE2
	SLC5A1
	DUT
	BCHE
	ACHE
	HTR7
	HTR6
	HPSE
	CDK1
	CCNE1
	P2RX3
	FDFT1
	IRAK4
	ADRA2A
	MTOR
	MMP8
	DRD1
	LTB4R
MOL004883	PTPN1
	CYP19A1
	ACHE
	ESR1
	XDH
	TBXAS1
	MGAM
	HTR2A
	ESRRA
	ESRRB
	HSD17B1
	ALDH2
	AR
	ABCG2
	MAOA
	ABCB1
	EGFR
	HSP90AA1
	HSD17B2
	HSP90AB1
	GCGR
	BCL2L1
	NOX4
	RELA

	PTPRS
	IL2
	ADORA2A
	CA12
	TYR
	MIF
	ADRB2
	ADRB1
	CHEK2
	PDE10A
MOL004884	PTPN1
	HSP90AA1
	ADRB2
	ADRB1
	CA7
MOL004885	ODC1
	MAOB
	PTPN1
	RET
	FLT1
	PIK3R1
	EPHB2
	ALPG
	ALPL
	PLAA
	GRM4
	MYLK
	GSK3A
	PIM2
	MARK1
	MMP2
	KDM1A
	HSD17B2
	VCP
	FLT3
	TUBB1
	PLK1
	CCND3
	ADAM17
	MMP7
	MMP8
	CHEK1
	WEE1
	MAPK14

PDE7A
PARP1
FGFR2
PTK6
ESR1
ABL1
CCNB3
ADORA2A
MTOR
PIK3CD
PRKDC
PIK3CB
HCK
PIK3CG
MAP2K1
EPHB4
PDPK1
ADORA2B
HSD17B3
PI4KB
DNM1
RAF1
BRAF
PDGFRB
CXCR2
HSD11B1
ERN1
CCND1
CCNE2
CHRNA7
AURKB
CSNK1G1
RPS6KA1
ROCK1
MAPK1
MMP14
PIM1
ESR2
EP300
DBF4
TRPM8
ERBB2
EZR
ABAT

	CDK1
	CCNE1
	CDK3
	AURKA
	JAK3
	PDE4A
	LCK
	PDE4B
	PDE4C
	ATM
	RPS6KA2
	ALK
	ATR
	NCOR2
	MMP16
	HDAC11
	HDAC10
	CTSD
	CDC25A
	BMP1
	CDC25B
	ANPEP
	TGM2
	SPHK2
	CXCR1
	SPHK1
	LRRK2
MOL004891	PTPN1
	ESR1
	ESR2
	MIF
	ALDH2
	SREBF2
	COMT
	HSD17B3
	PLK1
	MAPK1
	ERBB2
	EGFR
	CCNB3
	PDE7A
	WEE1
	GRM4
	RET

LCK
MAPK14
CHEK1
PDGFRA
CCND3
PFKFB3
MCL1
DPP4
LTA4H
ELANE
PDE5A
CCND1
CCNE2
ADORA2B
CTSS
FLT3
GCGR
TLR9
CDK5
CSNK1D
AURKA
GPBAR1
PLAA
NOX4
NOX1
ADORA2A
VCP
SPHK2
SPHK1
ERN1
HRH4
HSD17B2
PIM1
CASP3
PIM2
PIM3
MAPT
MTOR
SNCA
GABRB3
GABRA1
GABRG2
GABRA6
AURKB

	CHRNA7
	PDE4B
	EP300
	PTK6
	HPGDS
	HDAC3
	HDAC6
	HDAC2
	TNNC1
	ADAMTS5
	HDAC1
	NPY5R
	TRPV1
	BRAF
	TRPM8
	FYN
	SCN4A
	TTR
	CMA1
	SCN9A
	AKR1C4
	AKR1C2
	AR
	DRD1
	DRD2
	DRD3
	SIGMAR1
	PIK3CG
	BMP1
	AGPAT2
	HPGD
	PDE4D
	S1PR3
	S1PR1
	JAK3
	GSK3B
	ROCK2
	HSP90AA1
	HSD11B1
MOL004898	AKR1B1
	BACE1
	EGFR
	TERT
	CYP19A1

NOS2
SNCA
CHRNA7
APP
ABCG2
PTGES
ABCB1
F3
TUBB1
PDE10A
ALOX12
ALDH2
PDPK1
EGLN1
PTGS2
KCNA3
HSP90B1
HSP90AA1
BCL2L1
GCGR
MAOB
MAOA
HSP90AB1
ALK
MAPT
PDK1
XDH
AKR1C3
ALOX5
PTGS1
ESR1
CCND1
ESR2
NOX4
PDE5A
HSD17B2
HSD17B1
FBP1
GSK3B
ODC1
PTPN1
WEE1
RAF1
ROCK2

	DNASE1L3
	CHEK1
	CXCR4
	AR
	MCL1
	KISS1R
	F10
	CTSD
	CFTR
	CXCR2
	MMP8
MOL004904	MAOB
	HDAC3
	MTOR
	MAP2K1
	PDE10A
	CHRM1
	FKBP1A
	PIK3CG
	PIK3CA
	CDC42BPA
	WDR5
	WNT3A
	TNKS2
	ERN1
	SLC5A2
	HDAC11
	HDAC10
	DNM1
	ROCK2
	ROCK1
	SYK
	ALOX5AP
	PCNA
	CSF1R
	DHFR
	ADORA3
	HSP90AA1
MOL004905	HMGCR
	ACHE
	GSK3B
	NR3C2
	HSD11B1
	NOS2

PGR
TNF
PRSS1
CTRC
AR
F2
PTGS2
CYP19A1
EPAS1
PLA2G2A
POLB
PTPN1
NR3C1
HSD11B2
S1PR3
DNTT
NR1H4
EPHX2
IDH1
PDE2A
PDE10A
CYP17A1
CES2
APP
HSD17B2
AVPR2
AVPR1A
MAPK8
SHBG
AKR1C2
AKR1C1
P2RX3
CCNT1
NPY5R
SERPINA6
SLC10A2
CCKBR
PDPK1
KIF11
SLC6A2
TACR2
SLC6A3
FASN
MMP3

	MTOR
	PIK3CA
	FNTA
	CPT1A
	SLC16A1
	CXCR3
	LIMK2
	ACKR3
	SYK
	MCHR1
	PRKCA
	P2RY1
	TACR1
	PRKD1
	CTSL
	KCNA5
MOL004907	PPARA
	ABCB1
	CYP19A1
	CA7
	HSD17B2
	CA12
	CA4
	CBR1
	TYR
	MIF
	PTPRS
	TLR9
	CA2
	CDC7
	MAOB
	EGFR
	LCK
	NOX4
	XDH
	ALOX12
	ESR1
	ESR2
	ADORA1
	ADORA2A
	SIRT1
	DUSP3
	CDC25B
	BCHE

	ACHE
	TBXAS1
	MAOA
	MGAM
	HTR2A
	HTR2C
	HSD17B1
	ESRRA
	ESRRB
	ABCG2
MOL004908	PTPN1
	ODC1
	MMP1
	GSK3A
	SREBF2
	FLT3
	ERN1
	CHEK1
	ALDH2
	SRC
	GCGR
	TLR9
	CHRNA7
	AURKA
	ESR2
	TYR
	MET
	MAOB
	KDR
	MERTK
	CYP11B1
	ELANE
	CYP11B2
	MMP13
	MMP3
	ADAM17
	MMP14
	MMP7
	MMP12
	MMP8
	EGFR
	BMP4
	TBXA2R
	HDAC3

HDAC6
EPHX2
ADORA2A
ADORA2B
HDAC8
HDAC1
CDK5
MPEG1
S1PR3
S1PR1
PDK1
GRIN1
HSD17B2
HSD17B1
CYP17A1
HSP90AA1
TRPV1
SCD
AVPR1A
PFKFB3
TNNC1
STAT6
ESR1
COMT
CCNB3
ADORA1
DRD4
KIF11
PLK1
AURKB
HDAC5
MAPK14
HDAC7
HDAC10
HDAC2
MAPK10
CDC25A
MAPK1
HSD17B3
CDC25B
GRK6
PLEC
CHEK2
CMA1

	TGFBR1
	ACVR1
	RAF1
	BRAF
	PLA2G1B
	NPY5R
	IDH1
	BMP1
	BCL2
	VCP
	ADAMTS5
	ADAMTS4
	STK17B
	PRF1
	SLC29A1
	MELK
	SQLE
	IGF1R
	DPP4
	ROCK2
	ALOX5AP
	HPGD
MOL004910	ESR2
	ESR1
	CYP19A1
	PPARG
	ABCG2
	HSD17B1
	SHBG
	CES2
	ACHE
	MAOB
	CES1
	ADORA1
	BCHE
	RXRA
	CA12
	CA4
	PTGS1
	ADORA3
	BACE1
	TAS2R31
	CA7
	PLA2G1B

CTSL
CYP1B1
SLC5A2
SERPINE1
PLA2G2A
HSD17B2
MMP13
CBR1
AKR1C3
PDE4B
PTPN1
MTNR1A
MTNR1B
ABCC1
MYLK
EGLN1
POLB
GCGR
CDK2
CSF1R
MMP1
PLA2G7
CDC25B
PLA2G5
ERN1
MMP12
MAP3K5
PSEN2
ODC1
FYN
CYP11B1
EPHA2
YES1
BLK
LCK
SYK
CSK
CYP11B2
EPHB2
GSTA1
CYP17A1
BMX
LYN
EPHA5

	EPHA4
	TXK
	FGR
	EPHA6
	PTK6
	EPHB3
	EPHA3
	BTK
	TYRO3
	COQ8B
	EPHA1
	PIM1
	STAT6
	TACR3
	HSP90AA1
	QPCT
	DYRK1B
	CDK5
	CCNB3
	HSD17B3
	PDE10A
	PIM2
	PIM3
	MET
	RAF1
	SLC29A1
	PARP1
	FAP
	FADS1
	CXCR2
	FTO
	ADORA2A
	NOS1
	KDM4E
MOL004911	ESRRA
	ESRRB
	ESR1
	ESR2
	HSD17B2
	HSD17B1
	RAF1
	EPHB4
	MAPK14
	JAK2

EGFR
ELANE
KIF11
HSP90AA1
PTGS1
CTSD
PDE7A
AKR1B1
AKR1B10
NAAA
DRD1
JAK3
DRD3
RARG
RARA
ALOX15
TRPM8
DRD5
CCND1
CCNE2
DRD4
F3
TNKS2
TNKS
GCGR
SLC5A2
BACE1
CCNE1
CCNE1
PTGS2
F10
AVPR1A
HTR1B
HTR1A
SLC6A2
ADORA1
TACR2
ADORA3
LCK
TSPO
PTGIR
PRKCZ
FASN
HDAC6

	HDAC2
	HDAC8
	MMP9
	MMP1
	MMP2
	ADAM17
	CAPN1
	CHEK1
	BRAF
	WEE1
MOL004912	PTPN1
	HSP90AB1
	CA7
	ADRB2
	ADRB1
	PDE10A
	HSP90AA1
	ALDH2
	PDE4D
	GCGR
MOL004913	ALOX5
	CBR1
	ESR2
	ESR1
	CA5B
	CA1
	CA12
	CA9
	CA7
	KCNMA1
	KCNA5
	KCNA3
	EGFR
	SRC
	GSR
	CYP1A2
	CA4
	AKR1B1
	CA6
	CA14
	CA13
	CA5A
	HSD17B1
	ERBB2

	CCND1
	PDGFRB
	FLT4
	INSR
	CDK2
	PLK4
	TEK
	AURKA
	MAP3K8
	BRAF
	EPHB4
	SQLE
	FGR
	LYN
MOL004914	ALOX5
	CBR1
	ESR2
	ESR1
	CA13
	CA14
	CA5A
	MAOA
	KCNA5
	KCNA3
	CA5B
	GSR
	AKR1B1
	ERBB2
	CCND1
	PDGFRB
	FLT4
	INSR
	CDK2
	GSK3B
	CSNK2A1
	PLK4
	TEK
	AURKA
	MAP3K8
	BRAF
	EPHB4
	SQLE
	FGR
	LYN

	EGFR
	CA12
	CA9
MOL004915	PTPN1
	ACHE
	CYP19A1
	ESR1
	HSD17B1
	TBXAS1
	MGAM
	HTR2A
	ESRRA
	ESRRB
	ALDH2
	ABCB1
	ABCG2
	AR
	XDH
	HSD17B2
	HSP90AA1
	MAOA
	EGFR
	IL2
	ADRB2
	ADRB1
	CA12
	GCGR
	HSP90AB1
	PDE10A
	CHEK2
	TYR
	MIF
	NOX4
	PFKFB3
	BCL2L1
	RELA
	PTPRS
MOL004917	ALDH2
	IL2
	TNF
MOL004924	EIF4A1
	SLC5A2
	SLC29A1
	EGFR

	ADORA2A
	TYR
	CYP19A1
	ADORA1
	CA14
	ADK
	SLC28A2
	SLC5A1
	SLC5A4
	ABL1
	PDGFRA
	ADORA2B
	CASP3
	GBA
	MGMT
	MAP2K1
	PYGL
	HSPA5
	CTSL
	SLC2A1
	CTSK
	CTSS
MOL004935	ABCG2
	CYP19A1
	ESR1
	ESR2
	ACHE
	CA7
	CA12
	CA4
	CYP1B1
	BCHE
	CES1
	PPARG
	CES2
	PLA2G1B
	HSD17B1
	PTGS1
	RXRA
	MAOB
	TAS2R31
	ADORA1
	ADORA3
	SHBG

MMP13
MMP12
ABCC1
CBR1
CTSL
BACE1
ODC1
KLK2
BRAF
SLC5A2
HSD17B2
PTPN1
CA2
CA1
DRD1
DRD3
NOX4
ESRRA
ESRRB
GCGR
MCL1
IGF1R
INSR
CHRNA7
SRC
AKR1B10
MMP1
DRD5
DRD4
F3
PDK1
SGK1
BCL2
DNM1
CHEK1
WEE1
HPGDS
ABCB1
BCL2L1
DYRK1B
ADAM17
MMP2
POLB
RET

	EPHB4
	SERPINE1
	PLA2G5
	ERN1
	PLG
	ADORA2B
	MAP4K4
	CA6
	CA13
	CA5B
	CA5A
	TNF
	CLK1
	APP
	RAF1
	MMP9
	MMP3
	MMP8
	HSP90AA1
	FLT3
	PLK1
	MAPK14
	TOP1
	ADORA2A
MOL004941	CYP19A1
	ESR1
	ESR2
	MAOB
	CA7
	CA12
	HSD17B1
	ABCG2
	CA4
	SHBG
	CYP1B1
	ABCC1
	CBR1
	ADORA1
	ADORA3
	TAS2R31
	PTGS1
	AKR1C3
	PLA2G1B
	CES1

GRM5
MMP13
MMP12
POLB
PLA2G5
PLA2G10
CES2
BACE1
SERPINE1
RXRA
GRM2
PPARG
SRC
KLK1
KLK2
PLA2G2A
EDNRA
BCHE
ABL1
CHEK1
WEE1
SLC5A2
ERN1
RPS6KA5
ALK
CA2
CA1
ACHE
MET
PIK3CG
ALPG
HSD17B3
PTGER1
PTGER4
PTGER3
GSK3B
APP
CISD1
ALPL
LNPEP
CA3
CA6
CA13
CA5B

	CA5A
	DAO
	EEF2K
	CDK1
	CA9
	AURKB
	CDK2
	CDK4
	AURKA
	GCGR
	CDK5
	CCNE1
	CCNB2
	BRAF
	DPP4
	HNF4A
	RPS6KB1
	IGF1R
	PGF
	VEGFA
	GRK2
	GRM4
	MAP3K8
	ABCB1
MOL004945	ESR1
	ESR2
	ABCG2
	CYP19A1
	HSD17B1
	ACHE
	CES2
	SHBG
	CA7
	CA4
	BCHE
	PPARG
	BACE1
	CES1
	MAOB
	PTGS1
	CA12
	CBR1
	PLA2G1B
	SLC5A2

ADORA1
ADORA3
HSD17B2
MMP12
ABCC1
CYP1B1
TAS2R31
CTSL
RXRA
MMP13
CTSD
POLB
PLA2G5
KDM1A
ODC1
TYMS
PTPN1
ADAM17
BRAF
GCGR
LCK
PDE7A
EPHB4
MMP8
KLK1
KLK2
MMP3
PRKCZ
ABL1
AR
PLA2G2A
MMP16
MMP15
MMP26
OPRD1
CLK1
DYRK1B
CA1
CA6
CA5B
CA5A
ERBB2
AGTR1
RAF1

	HSP90AA1
	WEE1
	MTOR
	SERPINE1
	NR1H4
	NAAA
	PDK1
	DNM1
	ADRA2A
	ADRA2C
	ADRA2B
	PTGS2
	SRC
	RET
	CMA1
	ALOX5
	GSK3B
	CHEK1
	TRPV1
	NEK1
	MET
	NOX4
	TKT
	TNKS2
	TNKS
	OPRM1
	OPRK1
	KCNA5
	CDK2
	CDK3
	AKR1B10
	CA2
	PLAU
	SYK
	PLA2G7
	MDM4
MOL004948	PDE4D
	HDAC3
	PTPN1
	CA3
	ODC1
	APP
	KCNA3
	HDAC2

	CHRNA7
	HDAC5
	HDAC11
	HDAC4
	FLT3
	HPGDS
	MET
	CA2
	CA1
	EGFR
	KDR
	MMP13
	MMP9
	MMP1
	MMP2
	CHEK1
	ERN1
	IMPDH2
	P2RX3
	CCNB3
	HDAC7
	HDAC9
	SCN9A
	MMP8
	HDAC10
	PDE4A
	PDE4B
	PDE4C
	HTR3A
	CES1
	PPARG
	CES2
	HSD17B1
	COMT
	SRC
MOL004949	CYP19A1
	XDH
	PTPN1
	ABCG2
	BACE1
	ABCB1
	ACHE
	HSD17B2
	HSD17B1

TYR
AHR
ESRRA
ESR1
CA2
AKR1B1
FLT3
ADORA3
PDE5A
CDK1
DAPK1
MPG
MAOA
CA12
ALOX15
MAPT
KDM4E
TOP2A
IGF1R
INSR
AURKB
DRD4
GLO1
MYLK
MPO
PIK3R1
PYGL
SYK
PTK2
KDR
MMP13
MMP3
CA3
PLK1
CSNK2A1
NEK2
CA13
NEK6
PLA2G1B
CA5A
APEX1
NUAK1
AKR1C2
AKR1C1

AKR1C3
AKR1C4
AKR1A1
ADORA2A
EGFR
CA7
AKT1
AR
F2
PIM1
CDK2
SLC22A12
PFKFB3
GSK3B
PKN1
ALOX12
CAMK2B
ALK
AXL
ESR2
CCNB3
HSP90AB1
ARG1
CA4
CYP1B1
ALOX5
NOX4
HSP90AA1
CA1
CA9
AVPR2
ABCC1
BCHE
GCGR
TTR
AKR1B10
TNKS2
TNKS
SRC
NAE1
AMY1A
CFTR
BCL2L1
BCL2

	CDK5
	CDK6
	TERT
MOL004957	ESR1
	HSD17B2
	CA12
	ALDH2
	IL2
	EGFR
	CYP19A1
	CA7
	ESR2
	HSD17B1
	PTGS1
	ABCG2
	CA4
	PFKFB3
	MIF
	HTR2A
	ADORA1
	ADORA2A
	MAOA
	MAOB
	SLC6A2
	CA2
	ABCB1
	CA1
	TBXAS1
	ALOX12
	CBR1
	HTR2C
	ESRRA
	ESRRB
	PTPRS
	MGAM
	ALOX15
	PPARA
	XDH
	TYR
	NOX4
	PON1
	CA9
	MAP2K1
	ACHE

PTPN1
FLT1
RET
CHRNA7
EPHB2
DRD1
CA14
GRIA1
CHEK1
WEE1
CA3
TLR9
EZR
BACE1
ADAM17
HDAC1
HDAC6
ALPL
CDC25A
CDC25B
MMP1
F3
RPS6KA2
KDM1A
CA6
CA13
CA5B
CA5A
GSK3A
PIM2
MARK1
TRPM8
SLC9A1
ROCK1
ABL1
CDK2
HCK
PI4KB
EPHB4
PLAA
NCOR2
HDAC11
PTK6
HDAC10

MOL004959	MMP8
	DYRK1A
	GRM4
	MMP7
	RPS6KB1
	AURKA
	CCND3
	ALPG
	BMP1
	PDK1
	RAF1
	PDPK1
	BRAF
	CDK4
	MMP25
	PTPN1
	AURKB
	RPS6KB1
	AURKA
	TYR
	EPHB4
	CYP19A1
	BRAF
	DRD1
	SIGMAR1
	ESR1
	ESR2
	HSD17B2
	AKR1B10
	RAF1
	PTGS1
	ABL1
	EPHA2
	LCK
	EPHB2
	EPHA5
	EPHA4
	EPHA8
	EPHA7
	EPHB3
	EPHA3
	EPHB1
	EPHA1
	PLK1

AR
SGK1
HSP90AA1
TRPM8
SLC29A1
PDPK1
CTSK
ADAM17
CCNE1
CFD
CAPN1
TNF
SPHK2
PIK3CA
SPHK1
CSF1R
PIM1
CLK1
PIM2
P2RX7
DYRK1B
MAPKAPK2
CSNK1G1
RPS6KA1
ROCK1
MAPK1
STAT6
CTSD
MMP1
THRA
THRB
CCNE1
BACE1
IMPDH1
IMPDH2
AGTR1
OPRK1
HTR7
GCGR
MAPK8
HDAC5
HDAC7
HDAC4
HSP90AB1

	PDK1
	MMP3
	MMP8
	CHEK1
	TKT
	WEE1
	PIK3CD
	MKNK2
	P2RX3
	KDM1A
	DNM1
	OPRL1
	TLR9
	CDK1
	CA14
	GRK2
	MMP10
	TNNI3K
	SYK
	TGM2
	HSD11B1
	ALK
	MAOA
	CAPN1
	CCNC
	PARP1
	PITRM1
MOL004961	AKR1B1
	XDH
	CA2
	CA7
	CA12
	CA4
	CYP1B1
	APP
	NOX4
	PLG
	MCL1
	BACE1
	ABCC1
	ABCG2
	ABCB1
	CYP19A1
	HSD17B2

F2
EGFR
IGF1R
ADORA1
OPRD1
ACHE
ALOX15
ALOX12
HSD17B1
CDK1
MET
ESR2
PIK3CG
MAOA
ADORA2A
OPRM1
AVPR2
FLT3
PIM1
ALOX5
AURKB
DRD4
GLO1
MPO
PIK3R1
DAPK1
PYGL
CA1
GSK3B
SRC
PTK2
KDR
MMP13
MMP3
CA3
PLK1
CA6
MMP9
MMP2
PKN1
CA14
CA9
CSNK2A1
NEK2

	CXCR1
	CAMK2B
	ALK
	AKT1
	NEK6
	PLA2G1B
	CA5A
	AXL
	NUAK1
	AKR1C2
	AKR1C1
	AKR1C3
	AKR1C4
	CA13
	AKR1A1
	MAPT
	KDM4E
	GPR35
	TOP2A
	INSR
	MYLK
	SYK
	APEX1
	PLA2G2A
	CDK5
	CCNB3
	CDK6
	CDK2
	ARG1
	TERT
	NOS2
	SLC22A12
	ST6GAL1
	KIT
	ESR1
	ADORA3
	TYR
	AHR
	ESRRA
MOL004966	PTPN1
	KDR
	SRC
	FGFR1
	KIT

ODC1
MET
RAF1
BRAF
CDK2
CDK4
RET
GRM4
PLK1
ADORA1
PDK1
ABL1
PIM1
MTOR
PIK3CD
PRKDC
PIK3CB
HCK
PIK3CG
PI4KB
PIM2
CHEK1
EPHB4
CCNB3
LCK
ALPG
PLAA
ADORA2A
ADORA2B
PRF1
WEE1
TRPM8
ESR1
ESR2
ERN1
PIM3
KDM1A
PTK6
ALPL
ADAMTS5
FLT3
MYLK
ADORA3
GSK3A

MARK1
CRHR1
DRD1
HTR1A
DRD3
ELANE
CDK1
HSD17B2
HSD17B1
HDAC1
SMARCA2
TERT
GCGR
PIK3R1
EPHB2
MAOB
RORC
HDAC6
HDAC8
PARP1
MPEG1
HSD17B3
TBXA2R
AURKB
PTK2
TGFBR2
ATR
NEK1
ROCK1
PRKACA
DNM1
ENPP1
THRA
GABRA1
GABRA1
PDE5A
XIAP
HSD11B1
NOX4
NOX1
EGLN1
CCR9
HTR2C
CDC25A

	BMP1
	CDC25B
	TRPV1
	VCP
	PPIA
	PTPsigma
	FPR2
MOL004974	PTPN1
	KDR
	MET
	SRC
	FGFR1
	KIT
	ODC1
	PIM1
	PIM2
	CDK2
	CDK4
	PIM3
	PLK1
	LCK
	RAF1
	CCNB3
	BRAF
	RET
	PIK3R1
	EPHB2
	CHEK1
	WEE1
	AURKB
	PTK2
	HSD17B2
	HSD17B1
	VCP
	GRIA1
	MTOR
	ROCK1
	HSD17B3
	ABL1
	ADORA1
	ADORA2B
	PRKCA
	CDK1
	LTA4H

CCND3
ESR1
ESR2
TRPM8
PIK3CG
RAC1
PDE5A
PTK6
HDAC8
HDAC1
ADORA3
DNM1
CDC25C
CDC25A
PRKACA
CDC25B
PRKDC
ATM
ATR
ADORA2A
ENPP1
ERN1
TRPV1
NOX4
NOX1
CCND1
FLT3
TBXA2R
CCNE2
PTPsigma
GCK
MELK
ALPL
EP300
CDK5
RPS6KA3
GRM4
CSNK1D
PIK3CD
IRAK4
S1PR3
S1PR1
PLK3
PDPK1

	JAK2
	SREBF2
	MMP14
	RORC
	AR
	LNPEP
	SHBG
	MPEG1
	XIAP
	PDK1
	ALPG
	CMA1
	CTSG
	PDE10A
	PLAA
	CXCR2
	PDE4A
	PDE4B
	PDE4C
MOL004978	PTPN1
	DRD1
	HTR1A
	DRD2
	FGFR1
	CHEK1
	WEE1
	DRD3
	ESR1
	ESR2
	ABL1
	FLT3
	MMP1
	PDK1
	KDR
	CCND1
	CCNE2
	CCNB3
	MAPT
	PIM1
	SNCA
	RORC
	TBXA2R
	THRA
	SIGMAR1

ODC1
EP300
MET
LCK
ESRRG
GCGR
EGFR
PCNA
ADORA2A
ADORA2B
HSD17B3
LNPEP
S1PR3
S1PR1
ELANE
SRC
ERN1
GRIA1
PARP1
CDK5
PDE3A
HSD17B2
PDE7A
PLAA
MAOB
NR4A1
MMP13
MMP3
HPGDS
CRHR1
HSD17B1
RET
AURKB
PLK1
KDM1A
MELK
KIF11
CTSD
F3
PDE10A
TRPV1
UQCRB
HDAC6
FLT1

	AR
	NR3C1
	PIK3R1
	HDAC8
	EPHB2
	ALPL
	TRPM8
	PBRM1
	PDE5A
	CHRNA7
	ROCK2
	SMARCA4
	SHBG
	GABRB2
	GABRA1
	PDE4A
	ADORA3
	MAPK14
	MTOR
	PIK3CD
	PIK3CG
	PKM
	ATP4B
	ADORA1
	FASN
	LTA4H
	NEK1
	VCP
	ERBB2
	AURKA
	P2RX7
MOL004980	CA7
	CA1
	CA9
	AKR1B1
	MMP1
	ESR2
	PRKCZ
	CA12
	CA13
	HSD17B3
	TYMS
	HSP90AA1
	DRD2

RAF1
DYRK1A
DHFR
MAPK8
PAK4
CHEK1
CAPN1
STAT6
GCGR
CLK1
DYRK1B
HTR2B
NR3C1
CASR
DRD1
DUSP3
MDM4
MDM2
RPS6KB1
HSP90AB1
MMP14
ESR1
JAK2
NEK1
ADAM17
PDK1
PIK3CD
CDK4
TNNI3K
CHRNA4
CDK2
CDK3
PITRM1
WEE1
ADRA1A
DRD3
SIGMAR1
CYP19A1
AGTR1
TGFBR1
EGLN1
PDGFRA
OPRM1
OPRK1

	PIM2
	COMT
	LNPEP
	LDLR
	ABL1
	DCTPP1
	KIF11
	ALPL
	MAP2K2
	MMP7
	PDE10A
	RPS6KA3
	FCER2
	MMP8
	CCNB1
	HDAC5
	HDAC7
	PLA2G2A
	HDAC4
	GRK2
	CTSD
	MAP2K7
	HDAC6
	MAPK14
	HTR7
	HDAC8
	MAPK11
	P2RX7
	HSD11B1
	ALOX15
	ALOX12
	CYSLTR1
	ADRA2A
	ADRA2C
	ADRA2B
	JAK3
	CHRM1
	SLC6A2
	ADRA1D
	SLC6A4
	TACR2
	CHRM3
	JAK1
MOL004988	PTPN1

ESR2
ESR1
LDLR
ADORA1
ADORA2A
GRK3
GRK2
GRK5
NR1H4
KIF11
ADAMTS5
EGFR
CXCR2
CCNA2
MGAM
QPCTL
ADORA2B
GRM2
F10
ELANE
PDE10A
GSK3B
ITK
GCK
HSP90AA1
SLC2A1
SLC2A3
SLC2A2
CCKAR
CTSD
CCKBR
NOS1
NOS3
CDK5
ABL1
CTSB
GPR55
ADORA3
SLC10A2
TTK
CXCR1
ADAMTS4
GRM5
GRM1

	NPY5R
	CPT1A
	JAK3
	JAK1
	JAK2
	CHEK1
	PARP1
	MAPK1
	ODC1
	MAPK8
	GPR119
	CDK2
	SIGMAR1
	EP300
	SLC8A1
	HSP90AB1
	AURKA
	PDK1
	RET
	HDAC1
	STK17B
	STK17A
	HCRTR2
	HCRTR1
	DDX3X
MOL004989	ABCG2
	ESR2
	ESR1
	CYP19A1
	PPARG
	ACHE
	PLA2G1B
	MMP13
	CA7
	CA12
	MAOB
	CES1
	HSD17B1
	BCHE
	CA4
	CYP1B1
	CES2
	MMP12
	BACE1

SHBG
PTPN1
ADORA1
TAS2R31
ADORA3
PTGS1
ABCC1
CTSL
SLC5A2
KLK1
KLK2
MYLK
GSK3B
GSK3A
CDK2
PIM2
MARK1
RXRA
KDM1A
MMP1
ADAM17
MTOR
PIK3CG
PIK3CA
GUSB
CA2
CA6
CA13
CA5B
CA5A
CA3
MET
HSD11B1
ODC1
CA1
POLB
CBR1
MMP9
CHRNA7
PLA2G5
HSD17B2
MMP2
PIM1
TERT

	ALDH2
	ABCB1
	MMP3
	MMP7
	SPHK2
	SPHK1
	BRAF
	ALPL
	CSNK1G1
	RPS6KA1
	ROCK1
	MAPK1
	ALPG
	TNF
	RAF1
	CCNB3
	HSP90AA1
	BMP1
	CDK4
	RET
	GCGR
	HSD17B3
	FGFR1
	CHEK1
	WEE1
	MMP8
	IMPDH2
	CLK1
	DYRK1B
	MAPK14
	KDR
	PITRM1
	FASN
	PTK6
	CA9
	HSP90AB1
	DNM1
MOL004990	CA7
	CA12
	CA4
	HSD17B2
	MAOB
	MIF
	CA2

CYP19A1
CBR1
ALDH2
IL2
EGFR
ABCB1
TLR9
XDH
ACHE
CA1
HSD17B1
ESRRA
ABCG2
ESR1
ESR2
TBXAS1
MAOA
MGAM
HTR2A
HTR2C
ADORA1
ADORA2A
ESRRB
PON1
ALOX12
PTGS1
SLC6A2
BACE1
TYR
IGF1R
IGFBP3
PLAT
F2
F10
PLAU
SNCA
CDC7
PTPRS
CDK2
ARG1
NOX4
MCL1
ALOX15
PFKFB3

IGFBP6
IGFBP4
IGFBP5
IGFBP2
PARP1
IGFBP1
CHEK1
WEE1
GLO1
MMP9
MMP2
MMP12
CD38
TOP1
AVPR2
MPO
PIK3R1
DAPK1
PYGL
SRC
PTK2
KDR
MMP13
MMP3
PLK1
CDK1
PKN1
MET
NEK2
CXCR1
CAMK2B
ALK
AKT1
NEK6
PLA2G1B
AXL
NUAK1
AKR1C2
AKR1C4
AKR1A1
MPG
SLC22A12
HSP90AB1
ALOX5

MOL004991	PTGS1
	ALDH2
	SLC6A2
	IL2
	CA12
	CYP19A1
	CA7
	ADORA1
	ADORA2A
	HTR2A
	HTR2C
	CA4
	MAOB
	MAOA
	ESR2
	CBR1
	TBXAS1
	EGFR
	ABCG2
	CA2
	CA1
	HSD17B2
	PDE10A
	PFKFB3
	KDM5C
	KDM4A
	KDM4D
	KDM4C
	DBF4
	IRAK4
	PIK3CD
	PIK3CB
	PIK3CA
	KCNH2
	CSF1R
	PRKCQ
	TGFBR1
	MGAM
	ESRRB
	LRRK2
	GRM5
	GRM1
	NAAA
	EPHX1

SIRT2
GCK
PROKR1
TRPM8
AGPAT2
SCD
CRHR1
PIK3CD
EPHX2
SHH
CDK1
CDK9
TYMS
DHFR
JAK3
ADORA2B
JAK2
CYP17A1
ABCB1
NTRK1
TRPA1
GRIN2A
GABRB3
GABRA2
MAPK8
ADAMTS5
GRIA2
CCND1
GABRB3
GABRA5
CCNE2
KCNK3
KCNK9
AURKA
PRF1
PDGFRA
MAPKAPK2
DYRK1A
MAPK10
HDAC1
QPCT
PTGES
TGFB1
CDC7

	FLT1
	NR3C2
	NR3C1
	NR1H4
	PGR
	KAT2B
	ESR1
	PSEN2
	DHODH
	PGK1
	CDK9
	NPY5R
MOL004993	ABCG2
	ESR1
	ESR2
	CYP19A1
	CA12
	ACHE
	CA7
	CA4
	CYP1B1
	HSD17B1
	PTPN1
	PTGS1
	MAOB
	ABCC1
	SHBG
	CBR1
	BACE1
	CES1
	MMP12
	MMP13
	BCHE
	PPARG
	CES2
	ADORA1
	TAS2R31
	ADORA3
	SLC5A2
	ODC1
	PLA2G1B
	CA2
	CA1
	KLK2

HSD17B14
BCL2L1
CTSL
BCL2
NOX4
SRC
WEE1
BRAF
CLK1
DYRK1B
CDK1
CCNE1
CDK3
GRK2
AURKA
AR
HSD17B2
SERPINE1
CHRNA7
CA3
CA6
CA13
CA5B
CA5A
IGF1R
RXRA
INSR
NQO2
VCP
MMP3
ESRRA
ESRRB
DYRK1A
MMP1
YWHAG
HDAC2
RPS6KB1
POLB
SGK1
PLA2G5
PLA2G10
MTOR
ADAM17
DNM1

	GCGR
	RAF1
	ERN1
	DUSP3
	DRD1
	DRD3
	MMP2
	F3
	CHEK1
	CA9
	EPHB4
	MMP9
	AKR1B10
	CALM1
	ADORA2B
	PIK3CB
	CYP2C9
	CYP3A4
	CA14
	PIK3CA
	DRD5
	DRD4
	GSK3B
	CDK4
MOL005000	ACHE
	ALDH2
	PTPN1
	CA7
	CA4
	CBR1
	IL2
	ESR2
	SLC6A2
	EGFR
	TBXAS1
	MAOA
	ESR1
	MGAM
	HTR2A
	HTR2C
	ADORA1
	ESRRA
	ESRRB
	PPARG

	PDE4D
	F10
	NFKB1
	ADORA2A
	ERBB2
	PTPRS
	RELA
MOL005001	PTPN1
	RARA
	MELK
	HSP90AA1
	MTOR
	PIK3CA
	F2
	F10
	PRKCA
	PRKCB
	PRKCE
	PDK1
	KISS1R
	PRKCD
	ACHE
	AGTR1
	GCGR
	VCP
	AKR1B1
	CHEK2
	MPG
	BCL2L1
	CHEK1
	LDHA
	LDHB
MOL005003	PTPN1
	CDK4
	P2RX7
	TYR
	STAT6
	EPHB4
	AURKB
	AURKA
	BRAF
	TRPM8
	SLC29A1
	CDK2

TUBB1
HSP90AB1
RPS6KB1
FLT3
MAPK8
AKR1B10
TNF
PARP1
HSD11B1
CFD
HSD17B2
HSP90AA1
CDK1
CCNE2
SPHK2
CDK1
SPHK1
ESR1
PDE10A
CHEK1
WEE1
OPRK1
MMP1
TGFBR1
ROS1
FLT4
PDGFRA
EZR
MMP3
ADAM17
MMP7
ABL1
CDK5
EPHA2
CCNB3
LCK
P2RX3
EPHB2
EPHA5
EPHA4
EPHA8
EPHA7
PDK1
EPHB3

EPHA3

EPHB1

EPHA1

DNM1

TKT

MDM2

AR

MAPKAPK2

CSNK1G1

RPS6KA1

ROCK1

MAPK1

OXTR

PDE5A

GCGR

DRD1

PIM1

MMP10

PIM2

PSEN2

PITRM1

DUSP3

HDAC1

FAAH

MGLL

ESR2

RAF1

SYK

GSK3B

SIGMAR1

KIF11

MAOA

CCNC

CDK8

AVPR2

AVPR1A

F3 F7

BACE2

ADAM33

CYP19A1

CCNE1

TYMS

PDPK1

CCNE1

MOL005007	ODC1
	MAOB
	MYLK
	GSK3A
	PIM2
	MARK1
	KDM1A
	ERN1
	STAT3
	PDGFRB
	MMP2
	HSD11B1
	GRM4
	MAPK14
	RET
	PLK1
	ADAM17
	PTPN1
	ADORA2B
	HSD17B2
	HSD17B3
	ALPL
	RAF1
	BRAF
	CCNB3
	ALDH2
	PIM1
	AR
	CHRNA7
	PLAA
	TRPM8
	CCNE1
	FLT3
	PDPK1
	JAK2
	CDK3
	AURKA
	ACACB
	EZR
	ROCK2
	PTK6
	SAE1
	PFKFB3
	MMP14

BMP1

MMP8

CHEK1

WEE1

CDK1

DRD1

DRD2

DRD3

SIGMAR1

TUBB1

ADORA2A

SYK

PDE5A

PDE10A

CTSK

BRD4

ALPG

CDC25A

CDC25B

TGM2

MTOR

PIK3CG

PDK1

ESR1

CDK2

CDK9

SCD

RPS6KA2

MAOA

ERBB2

HDAC10

SREBF2

DRD4

EP300

CCNE2

TGFBR1

ACVR1

ABL1

CCND1

LIPG

TRPV1

ANPEP

ADAMTS5

ADAMTS4

	TYMS
	MME
	FNTA
	ROCK1
	GCK
	PRKACA
	ENPP1
	HTR1A
	PIK3CD
	PRKDC
	PIK3CB
	HCK
MOL005008	CYP19A1
	XDH
	HSP90AB1
	BACE1
	HIF1A
	HSP90AA1
	GCGR
	RPS6KA3
	ADRB2
	ADRB1
	SIRT1
	WEE1
	TYR
	AHR
	ESRRA
	RAF1
	CHEK2
	CHEK1
MOL005012	ALDH2
	BMP4
	WEE1
	SCD
	ERBB2
	AKR1B10
	PDE4A
	PDE4B
	PDE4C
	MET
	GCGR
	HSD11B1
	CDK2
	PDK1

HPGDS
COMT
HSD17B2
NOX1
CES1
CES2
ERN1
TGFBR1
ACVR1
MMP13
AKT2
PTGES
ODC1
PDGFRB
KIT
AURKB
KDR
CDK2
CDK4
MMP1
NAMPT
MMP9
MMP2
ADAMTS5
ADAMTS4
MMP14
ROCK2
DNM1
TAS2R31
F10
ALPL
MAPT
APP
MAPK14
STK17B
SNCA
ACHE
IMPDH2
NR1H4
ADAM17
GSK3B
GSK3A
CDC25A
CDC25B

	PDGFRA
	RET
	LCK
	TRPV1
	SREBF2
	THRA
	ME1
	BRAF
	PLAA
	IRAK4
	ROCK1
	HSD17B3
	AURKA
	EGFR
	CSF1R
	SLC5A1
	YES1
	CHEK1
	EPHB4
	HSD17B1
	PARP1
	MELK
	ACACB
	CHRNA7
MOL005013	FNTA
	NOS2
	AR
	PRKCH
	HSD11B2
	PTPN11
	HSD11B1
	AKR1B10
	TNF
	BACE1
	PTPN1
	POLB
	PTGES
	SRD5A2
	ESR1
	ESR2
	CYP19A1
	NR3C1
	PGR
	PTPN2

NPC1L1
SERPINA6
SIGMAR1
SHBG
CYP17A1
PTGER4
PTGER2
ALOX5
LTB4R
BCHE
IDO1
RORA
MAPK3
CES2
FDFT1
PTGER1
CDC25A
PTGER3
SLC6A3
ADORA3
PTGDR
PPARA
PPARD
PTGFR
PPARG
HMGCR
GPBAR1
PTGIR
FABP1
NR3C2
MMP3
MMP1
MMP2
NR1H3
CES1
PREP
THRA
THRB
SLC22A6
AGTR1
NR1I2
HSD17B3
SCD
NR1I3

	G6PD
	CYP51A1
	PTPN6
	ALOX5AP
	OAT
	ITGAL
	FABP4
	FABP3
	FABP5
	PTGDR2
	TOP2A
	TOP1
	CCR1
	PLA2G1B
	CDC25B
	SLC6A4
	PIK3CA
	FAAH
	RORC
	PTPRF
	ACP1
	TRPM8
	HDAC6
	HDAC1
	CASP1
	F11
	ITGB1
	IMPDH1
	IMPDH2
	ITGA2B
MOL005016	EGFR
	HSD17B2
	CA7
	CA12
	CA4
	ABCB1
	IL2
	CYP19A1
	CBR1
	ESR1
	ESR2
	HSD17B1
	XDH
	PTPRS

ALOX12
TLR9
TYR
CA2
MIF
NOX4
PTGS1
SLC6A2
ADORA1
MAOA
ADORA2A
ABCG2
PPARA
ALOX15
ESRRA
CA1
ALDH2
CHEK1
WEE1
ACHE
TBXAS1
MGAM
HTR2A
HTR2C
ESRRB
PFKFB3
PON1
HSP90B1
BACE1
GCGR
HSP90AB1
MAOB
TNNC1
AKR1B1
KIT
OPRD1
IGFBP6
IGFBP4
IGFBP5
IGFBP2
IGFBP3
IGFBP1
HSP90AA1
PTPN1

	ALPL
	PLAU
	MCL1
	DUSP3
	CDC25B
	PDE10A
	STS
MOL005017	SRD5A1
	ESR2
	XPO1
	CA7
	CA13
	CCR4
	ESR1
	GPR84
	HSP90B1
	HSP90AA1
	GCGR
	PTPN1
	FNTA FNTB
	ALPL
	RELA
	PDE5A
	HSP90AB1
MOL005018	CTSK
	ODC1
MOL005020	RPS6KB1
	ESR2
	PTPN1
	SYK
	RAF1
	BRAF
	SLC5A1
	HSD17B2
	HSD17B1
	PDK1
	TAS2R31
	CYP2C9
	CYP3A4
	CYP2C19
	PLA2G1B
	GNRHR
	EP300
	TRPV1

BACE1
ABL1
HDAC6
EGFR
HDAC1
CHEK1
WEE1
CTSD
TGFBR1
SLC29A1
ADORA1
ADORA2A
ADORA3
BCHE
ESR1
ESRRA
ESRRB
RARG
RARA
MAPK10
HSP90AA1
HSD11B1
MMP13
PLK1
MMP1
ADAM17
TAAR1
TKT
ODC1
ERBB2
PTGIR
RET
HTR1A
THRA
THRB
OPRM1
NAAA
PDE10A
GCGR
S1PR3
S1PR1
TYR
PIM1
SLC5A2

		PIM2
		STAT6
		PIM3
		NR1H4
		PIK3CD
		PIK3CG
		KDR
Hong Hua	MOL000006	NOX4
		AKR1B1
		CDK5
		XDH
		MAOA
		FLT3
		CA2
		CCNB3
		ALOX5
		ADORA1
		CA7
		GLO1
		APP
		SYK
		GSK3B
		PARP1
		TTR
		MMP9
		CA12
		MMP2
		CA4
		MMP12
		CD38
		CYP1B1
		ABCG2
		AKR1B10
		TNKS2
		TNKS
		TOP1
		ARG1
		PTPRS
		ABCC1
		HSD17B1
		ACHE
		CDK6
		ABCB1
		HSD17B2

CYP19A1
ESR2
ADORA2A
CSNK2A1
ALOX15
ALOX12
ESR1
PTGS2
CFTR
AMY1A
GRK6
CA1
CA9
CDK2
TERT
CDK1
TYR
AHR
ESRRA
GPR35
AVPR2
IGF1R
EGFR
F2
PIM1
AURKB
DRD4
MPO
PIK3R1
DAPK1
PYGL
SRC
PTK2
KDR
MMP13
MMP3
CA3
PLK1
CA6
PKN1
CA14
MET
NEK2
CXCR1

	CAMK2B
	ALK
	AKT1
	NEK6
	PLA2G1B
	CA5A
	BACE1
	AXL
	NUAK1
	AKR1C2
	AKR1C1
	AKR1C3
	AKR1C4
	CA13
	AKR1A1
	PFKFB3
	PLG
	KDM4E
	AR
MOL000098	NOX4
	AVPR2
	AKR1B1
	XDH
	MAOA
	IGF1R
	FLT3
	CYP19A1
	EGFR
	F2
	CA2
	PIM1
	ALOX5
	AURKB
	DRD4
	ADORA1
	CA7
	GLO1
	MPO
	PIK3R1
	ADORA2A
	DAPK1
	PYGL
	CA1
	GSK3B

SRC
PTK2
HSD17B2
KDR
MMP13
MMP3
CA3
ALOX15
ABCC1
PLK1
CA6
CDK1
MMP9
CA12
MMP2
PKN1
CA14
CA9
CSNK2A1
ALOX12
MET
CA4
NEK2
CXCR1
CAMK2B
ALK
AKT1
ABCB1
NEK6
PLA2G1B
CA5A
BACE1
CYP1B1
AXL
ABCG2
NUAK1
AKR1C2
AKR1C1
AKR1C3
AKR1C4
CA13
AKR1A1
GPR35
MAPT

	KDM4E
	TOP2A
	INSR
	ACHE
	MYLK
	SYK
	PIK3CG
	APEX1
	PTPRS
	ESR2
	MPG
	SLC22A12
	CDK5
	CCNB3
	ARG1
	CDK6
	CDK2
	TYR
	HSD17B1
	AHR
	ESRRA
	APP
	PARP1
	TTR
	MMP12
	CD38
	AKR1B10
	TNKS2
	TNKS
	TOP1
	TERT
MOL000422	NOX4
	AKR1B1
	XDH
	TYR
	FLT3
	CA2
	ALOX5
	CA7
	HSD17B2
	ABCC1
	HSD17B1
	AHR
	CA12

ESRRA
ABCB1
CYP1B1
ABCG2
ADORA1
CA4
ACHE
MAOA
GLO1
SYK
GSK3B
MMP9
MMP2
ALOX15
ALOX12
PTPRS
ADORA2A
CDK5
CCNB3
ARG1
GPR35
ESR2
DAPK1
MPG
SLC22A12
TTR
AKR1B10
TNKS2
TNKS
CDK6
CDK2
CYP19A1
CSNK2A1
EGFR
AVPR2
IGF1R
F2
PIM1
AURKB
DRD4
MPO
PIK3R1
PYGL
CA1

	SRC
	PTK2
	KDR
	MMP13
	MMP3
	CA3
	PLK1
	CA6
	CDK1
	PKN1
	CA14
	CA9
	MET
	NEK2
	CXCR1
	CAMK2B
	ALK
	AKT1
	NEK6
	PLA2G1B
	CA5A
	BACE1
	AXL
	NUAK1
	AKR1C2
	AKR1C1
	AKR1C3
	AKR1C4
	CA13
	AKR1A1
	APP
	PARP1
	MMP12
	CD38
	TOP1
	ESR1
	PTGS2
	CFTR
	PFKFB3
	AMY1A
	GRK6
	TERT
	MAPT
MOL002694	CHRM2

CHRM1
TAAR1
PDE10A
FAP
EZR
ABCB1
PTGS2
ABCC1
PREP
MET
SCN9A
FKBP1A
HTR2A
PIM1
PDE3A
MAPK10
PDE3B
GRM5
PIM2
NTRK1
HCRTR2
BRD4
NAMPT
PDE7A
P2RX7
CREBBP
PTGER4
PTGER2
EGFR
ADORA2A
SYK
JAK3
MAPK8
JAK2
CCNE1
DAPK3
CDK9
HSD11B1
ADORA1
CASP3
ADORA2B
GSK3B
MMP13
PARP1

CASP7
AKT1
SCD
ABL1
FLT1
MAPKAPK2
SRC
KDR
HCRTR1
ESR2
ADORA3
PIK3CD
PIK3CB
PIK3CG
PIK3CA
ABCG2
AURKB
MAPK14
CDK2
CA12
IKBKE
CA9
ADAM17
NOS2
BRSK2
TBK1
MARK3
CES1
EPHX2
SCN10A
PARP10
MMP2
TNKS2
TNKS
AVPR2
PDE5A
PDGFRB
FLT3
HTR2C
CHRM3
GRM4
HRH4
RPS6KA2
BCL2A1

	ERBB2
	PSEN2
	HRH3
	CTSK
	CTSS
	PDE9A
	CTSL
	HSP90AA1
	CMA1
	PDE1C
	PARP3
MOL002695	NR3C1
	PDE5A
	SYK
	CYP2C9
	CYP2C19
	TRPV1
	MTOR
	PIK3CA
	RORC
	PDE7A
	CYP3A4
	CHRM2
	BRD2
	CFD
	CDK2
	ABL1
	ADORA3
	ICAM1
	PIK3CD
	PRKDC
	PIK3CB
	HCK
	PIK3CG
	PI4KB
	SELE
	BRAF
	NAMPT
	PFKFB3
	TAAR1
	GABRA2
	MAPK8
	PLK1
	NTRK1

VCAM1
TTK
CDK5
AURKB
PTK2
CMA1
SCN10A
JAK3
PIM2
FKBP5
JAK1
JAK2
ERBB4
KNG1
TUBB1
TGM2
TRHR
MAPK3
CAPN1
TGFBR1
FNTA
FPR1
FPR2
NR5A1
PDE10A
BRD4
IKBKB
DYRK1A
MAPK10
PDE2A
BRD9
DYRK1B
PDPK1
GSK3B
MMP9
CDK4
MMP1
RPS6KB1
MMP8
BACE2
IKBKE
TBK1
RAF1
F10

	PGK1
	IRAK4
	HCRTR1
	LRRK2
	MKNK1
	CDC7
	MAP3K7
	MAP2K1
	CCND1
	SSTR3
	CCNE2
	CCNB3
	NOS2
	ALPL
	PDGFRB
	PPP1CA
	RPS6KA3
	ATM
	AGPAT2
	SMO
	CSF1R
	F9
	GSK3A
MOL002712	AKR1B1
	NOX4
	XDH
	FLT3
	CA2
	ALOX5
	CA7
	HSD17B2
	ABCC1
	CA12
	ABCB1
	CYP1B1
	ABCG2
	PFKFB3
	ADORA1
	ADORA2A
	CA4
	PIM1
	PTPRS
	GPR35
	DAPK1

ESR2
MPG
SLC22A12
TYR
HSD17B1
AHR
ESRRA
AVPR2
MAOA
IGF1R
CYP19A1
EGFR
F2
AURKB
DRD4
GLO1
MPO
PIK3R1
PYGL
CA1
GSK3B
SRC
PTK2
KDR
MMP13
MMP3
CA3
ALOX15
PLK1
CA6
CDK1
MMP9
MMP2
PKN1
CA14
CA9
CSNK2A1
ALOX12
MET
NEK2
CXCR1
CAMK2B
ALK
AKT1

	NEK6
	PLA2G1B
	CA5A
	BACE1
	AXL
	NUAK1
	AKR1C2
	AKR1C1
	AKR1C3
	AKR1C4
	CA13
	AKR1A1
	AMY1A
	GRK6
	ACHE
	TERT
	MAPT
	KDM4E
	TOP2A
	INSR
	MYLK
	SYK
	PIK3CG
	APEX1
	CDK5
	CCNB3
	ARG1
	CDK6
	CDK2
	TTR
	AKR1B10
	TNKS2
	TNKS
	ESR1
	APP
MOL002714	KDM4E
	XDH
	ALOX15
	CDK1
	ALOX12
	GRK6
	CYP19A1
	CA7
	CA12

CA4
ABCB1
CYP1B1
HSD17B1
AKR1B1
CDK5
CA2
CCNB3
CDK6
CA1
CA9
ABCG2
CBR1
ESR2
ESR1
ACHE
ADORA1
ADORA2A
PTGS2
PTPRS
AMY1A
FLT3
IKBKB
NTRK2
AR
NOX4
MAOA
SYK
GSK3B
ABCC1
TTR
CSNK2A1
CFTR
AKR1B10
TNKS2
TNKS
HSD17B2
BCHE
ADORA3
TERT
LCK
PFKFB3
PIM1
PARP1

ALOX5
APP
CALM1
ARG1
NOS2
GLO1
MMP9
MMP2
MMP12
CD38
TOP1
NAE1
EGFR
SRC
TYR
AHR
ESRRA
KIT
OPRD1
CYP1A1
AURKB
PIK3CG
MAPT
TOP2A
INSR
MYLK
APEX1
IGF1R
KDR
PLK1
MET
ALK
AXL
PTPN1
ST6GAL1
SLC22A12
GPR35
FYN
TACR2
PRKDC
MAPK3
BACE1
SIRT1
PLA2G2A

	PLA2G4A
	CA6
	CDK2
MOL002717	IGFBP3
	AKR1C3
	NOX4
	CAMKK2
	LCK
	SELL
	SELE
	SELP
	MMP16
	MMP13
	MMP1
	MMP2
	MMP14
	MMP8
	AKR1B1
	ECE1
	ESR2
	CDC25B
	DHODH
	IGFBP5
	AMPD3
	MME
	NR4A1
	HSP90AA1
	HSP90AB1
	PIM1
	KDM4C
	GRK6
	VCP
	ERN1
	MET
	NEU4
	MKNK2
	TYR
	PYGL
	PYGM
	MCL1
	GPR35
	ITGB1
	PFKFB3
	F2

	EGLN1
	BCL2A1
MOL002719	CYP19A1
	CA7
	CA12
	CA4
	CYP1B1
	ESR1
	MAOB
	PTGS1
	HSD17B1
	TAS2R31
	ESR2
	ABCC1
	SHBG
	CBR1
	ABCG2
	ADORA1
	ADORA3
	AKR1C3
	PLA2G1B
	MMP12
	GRM5
	CES1
	PPARG
	CES2
	MMP13
	KLK1
	KLK2
	BACE1
	SLC5A2
	CHRNA7
	SRC
	POLB
	PLA2G2A
	PLA2G5
	PLA2G10
	STS
	DYRK1A
	MAPT
	MAPK14
	PGD
	BCL2
	APP

	EDNRA
	TERT
	CA2
	CA1
	BCHE
	AKT1
	CDK5
	CA6
	CA13
	CA5B
	CA5A
	RXRA
	CA9
	ABCB1
	MET
	KIT
	KDR
	FGFR1
	NOX4
	GRM2
	ALOX12
	HSD17B2
	SERPINE1
	NQO2
	SNCA
	DNMT1
	MMP2
	MMP14
	STAT1
	SQLE
	IGF1R
	INSR
	FFAR1
	CTSB
MOL002757	CHEK1
	CYP11B2
	HSP90AA1
	FBP1
	PARP14
	PARP15
	PARP10
	CCND1
	HTR2C
	PARP1

		GSK3B
		SIRT2
		ASAH1
		CISD1
		PIK3CD
		PIK3CB
		PIK3CG
		RPS6KB1
		DYRK1B
		MTNR1B
		CASP3
		CASP7
		FAP
		CASP1
		SRC
		CCNC
		ICAM1
		VCAM1
		SELE
		PTK2B
		PDE3A
		RPS6KA5
		CLK3
		MTNR1A
		IDH1
		GSK3A
		ALOX15
	MOL002773	RBP4
Jie Geng	MOL001689	CYP1B1
		CYP19A1
		CA7
		CA12
		CA4
		CBR1
		ABCC1
		ESR2
		ABCB1
		NOX4
		TNKS2
		TNKS
		CDK5
		XDH
		CCNB2
		ACHE

CDK6
ESR1
AKR1B1
FLT3
HSD17B1
ABCG2
MAOA
ADORA1
ADORA2A
SYK
GSK3B
TTR
AKR1B10
KIT
OPRD1
LCK
HSD17B2
ALOX15
ALOX12
PTGS2
CSNK2A1
CFTR
PIM1
CA2
MCL1
PTPRS
PLG
ARG1
ALOX5
CA1
CA9
GRK6
APP
GLO1
PARP1
MMP9
MMP2
MMP12
CD38
TOP1
AMY1A
PLA2G2A
NAE1
CDK1

		KDM4E
		TYR
		AHR
		ESRRA
		TERT
		ADORA3
		MAOB
		PFKFB3
		SLC22A12
		AR
		ST6GAL1
		SIGMAR1
		IGF1R
		EGFR
		NOS2
		BACE1
		CALM1
		CYP1A1
		CYP1A2
		F2
		DAPK1
		CDK2
		GPR35
		MPG
		AVPR2
		AURKB
		DRD4
		MPO
		PIK3R1
		PYGL
		SRC
		PTK2
		KDR
		MMP13
		MMP3
		CA3
		PLK1
		CA6
		PKN1
		CA14
Niu Xi	MOL000098	NOX4
		AVPR2
		AKR1B1
		XDH

MAOA
IGF1R
FLT3
CYP19A1
EGFR
F2
CA2
PIM1
ALOX5
AURKB
DRD4
ADORA1
CA7
GLO1
MPO
PIK3R1
ADORA2A
DAPK1
PYGL
CA1
GSK3B
SRC
PTK2
HSD17B2
KDR
MMP13
MMP3
CA3
ALOX15
ABCC1
PLK1
CA6
CDK1
MMP9
CA12
MMP2
PKN1
CA14
CA9
CSNK2A1
ALOX12
MET
CA4
NEK2

CXCR1
CAMK2B
ALK
AKT1
ABCB1
NEK6
PLA2G1B
CA5A
BACE1
CYP1B1
AXL
ABCG2
NUAK1
AKR1C2
AKR1C1
AKR1C3
AKR1C4
CA13
AKR1A1
GPR35
MAPT
KDM4E
TOP2A
INSR
ACHE
MYLK
SYK
PIK3CG
APEX1
PTPRS
ESR2
MPG
SLC22A12
CDK5
CCNB3
ARG1
CDK6
CDK2
TYR
HSD17B1
AHR
ESRRA
APP
PARP1

	TTR
	MMP12
	CD38
	AKR1B10
	TNKS2
	TNKS
	TOP1
	TERT
MOL000173	PTGS2
	NOS2
	FLT3
	AKR1B1
	OPRD1
	KIT
	ABCB1
	IKBKB
	NTRK2
	KDM4E
	XDH
	ALOX15
	CDK1
	ALOX12
	GRK6
	CYP19A1
	ESR2
	CYP1A1
	OPRM1
	ABCG2
	ESR1
	EGFR
	SLC22A12
	CYP1B1
	CA4
	CA2
	CA1
	MCL1
	PIK3CG
	PIM1
	ADORA1
	ADORA2A
	HSD17B2
	HSD17B1
	CA7
	CA12

CDK5

CCNB3

CDK6

CA9

CBR1

TERT

AR

CA6

PTPRS

DAPK1

MPG

PFKFB3

MMP9

MMP2

LCK

MMP12

CD38

TOP1

ARG1

MAPT

TOP2A

INSR

DRD4

MYLK

MPO

PIK3R1

PYGL

MMP13

MMP3

CA3

CA14

MET

CA13

CAMK2B

PLA2G1B

CA5A

APEX1

AKR1C2

AKR1C1

AKR1C3

AKR1C4

AKR1A1

GPR35

ODC1

	HSP90AA1
	ALOX5
	CXCR1
	PLA2G2A
	SRC
	APP
	CYP1A2
	ADORA3
	HSP90B1
	ABCC1
	TNKS
	TTR
	NOX4
	AVPR2
	NAE1
	BCHE
	ACHE
	BACE1
	PDE5A
	GSK3B
MOL000422	NOX4
	AKR1B1
	XDH
	TYR
	FLT3
	CA2
	ALOX5
	CA7
	HSD17B2
	ABCC1
	HSD17B1
	AHR
	CA12
	ESRRA
	ABCB1
	CYP1B1
	ABCG2
	ADORA1
	CA4
	ACHE
	MAOA
	GLO1
	SYK
	GSK3B

MMP9
MMP2
ALOX15
ALOX12
PTPRS
ADORA2A
CDK5
CCNB3
ARG1
GPR35
ESR2
DAPK1
MPG
SLC22A12
TTR
AKR1B10
TNKS2
TNKS
CDK6
CDK2
CYP19A1
CSNK2A1
EGFR
AVPR2
IGF1R
F2
PIM1
AURKB
DRD4
MPO
PIK3R1
PYGL
CA1
SRC
PTK2
KDR
MMP13
MMP3
CA3
PLK1
CA6
CDK1
PKN1
CA14

	CA9
	MET
	NEK2
	CXCR1
	CAMK2B
	ALK
	AKT1
	NEK6
	PLA2G1B
	CA5A
	BACE1
	AXL
	NUAK1
	AKR1C2
	AKR1C1
	AKR1C3
	AKR1C4
	CA13
	AKR1A1
	APP
	PARP1
	MMP12
	CD38
	TOP1
	ESR1
	PTGS2
	CFTR
	PFKFB3
	AMY1A
	GRK6
	TERT
	MAPT
MOL000785	ACHE
	HTR2B
	BCHE
	ADRA2C
	ADRA2B
	CHRM1
	SIGMAR1
	CYP2D6
	SAE1
	RAC1
	CDC42
	MAP2K1

AURKB
AURKA
CDK2
PLK1
TTK
PLK3
PLK2
PGK1
TRPC6
MAPKAPK2
CCNC
CDK8
HPGD
DYRK1A
GRM5
HTR3A
CYP1A1
NQO1
NQO2
CYP1B1
DRD4
PRF1
EPHX2
NTRK1
MTOR
MARK1
PIK3CD
PIK3CB
PIK3CG
KDM5B
ERBB2
GRM1
ABCG2
DHFR
CLK4
NAAA
PPIA
LCK
MET
LYN
EPHB4
TBXAS1
TEK
CDC25B

SCD
KIT
PIM1
CYP11B2
SLC1A3
CLK1
DYRK2
MDM2
CCND1
GCK
ALDH2
MAPK8
MAPK10
MCHR1
HCRTR2
TRPV1
CSF1R
CFD
ROCK2
RAF1
IKBKB
HSD17B2
F3
FPR2
LRRK2
RPS6KA2
NAMPT
MST1R
CSNK1D
MMP3
CDK2
MMP9
CDK4
MMP1
SIRT2
FLT4
RET
ITK
HSD17B1
STAT3
HSD11B1
PTGER2
PDGFRB
PFKFB3

MOL001458	ACHE
	SIGMAR1
	CHRM1
	HTR2B
	BCHE
	ADRA2C
	ADRA2B
	CYP2D6
	SAE1
	RAC1
	CDC42
	CHRM4
	TBXAS1
	XBP1
	IKBKB
	PLK1
	PRF1
	ABL1
	CHEK2
	SCN9A
	CDC7
	LIMK1
	SCD
MOL002714	KDM4E
	XDH
	ALOX15
	CDK1
	ALOX12
	GRK6
	CYP19A1
	CA7
	CA12
	CA4
	ABCB1
	CYP1B1
	HSD17B1
	AKR1B1
	CDK5
	CA2
	CCNB3
	CDK6
	CA1
	CA9
	ABCG2

CBR1
ESR2
ESR1
ACHE
ADORA1
ADORA2A
PTGS2
PTPRS
AMY1A
FLT3
IKBKB
NTRK2
AR
NOX4
MAOA
SYK
GSK3B
ABCC1
TTR
CSNK2A1
CFTR
AKR1B10
TNKS2
TNKS
HSD17B2
BCHE
ADORA3
TERT
LCK
PFKFB3
PIM1
PARP1
ALOX5
APP
CALM1
ARG1
NOS2
GLO1
MMP9
MMP2
MMP12
CD38
TOP1
NAE1

	EGFR
	SRC
	TYR
	AHR
	ESRRA
	KIT
	OPRD1
	CYP1A1
	AURKB
	PIK3CG
	MAPT
	TOP2A
	INSR
	MYLK
	APEX1
	IGF1R
	KDR
	PLK1
	MET
	ALK
	AXL
	PTPN1
	ST6GAL1
	SLC22A12
	GPR35
	FYN
	TACR2
	PRKDC
	MAPK3
	BACE1
	SIRT1
	PLA2G2A
	PLA2G4A
	CA6
	CDK2
MOL002897	SAE1
	ACHE
	BCHE
	SIGMAR1
	HTR2B
	ADRA2C
	ADRA2B
	CHRM1
	CYP2D6

RAC1
RPS6KB1
AURKA
CDC42
HPGD
TBXAS1
MAOB
PIK3CG
GRK5
CYP19A1
SCD
AURKB
XBP1
NTRK1
PIK3CD
PIK3CB
GRIA1
MAPKAPK2
JAK2
IMPDH2
CHEK2
TGM2
CD38
LCK
PIM1
PIM2
MAPK10
MAP4K4
PARP1
F3
TRPM8
ABL1
SIRT3
SIRT2
SIRT1
NR3C2
DHFR
FLT1
ALOX5AP
ROCK1
BCAT2
PRKACA
CYP11B2
ICAM1

SELE
AGPAT2
DRD4
DRD3
KIT
SRC
MKNK1
FLT3
IKBKB
ADORA2A
ADORA3
PTPN1
ZAP70
SNCA
JAK3
GSK3A
IRAK4
MAPK1
SLC22A12
HTR3A
AKR1B1
TYMS
CHEK1
ERBB2
MAPK8
SCN9A
NPY5R
SLC5A1
PDE4B
CCNE1
CYP11B1
EPHA2
FAAH
MST1R
DPP4
ROCK2
CCNC
MET
STAT3
AXL
CDK8
MME
RPS6KA3
EPHX2

	QPCT
	MARS
	MMP9
MOL003847	CA1
	CA12
	CA9
	CA7
	CTSV
	CTSL
	KCNJ5
	KCNJ6
	GSK3B
	ADORA2A
	NQO2
	PREP
	FAP
	THRB
	MAPK14
	TGFBR1
	GPR139
	EGFR
	SRC
	KDR
	PDE10A
	MTNR1B
	ADORA2B
	PIK3CD
	PIK3CB
	PIK3CG
	TNKS2
	PPIA
	KDM5A
	KDM5B
	KAT2B
	CRHR1
	HCRTR2
	LIPG
	HCRTR1
	EZR
	DRD2
	DRD4
	MAPK8
	MDM2
	KCNA5

KCNA3
IDH1
CSF1R
LCK
GRM5
ALK
PSMB5
CASP3
SYK
CASP7
P2RX7
EPHX2
MET
HCAR2
PIK3CA
MKNK2
HSD11B1
NOS2
MKNK1
EZH2
CAPN1
CTSB
PTPRC
GRM1
TLR9
FNTA
PGGT1B
MMP3
MMP9
MMP1
AKR1C3
CHRNA7
P2RX3
GABRA2
S1PR3
NPY5R
F10
CDC25B
CYP11B1
CYP19A1
APP
CYP11B2
TRPA1
HTR2A

	HTR2C
	NAAA
	TSPO
	IMPDH2
	JAK3
	HTR7
	DDAH1
	NR3C1
	PSEN2
	TAOK1
	PDE4B
	HSD17B2
	JAK1
	HSD17B1
	PDE9A
MOL012542	MTOR
	SLC2A1
	CNR1
	EDNRB
	EDNRA
	PDE10A
	SLC28A2
	SYK
	PIK3CD
	PIK3CB
	PIK3CG
	PIK3CA
	AGTR2
	IKBKB
	PRKD1
	REN
	PRUNE1
	PTPN1
	METAP2
	PIK3C2A
	PIK3C3
	MAP3K9
	SLC5A2
	FGR
	SLC5A1
	PIK3C2B
	PDE5A
	FKBP1A
	MMP13

		MMP9
		MMP2
		MMP8
		F2
		F10
		LTB4R
		PRKDC
		PPIA
		KLK3
		CTSD
		CAPN1
		CTSB
		PDK1
		SLC29A1
		BACE2
		PDE4A
		PDE4B
		PDE4D
		HSP90AA1
		CASP1
		KCNH2
		CCR1
Sheng Dihuang	Coniferin	ADORA2A
		ADORA1
		ADORA3
		SLC5A2
	Rehmaglutin C	ADORA1
		ADORA2A
		ADORA3
		MCL1
		HSPA8
		HSPA5
		ADK
		DPP4
		ADA
		EIF4E
		GAPDH
		PNP
		HPRT1
		AHCY
		CDC42
		RAC1
		POLA1
		CDA

	TYMP
	CD38
	CA2
	CA1
	CA12
	CA9
	GBA
	GAA
	TK1
	OGA
	TERT
	FUCA1
	GRK1
	CCND1
	MAPK1
	EHMT1
	EHMT2
	CA7
	CA13
	CA14
	EGFR
	SETD7
	IDO1
	SRM
	HK2
	HK1
	TYR
	PDCD4
	F2
	DAO
Salidroside	TYR
	CA2
	CA7
	CA1
	CA3
	CA12
	CA14
	CA9
	CA4
	CA5A
	CA6
	ADK
	ADA
	ADORA2A

		AKR1C3
		ADORA3
		PNP
Tao Ren	MOL000296	CYP51A1
		HMGCR
		AR
		NPC1L1
		RORC
		CYP17A1
		NR1H3
		SREBF2
		ESR2
		CYP19A1
		ESR1
		SHBG
		SLC6A2
		PTPN1
		BCHE
		CHRM2
		SERPINA6
		RORA
		SLC6A4
		ACHE
		CYP2C19
		VDR
		NR1H2
		CDC25A
		GLRA1
		G6PD
		HSD11B1
		DHCR7
		PPARD
		SQLE
		CES2
		NR1I3
		CHRM4
		PTPN6
		PTPN2
		CCR1
		NR3C1
		NOS2
		TACR1
		HSD11B2
		PTGES

	POLB
MOL001328	NR3C2
	HMGCR
	TNF
	HSD11B1
	CYP17A1
	LTB4R
	CYP19A1
	PRSS1
	CTRC
	AMPD2
	NOS2
	EPAS1
	CPA1
	GSK3B
	NR3C1
	PLA2G2A
	ACE
	LTA4H
	PSEN2
	GSR
	AKR1B1
	EGFR
	SRC
	AMPD3
	ACHE
	PTPN1
	HSD11B2
	PTGS2
	TACR2
	AMPD1
	POLA1
	PTGDR2
	MAPK8
	SLC22A12
	MME
	PYGL
	PYGM
	ECE1
	PTGS1
	CTNNB1
	IL1B
	MMP3
	IMPDH1

	ITGAL
	CTSA
MOL001329	HMGCR
	NR3C2
	CYP17A1
	PLA2G2A
	AMPD2
	PRSS1
	CTRC
	AKR1B1
	ACE
	CYP19A1
	MME
	EPAS1
	LTB4R
	IMPDH2
	SLC22A12
	MMP3
	NR3C1
	GSR
	PGGT1B
	EGFR
	SRC
	ACHE
	IMPDH1
	PTGS2
	GSK3B
	AMPD3
	CTNNB1
	CPA1
	LTA4H
	CTSA
	PGR
	ANPEP
	ECE1
	PRKCA
	HSD11B2
	HSD11B1
	IL1B
	TACR2
	PTGS1
	ABCB1
	PARP1
	F2

	ACLY
	TNF
	PIM1
	PIM2
	PTPN1
	FOLH1
	CSNK2A1
MOL001339	HSD11B1
	PRKCA
	CPA1
	LTB4R
	AMPD3
	TLR9
	AMPD2
	AMPD1
	F2
	PRSS1
	PSEN2
	CTRC
	CES2
	SLC2A1
	ADCY1
	TRPV4
	LTA4H
	IDO1
	PTGDR2
	MAPK8
	GSR
	ITGAL
	ECE1
	CTSA
	ACE
	MME
	PTGS2
	CNR1
	IMPDH1
	ITGB1
	TP53
	EGFR
	SRC
	AKR1B1
	PYGL
	PYGM
	FLT1

	KDR
	GLRA2
MOL001340	FNTA
	CYP19A1
	IL1B
	HMGCR
	CDC25C
	CDC25A
	CDC25B
	PDE4D
	PTPN1
	PTPN2
	F2
	PRSS1
	CTRC
	AR
	NR3C2
	NR3C1
	HSD11B1
	PGR
	PLA2G2A
	PPP2CA
	CES2
	IKBKB
	BCL2L1
	PRKCD
	PPARG
	CXCR1
	PRKCA
	ITGAL
	ACHE
	PTGS2
	STAT3
	F2RL1
	ATP2A1
	PTPN11
	ATP12A
	TERT
	OPRD1
	OPRK1
	PCSK7
	HSD11B2
MOL001342	HSD11B1
	HMGCR

IL1B
CPA1
CYP19A1
AMPD2
ITGAL
PSEN2
ACE
PDE4D
IDO1
AKR1B1
AMPD3
EGFR
SRC
POLA1
PPP2CA
ATP12A
LTA4H
PRKCD
PRSS1
CTRC
AMPD1
PTGS2
GSR
JUN
NR3C1
CA2
CA1
CA14
CA4
CA13
CA5A
PTGDR2
CTNNB1
FNTA
CDC25B
PTPN1
MME
ITGAL
NR3C2
PRKCA
CTSA
PTGS1
PPARG
MMP3

	IMPDH1
MOL001343	PTGS2
	HSD11B1
	AMPD2
	HMGCR
	ACE
	IDO1
	PTGS1
	GSR
	LTB4R
	SLC22A12
	MMP3
	MME
	PREP
	PRSS1
	LTA4H
	NR3C1
	PARP1
	CTRC
	AMPD3
	CTSA
	CPA1
	CES2
	ABCB1
	SLC2A1
	ADCY1
	ITGB1
	NR3C2
	IMPDH1
	AKR1B1
	PRKCA
	MAPK8
	CTNNB1
	CSNK2A1
	PTGDR2
	PPP1CC
	PTGES
	ITGAL
	IMPDH2
	LANCL2
	GABRA1
	CREBBP
	ECE1
	ANPEP

	PIM1
	PIM2
	TYMS
	DHFR
	PYGL
	PYGM
	EDNRB
	TBXAS1
	ACE2
	WEE1
	JAK3
	HSD11B2
	PNP
	TTR
	BCAT2
	ACLY
MOL001344	HSD11B1
	HMGCR
	IL1B
	AMPD2
	POLA1
	IDO1
	CPA1
	LTA4H
	ACE
	CYP19A1
	ITGAL
	PYGL
	PYGM
	PTGS2
	GSR
	PSEN2
	PPP2CA
	SLC22A12
	AMPD3
	ATP12A
	PRKCD
	MME
	PRSS1
	CTRC
	PDE4D
	ECE1
	JUN
	PTGS1

	PTGDR2
	MAPK8
	ITGAL
	FNTA
	AMPD1
	AKR1B1
	ACE2
	NR3C2
	PRKCA
	FLT1
	KDR
	PARP1
	NR3C1
	MMP3
MOL001348	HSD11B1
	SAE1
	POLB
	AKR1B10
	NR1H4
	PTPN1
	PTGES
	TOP2A
	CDC25C
	CDC25A
	PTGS2
	AR
	CES2
	FNTA
	ACE
MOL001349	SAE1
	AKR1B10
	POLB
	NR1H4
	HSD11B1
	PTGES
	TOP2A
	CDC25C
	ACE
	AR
	CES2
	PTPN1
	FNTA
	PTGS2
	SLC22A12

	REN
MOL001350	HMGCR
	IL1B
	PPP2CA
	CXCR1
	ACE
	PRKCD
	NR3C1
	MME
	AKR1B1
	GSTM1
	IDO1
	NR3C2
	TERT
	GSR
	SLC22A12
	ATP2A1
	IMPDH1
	AMPD2
	AMPD3
	IMPDH2
	CPA1
	MMP3
	PCSK7
	EGFR
	SRC
	CTNNB1
	LTA4H
	PDE4D
	PTGS2
	F2RL1
	ITGAL
	PIM1
	PIM2
	ABCB1
	JUN
	KCNA3
	TTR
	PRSS1
	CTRC
	ANPEP
	ACLY
	ECE1
	IGF1R

	TEK
	F2
	PRKCA
	CSNK2A1
	SF3B3
	FNTA
	ITGB1
	PARP1
	FBP1
	ITGAL
MOL001351	HSD11B1
	F2
	PRSS1
	CTRC
	PTGS2
	TOP2A
	PTGES
	CPA1
	AMPD2
	PREP
	CYP17A1
	CDC25A
	CES2
	AMPD3
	VDR
	LTB4R
	MME
	CYP19A1
	PTPN1
	AMPD1
	HSD17B2
	OPRD1
	NR3C2
	HMGCR
	FLT1
	KDR
	TP53
	ECE1
	UGT2B7
	MAPK8
	PTGDR2
	IDO1
	PTGER4
	CCR9

	AR
	PYGL
	PYGM
	CTSA
	ITGB1
	ROCK2
	CYP26B1
	CYP26A1
MOL001352	HSD11B1
	AMPD2
	PTGS2
	SLC2A1
	ADCY1
	MME
	PRKCA
	TRPV4
	IDO1
	HMGCR
	LTB4R
	GLRA2
	ACE
	IMPDH2
	GSR
	CTNNB1
	IMPDH1
	PSEN2
	SLC22A12
	ABCB1
	PTGDR2
	NR3C1
	EGFR
	SRC
	MMP3
	AMPD3
	CPA1
	CTSA
	LTA4H
	PTGS1
	TBXAS1
	PYGL
	PTAFR
	PRKCD
	PARP1
	TLR9

	FOLH1
	MAPK8
	PRSS1
	PREP
	ITGAL
	CTRC
	ACLY
	ILK
	GABRA1
	CASP3
	TYMS
	ITGB1
	NR3C2
MOL001353	HSD11B1
	PRKCA
	AMPD2
	LTB4R
	ACE
	PTGS2
	GSR
	IDO1
	SLC22A12
	IMPDH1
	SLC2A1
	ADCY1
	MME
	LTA4H
	MMP3
	PRSS1
	IMPDH2
	AMPD3
	CTRC
	CPA1
	CES2
	CTNNB1
	ECE1
	TRPV4
	ITGB1
	EGFR
	SRC
	PTGS1
	PARP1
	SLC6A4
	ACLY

	HMGCR
	ITGAL
	PTGDR2
	ANPEP
	CTSA
	PYGL
	PYGM
	PIM1
	CSNK2A1
	PIM2
	IGF1R
	MAPK8
	TEK
	CREBBP
	CNR1
	CNR2
MOL001358	HMGCR
	PPP2CA
	IL1B
	AMPD2
	IDO1
	CPA1
	HSD11B1
	PPP1CC
	NR3C2
	MAPK8
	PRKCD
	OPRD1
	PTGDR2
	NR3C1
	ACE
	CYP19A1
	GSTM1
	LTA4H
	AMPD3
	ATP2A1
	PTGS2
	MMP3
	MME
	PARP1
	F2RL1
	PCSK7
	FNTA
	GSR

	PRSS1
	CTRC
	ITGAL
	AMPD1
	SF3B3
	PYGL
	PYGM
	ACE2
	IKBKB
	AKR1B1
	PTGS1
	CTSA
	UGT2B7
	PDE4D
	AR
	JUN
	KCNA3
	ROCK2
	P2RX3
	SLC22A12
	FLT1
	ITGB1
MOL001360	HSD11B1
	PTGS2
	PRSS1
	CTRC
	LTB4R
	AMPD2
	CES2
	CA14
	CA4
	CA13
	CA5A
	ACE
	MME
	AKR1B1
	IDO1
	AMPD3
	GSR
	HMGCR
	CPA1
	IMPDH1
	SLC22A12
	EGFR

	SRC
	CTNNB1
	IMPDH2
	LTA4H
	MMP3
	PRKCA
	NR3C1
	HSD11B2
	PTGDR2
	CTSA
	ECE1
	NR3C2
	ITGB1
	PTGS1
	AMPD1
MOL001361	HMGCR
	FNTA
	CXCR1
	IL1B
	PPP2CA
	PRKCD
	GSTM1
	CYP19A1
	AKR1B1
	ATP2A1
	PTGS2
	BCL2L1
	AR
	NR3C2
	TERT
	MME
	IMPDH2
	AMPD3
	AMPD2
	F2RL1
	PCSK7
	FBP1
	KCNA3
	JUN
	SELP
	SLC22A12
	CASP3
	CDC25C
	PRKCA

	CSNK2A1
	CTSA
	ANPEP
	LTA4H
	ECE1
	ACE
	FOLH1
	ACLY
	NR3C1
	PRSS1
	CTRC
	IMPDH1
	F2
	GSR
MOL001371	AKR1B1
	AKR1B10
	CA9
	CA12
	CA7
	CA6
	CA14
	CA5A
	CA13
	CA5B
	MET
	MAOB
	GSK3A
	GUSB
	MAPT
	TAS2R31
	CA2
	CA1
	ALOX5
	MMP9
	MMP1
	MMP2
	ALPG
	PLAA
	TERT
	MPI
	PTPN1
	ADORA3
	MTOR
	PIK3CG

CHRNA7
ALPL
HTR1A
EGFR
LCK
SCD
TYK2
CDK2
NOX4
MAPK14
CDK1
CHEK1
WEE1
PTPsigma
CDK4
ATP4B
RAF1
MYLK
PIM2
BRAF
MARK1
CDC25C
CDC25A
CDC25B
KDM1A
PDGFRB
KIT
GRM4
PTPN6
PTPN11
PLG
HDAC3
HDAC6
JAK1
HDAC1
ODC1
PTK6
MCL1
ACACB
TRPV1
CDK2
CDK9
AR
HDAC2

		HDAC7
		HDAC8
		ADAM17
		BCHE
		HSD17B3
		ESR1
		PTGS1
		DRD1
		DRD2
		SGK1
		DRD3
		SIGMAR1
		MPEG1
		TNF
		BACE2
		PLK1
		ROCK1
		PARP1
		BCL2
		ABCG2
		TRPM8
		DUSP3
		MELK
		PIM1
		MAPK1
		MAPKAPK2
Chi Ke	MOL002341	CA7
		CA12
		CA4
		CYP1B1
		CYP19A1
		TAS2R31
		ADORA1
		ADORA3
		ABCG2
		HSD17B1
		ESR2
		ESR1
		MAOB
		ABCC1
		SHBG
		CBR1
		MMP13
		PTGS1

BACE1
MMP12
GRM5
SRC
AKR1C3
PLA2G1B
KLK1
KLK2
CA3
CHRNA7
TERT
CA2
CA1
CA6
CA5A
POLB
SERPINE1
APP
PLA2G5
PLA2G10
PLG
NOX4
CA13
CA5B
ACHE
GUSB
CES1
SLC5A2
CES2
RXRA
KDR
GRM2
AKR1B1
CDK1
CCNE1
DUSP3
NTRK1
AURKA
CA9
TOP1
DNMT1
PGD
ST3GAL3
FUT7

	FUT4
	STAT1
	SQLE
	FFAR1
	PIM1
	PIM2
	PIM3
	MMP2
	IGF1R
	MMP9
	ODC1
	PARP1
	DNM1
	TNKS2
	TNKS
	MET
	DYRK1A
	ABCB1
	PPARG
	MAP4K4
	PLA2G2A
	CDK2
	PRMT1
	HSD17B2
	EDNRA
	BCL2
MOL004328	CYP19A1
	CA7
	ABCC1
	HSD17B1
	CA12
	SHBG
	CA4
	CYP1B1
	CBR1
	ESR1
	ESR2
	PTGS1
	MAOB
	ADORA1
	ADORA3
	ABCG2
	TAS2R31
	AKR1C3

PLA2G1B
GRM5
CES1
PPARG
CES2
SLC5A2
MMP12
POLB
MMP13
PLA2G2A
PLA2G5
PLA2G10
BACE1
CHRNA7
KLK1
KLK2
RXRA
SERPINE1
SRC
CA2
CA1
HSD17B14
KIT
KDR
FGFR1
MET
CA3
CA6
CA13
CA5B
CA5A
NQO2
CTSB
NOX4
AKR1B1
CLK1
DYRK1B
BCHE
CA9
ESRRA
ESRRB
CDK5
IGF1R
INSR

	DYRK1A
	HSD17B2
	IGFBP3
	MMP2
	YWHAG
	SIRT2
	F3
	PTGER1
	PTGER2
	PTGER3
	BCL2L1
	PIK3CB
	CYP2C9
	CYP3A4
	PIK3CA
	VCP
	PGF
	VEGFA
	EDNRA
	ERN1
	MMP3
	APP
	GSK3B
	BCL2
	MMP9
	WEE1
	LCK
	SYK
	CCNE1
	CCNE1
	CDK4
	AURKA
	SNCA
	ALOX12
	HNF4A
MOL005828	ABCG2
	AKR1B1
	OPRD1
	CYP1B1
	KIT
	OPRM1
	CA2
	ALOX5
	PTGS2

	NOX4
	NOS2
	ADORA3
	CA12
	ADORA1
	ADORA2A
MOL013381	CYP51A1
	HMGCR
	AR
	NPC1L1
	RORC
	CYP17A1
	NR1H3
	SREBF2
	ESR2
	CYP19A1
	ESR1
	SHBG
	SLC6A2
	PTPN1
	BCHE
	CHRM2
	SERPINA6
	RORA
	SLC6A4
	ACHE
	CYP2C19
	VDR
	NR1H2
	CDC25A
	GLRA1
	G6PD
	HSD11B1
	DHCR7
	PPARD
	SQLE
	CES2
	NR1I3
	CHRM4
	PTPN6
	PTPN2
	CCR1
	NR3C1
	NOS2

TACR1
HSD11B2
PTGES
POLB
